# Prebiotic formation of thioesters via cyclic anhydrides as a key step in the emergence of metabolism

**DOI:** 10.1038/s41598-025-91547-2

**Published:** 2025-02-27

**Authors:** Abdelkarim El Qami, Jorge Isaac Hilari, Véronique Blandin, Oscar Gayraud, Anne Milet, Yannick Vallée

**Affiliations:** Univ. Grenoble Alpes, CNRS, DCM, 38000 Grenoble, France

**Keywords:** Prebiotic chemistry, Abiogenesis, Protometabolism, rTCA cycle, 1,4-dicarboxylic acids, Thioesters, Chemistry, Organic chemistry, Origin of life, Theoretical chemistry

## Abstract

Thioesters are high-energy derivatives of carboxylic acids that are essential in the functioning of today’s living cells. Their central role argues in favor of their early introduction in the abiotic reaction network which led to the emergence of life on Earth. We propose that the first thioesters appeared during the establishment of the reverse tricarboxylic acid (rTCA) cycle, an effective metabolic cycle for the synthesis of organic molecules from CO_2_. Most of the acids in this cycle are 1,4-diacids. We show that the formation of a cyclic anhydride from aqueous solutions of succinic or citric acid is possible using drying conditions over silica, as it could happen in an evaporating pond. When these 1,4-diacids are dried in the presence of thiols, thioesters are obtained. Our experimental and theoretical results demonstrate that analogs of succinyl-CoA and citryl-CoA, thioesters from the rTCA cycle, can be produced. Such a process highlights the importance of 1,4-diacids, which would have been introduced in the metabolism then under construction because of their ability to form anhydrides and to be activated in the absence of triphosphates or of any other activating agent. At its beginning, the rTCA cycle should therefore be interpreted mainly as a “1,4-diacid cycle”.

## Introduction

The tricarboxylic acid cycle (TCA, Krebs cycle) occupies a central position in the metabolism of living cells^1^. Eight di- and triacids directly take part in it and, through these acids, it is the source of various other metabolites. The TCA cycle is exergonic. It is an oxidative process that degrades citric acid (a C6 molecule) into C4 diacids and two CO_2_ molecules. So, if already operative on the early Earth, it was not fitted for the building of organic molecules from CO_2_, a global reductive anabolic process. From a prebiotic point of view, the TCA cycle is not particularly interesting. In addition, the key molecule used to regenerate citrate (C6) from oxaloacetate (C4), thus closing the cycle, is a thioester, acetyl-CoA, itself produced from pyruvate via an oxidative decarboxylation reaction, so that another CO_2_ molecule must be lost.

**Fig. 1:**
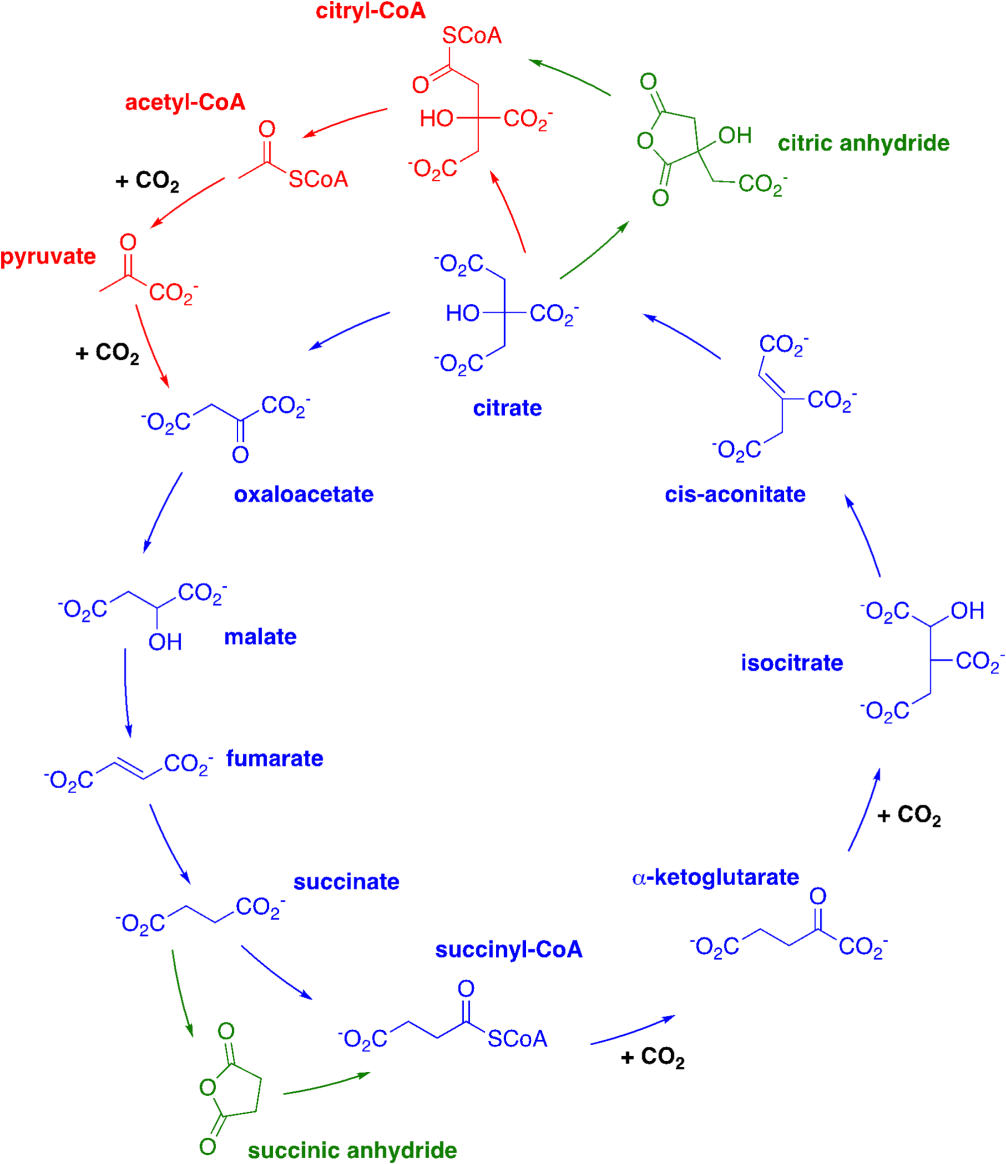
rTCA cycle. Blue, main cycle; red, horseshoe extension; green, anhydride hypothesis. The four integrated CO_2_ molecules are indicated.

For these reasons, the reverse TCA cycle (rTCA), which is active in some microorganisms today^2–5^, possesses much more potential as a key process at the origin of life (Fig. 1). Along the main part of this cycle (drawn in blue in Fig. 1), 2 CO_2_ molecules are consumed while C4 diacids are being elaborated into C6 triacids. Furthermore, in its “horseshoe” extension (in red), two other CO_2_ molecules react, with the C2 molecule, acetyl-CoA, being transformed into the C4 molecule, oxaloacetate. As a whole, in the complete (main + horseshoe) cycle, four CO_2_ molecules are integrated into organic matter. Indeed, several groups are working on this cycle due to its synthetic potential and major results have been published^6–11^. It is now well established that several steps of the rTCA cycle may have taken place in a world devoid of enzymes. This is especially the case for the reduction and dehydration steps.

However, many questions still remain unanswered, and among them the two following ones:

First, how were the involved thioesters produced? Solutions for the synthesis of thioesters in the TCA cycle have been proposed (oxidative decarboxylation reactions)^12,13^ but in the reverse cycle, thioesterification reactions of acids are needed. These are endergonic processes^14,15^ that in present biology are promoted by energy-rich triphosphates. Neither ATP nor any of its organic equivalents were present in the very early stages of proto-metabolism development. Their absence may possibly have been compensated by the presence of inorganic tri-(or di-)phosphates^16^. But what if these energy-rich inorganics were also absent or not usable here^17^? 

Second, the main rTCA cycle includes seven 1,4-diacids out of nine intermediates. Three of these 1,4-diacids (which actually are doubly 1,4-diacids) are also 1,5-diacids. Furthermore succinyl-CoA is a 1,4-diacid derivative. Only α-ketoglutarate is solely a 1,5-diacid. Monoacids are represented only in the horseshoe path: acetate, as a thioester, and pyruvate. Is there a chemical, prebiotic, explanation for the prevalence of 1,4-diacids in such an important metabolic cycle?

In this paper, we propose that, in an early version of the rTCA cycle, at least two 1,4-diacids were dehydrated in a self-activation process and that the anhydrides thus formed were opened by thiols, delivering thioesters (green parts of Fig. 1). On this basis, we argue that 1,4-diacids were “chosen” by life due to their ability to form five-membered cyclic anhydrides.

## Results

### Dehydration of succinic and citric acids

As anhydride formation is highly disfavored in water (e.g. in the bulk of the early ocean), the proposed process requires drying conditions. Such conditions have been frequently proposed for condensation reactions in prebiotic chemistry, for instance for the formation of amide bonds^18–20^. The idea is to mimic a primitive seashore submitted to tidal variation or an evaporating pond, for example near volcanoes. Our reactions were started in aqueous solutions and water was let to evaporate under heating. We did not perform a broad screening of possible solid supports. Among those we tried (Supplementary Table S1), only silica gel, an amorphous, mesoporous (6 nm pores) form of silica, chromatography quality, proved effective.

**Fig. 2:**
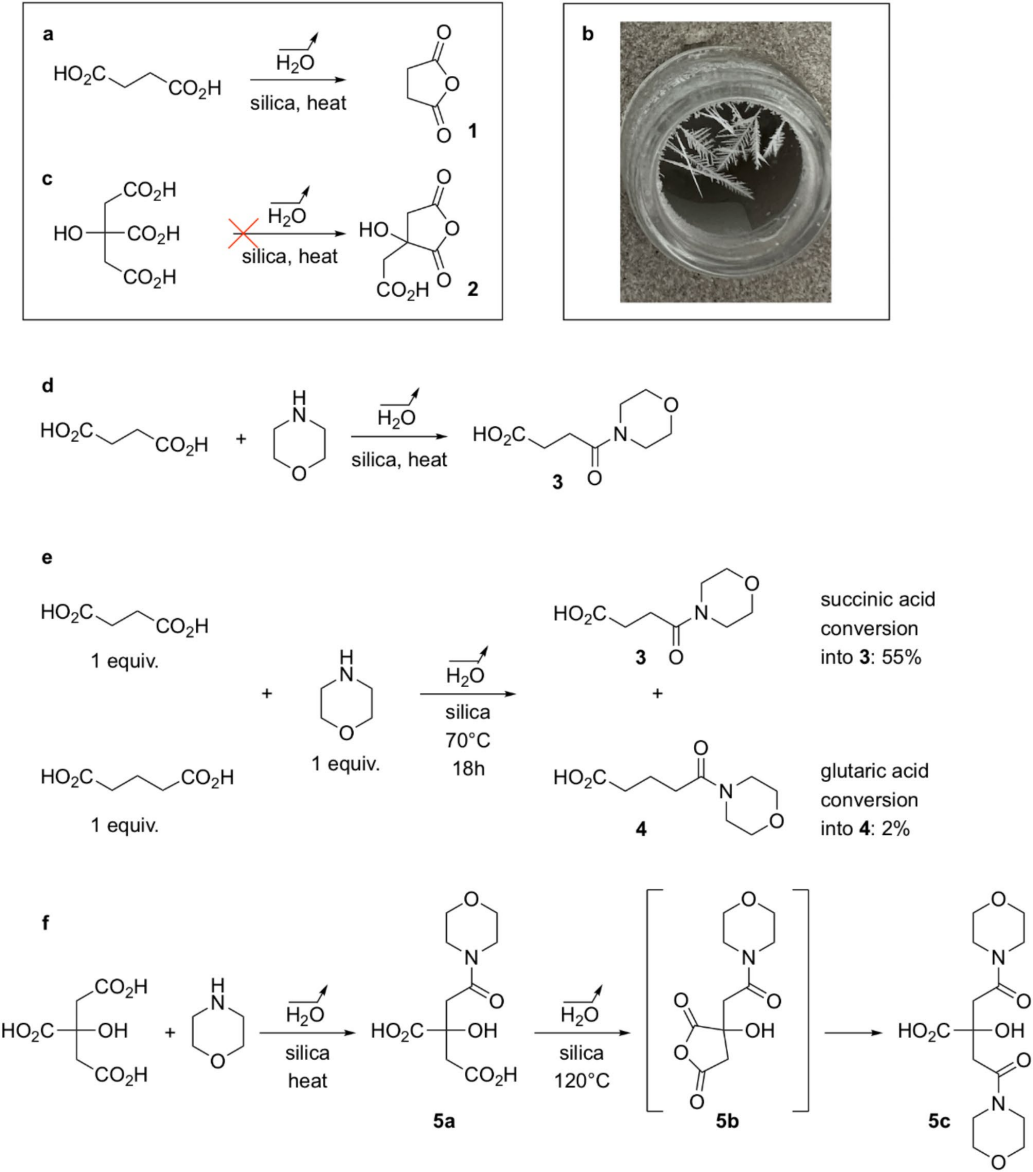
Experiments under drying conditions over silica, pointing towards the transient formation of anhydrides from 1,4-diacids. (**a**) From succinic acid, formation of anhydride **1** was evidenced. (**b**) Sublimate obtained from succinic acid dehydration at 120 °C. (**c**) Citric anhydride **2** was not detected from dehydration of citric acid. (**d**) Trapping experiments with morpholine for succinic acid: general scheme. The results detailed in the main text are consistent with a transient formation of **1** followed by opening with morpholine. (**e**) The result of a competitive reaction of succinic and glutaric acids with morpholine is consistent with a faster formation of five-membered ring anhydride. (**f**) Trapping experiments with morpholine for citric acid: general scheme. The results detailed in the main text are consistent with a transient formation of **2** followed by regioselective opening with morpholine. Higher temperatures favor a second dehydration/opening sequence.

The cyclization of 1,4-diacids into their corresponding anhydrides is a well-known process^21^. For instance, in the field of prebiotic chemistry, it has been proposed that the dehydration of aspartate residues at the end of certain peptide chains may have facilitated the elongation of these peptides^22,23^. Succinic acid itself can be dehydrated at elevated temperature^24^. In our case, when an aqueous solution of succinate was heated over silica till dryness (Fig. 2a), then maintained at 120 °C, a sublimate formed, consisting of the anhydride as identified by ^1^H NMR (anhydride/acid ratio 92:8) and X-ray diffraction analysis (Fig. 2b and Supplementary Table S1, entry 3; note that in the absence of silica, no transformation occurred: Supplementary Table S1, entry 1). At 100 °C, still with water evaporation but no sublimation, after six hours 3% anhydride **1** was found together with the acid in the remaining organic mixture (Supplementary Table S1, entry 8). Furthermore, when such experiments were run in the presence of morpholine, amide **3** was formed (Fig. 2d). Using 2 equiv. of morpholine at 100 °C, after 3 h, conversion into **3** reached 85% (Supplementary Table S2, entry 5). At 70 °C, after 56 h, it was 88% (6% after 6 h) (Supplementary Table S2, entries 12–13). It is noteworthy that the reaction of compound **3** (a monoacid) with morpholine to form the corresponding diamide was not observed, even at 100 °C. This argues in favor of a first amidation reaction occurring via the cyclic anhydride, and not via direct condensation of the amine with one of the carboxylic acid functions of succinic acid.

In order to demonstrate that 1,4-diacids are more reactive than other acids in such processes, competitive reactions between succinic and glutaric acids were run (Fig. 2e). At 70 °C, after 18 h, 55% conversion of succinic acid into **3** and 2% conversion of glutaric acid into **4** were obtained. This result is consistent with the formation of the six-membered ring anhydride of glutaric acid being much slower than the formation of succinic anhydride, a usual behavior for ring closings (5-membered quicker than 6-membered)^25^. Furthermore, no amide formation was observed from adipic acid under conditions (1 equiv. morpholine, 100 °C, 3–6 h) in which succinic acid gave amide **3** (Supplementary Table S2, entry 1: 3 h, 80% conversion).

It has been reported that when citric acid is dehydrated in the presence of acetic anhydride, the only formed cycle corresponds to a 1,4-diacid cyclisation^26^. However, when we heated citric acid over silica in conditions similar to the ones we used for succinic acid, no detectable amount of citric anhydride **2** could be observed in the ^1^H NMR spectrum of the organics remaining on silica (Fig. 2c). We used trapping experiments with morpholine to study the possible transient formation of the anhydride (Fig. 2f). Thus, using 2 equiv. of morpholine, amide **5a** represented 36% of the reaction mixture after 6 h at 100 °C (Supplementary Table S3, entry 12); 18% conversion was obtained after 6 h at 70 °C (Supplementary Table S3, entry 2). Remarkably, the only observed isomer corresponds to the regioselectivity required in the rTCA cycle. In some cases however, at 100 °C and even more at 120 °C, when using an excess of morpholine, the formation of diamide **5c** was also noticed (e.g. 120 °C, 3 h, 2 equiv. amine, 30% **5a**, 70% **5c**; Supplementary Table S3, entry 15), most probably resulting from transient formation of amido-anhydride **5b** (Fig. 2f). Even if the formation of amides directly from acids cannot be completely excluded^27,28^, it seems to us that all of our results point towards the essential involvement of anhydride intermediates.

### Reactions with CoM

**Fig. 3:**
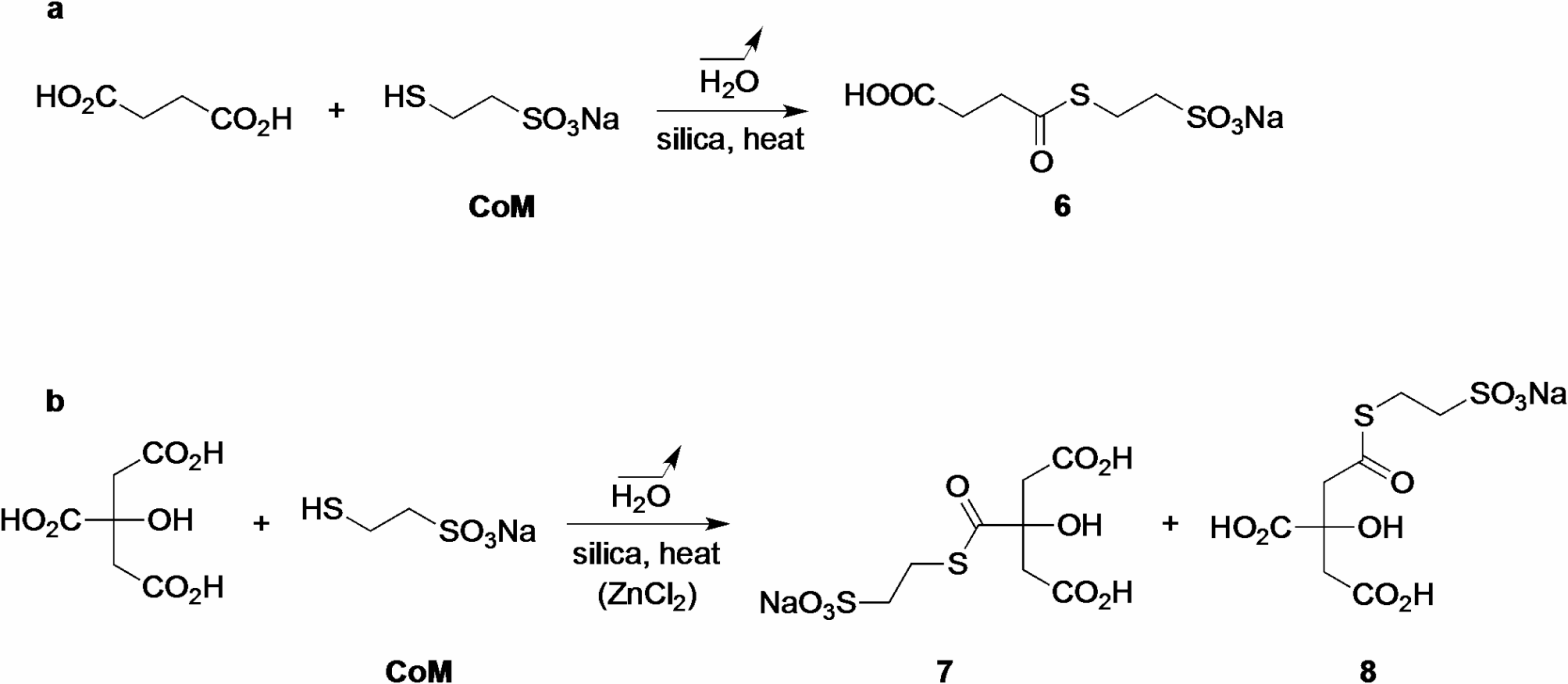
Drying conditions over silica allow the formation of thioesters from1,4-diacids: general schemes (**a**) from succinic acid; (**b**) from citric acid. In the latter case, two regioisomers could be observed; the presence of ZnCl_2_ favored thioester **8**.

We chose coenzyme M^29^, as it is highly soluble in water and non-volatile, to test the possibility to form thioesters from succinic and citric acids under drying conditions. When an aqueous solution of succinic acid was dried over silica in the presence of CoM, the expected thioester **6** was indeed formed (Fig. 3a). At 70 °C using 3 equiv. of CoM, 9% conversion into thioester was observed after 16 h (Table 1, entry 2). At 100 °C using only 1 equiv. of CoM, **6** was formed in about 20% conversion over the same period (Table 1, entries 3 and 6), using water or a sodium chloride solution simulating the salty ocean^30^. With higher amounts of CoM, higher conversions are reached (Table 1, entries 5 and 8). Wet-dry cycling^18,31–34^, whether as a series of rehydration/drying phases (Table 1, entries 4 and 7) or together with a new supply of thiol (Table 1, entry 8), did not provide significant effects on the conversion (see also Supplementary Table S4). Altogether, up to 36% conversion of succinic acid into CoM-thioester **6** in 18 h was observed.

**Table 1:** Formation of succinyl thioester **6** from succinic acid and CoM. For the extended data set see Supplementary Table S4.

Entry^a^	CoM (x equiv.)	Wet-dry cycles	T (°C)	Time (h)	Conversion (%)^b^ into 6
1	1	1	70	16	5
2	3	1	70	16	9
3	1	1	100	16	21
4	1	3	100	3 × 3	24
5	2	1	100	16	27
6^c^	1	1	100	16	22
7^c^	1	3	100	3 × 3	23
8	6 × 0.5	6	100	6 × 3	36

^a^Reaction conditions: succinic acid (100 mg), CoM (x equiv.), water (1 mL), silica (400 mg).

^b^Determined from the ^1^H NMR spectrum of the reaction mixture.

^c^Performed using 34 wt-% aqueous NaCl solution (1 mL) instead of water.

The main results with citric acid are summarized in Table 2. In contrast to the regioselectivity observed in the case of morpholine condensation, the only thioester we observed after the reaction of citric acid with CoM (1 equiv.) at 70 °C was **7** (Fig. 3b). After 72 h the obtained conversion was 9% (Table 2, entry 1) (nil after 16 h; Supplementary Table S5, entry 1). At 100 °C the reaction was more efficient: in 16 h, 14% of citric acid had reacted to give a 1:1 mixture of regioisomeric thioesters **7** and **8** (Table 2, entry 7). Note that **7** is not the suitable regioisomer for a postulated proto-rTCA cycle (Fig. 1).

**Table 2:** Formation of citryl thioesters **7** and **8** from citric acid and CoM. For the extended data set see Supplementary Table S5.

Entry^a^	Additive (x equiv.)	T (°C)	Time (h)	Conversion (%)^b^ into:	
				7	8
1	–	70	72	9	0
2	ZnCl_2_ (0.5)	70	72	8	5
3	ZnCl_2_ (1.0)	70	72	0	7
4^c^	ZnCl_2_ (1.0)	70	72	0	7
5	ZnCl_2_ (1.0)	100	16	0	27
6^c^	ZnCl_2_ (1.0)	100	16	0	28
7	–	100	16	7	7

^a^Reaction conditions: citric acid (100 mg), CoM (1 equiv.), water (1 mL), silica (400 mg).

^b^Estimated from the ^13^C NMR spectrum of the reaction mixture.

^c^Performed using 34 wt-% aqueous NaCl solution (1 mL) instead of water.

**Fig. 4:**
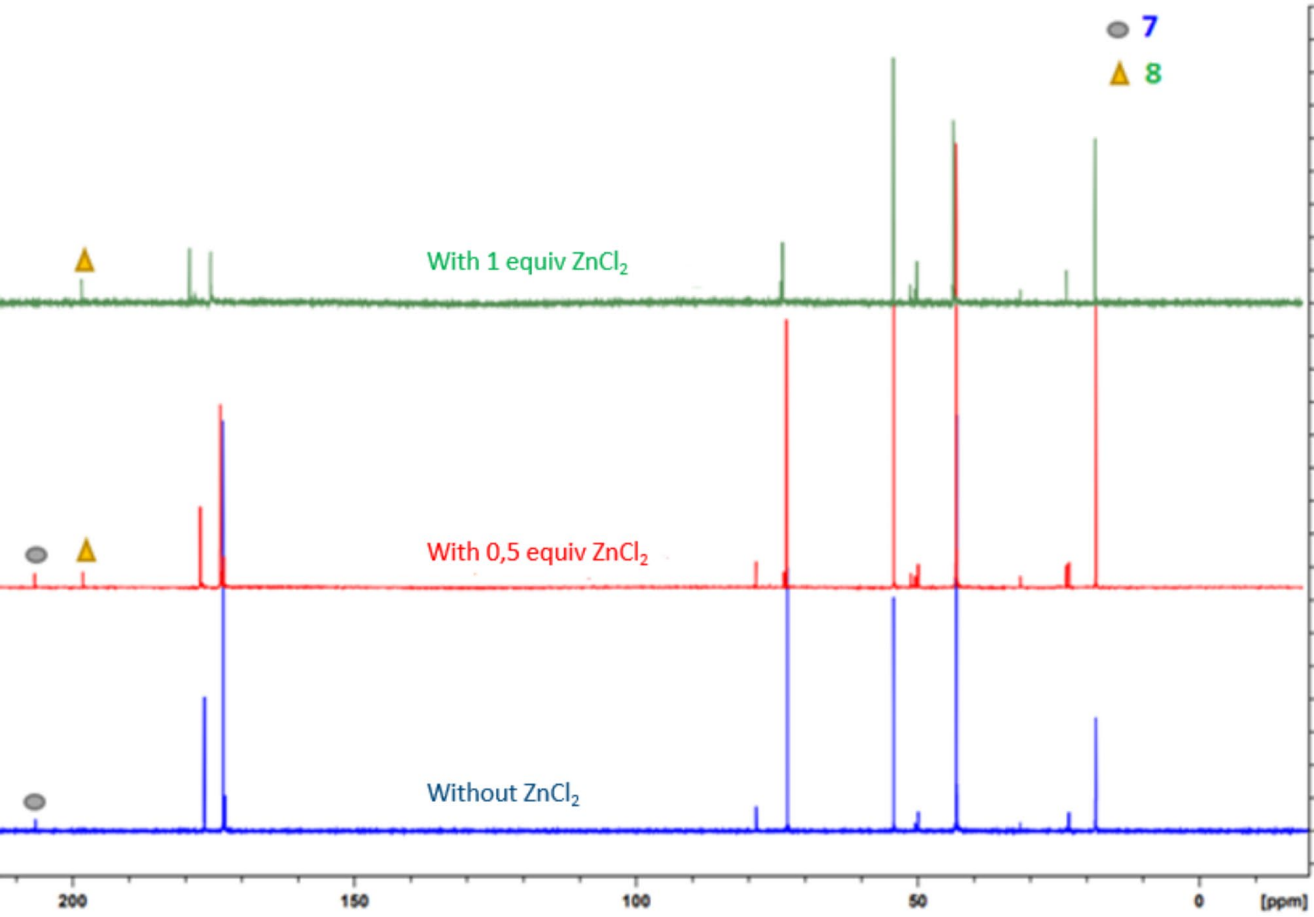
^13^C NMR spectra (D_2_O) showing the effect of ZnCl_2_ on the regioselectivity of thioester formation from dehydration of citric acid over silica in the presence of CoM. See also Supplementary Figures S19 and S21.

In order to invert the regioselectivity, and to obtain the suitable regioisomer **8**, various additives were tested (ZnCl_2_, CaCl_2_, MgCl_2_, BaCl_2_, FeCl_2_, FeCl_3_, Zn(OH)_2_; Supplementary Table S5). The only one that gave us satisfying results was ZnCl_2_ (Table 2, entries 2–6 and Fig. 4). For instance, after 16 h at 100 °C, **8** was the sole product formed, with 27% conversion from citric acid (Table 2, entry 5). The presence of NaCl did not affect conversions and regioselectivities (Table 2, entries 4 and 6).

### Reactions with Na_2_S

The first nucleophilic sulfur species on the early Earth must have been H_2_S, present in water as its anions HS^−^ and S^=^ (H_2_S itself is poorly soluble in water)^35^. In order to minimize security risks, and knowing that 5-membered cyclic anhydrides form easily, we reacted sulfide anions directly on preformed anhydrides, thus avoiding the need to heat H_2_S salts in water (which would have formed highly toxic H_2_S gas).

**Fig. 5:**
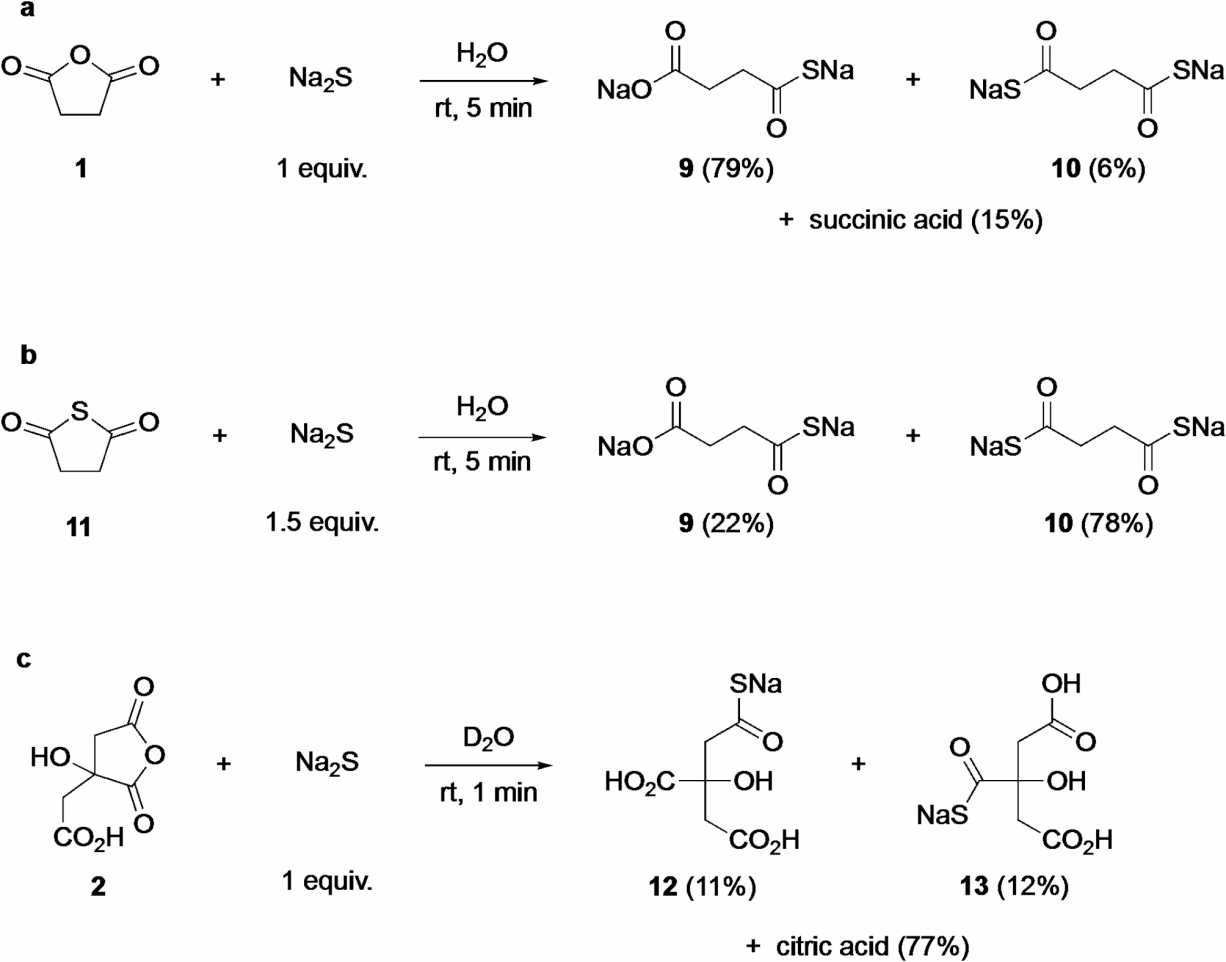
Sulfide anion opening of anhydrides. (**a**) Reaction with succinic anhydride leads mainly to thiocarboxylate **9**. (**b**) A transient formation of thioanhydride **11** accounts for the presence of dithiocarboxylate **10**. (**c**) Reaction with citric anhydride is not regioselective and accompanied by hydrolysis.

When succinic anhydride **1** was added to a solution of 1 equiv. Na_2_S in water (Fig. 5a), a rapid, complete conversion of **1** occurred, with some concomitant hydrolysis (15% of diacid) and thiocarboxylate **9** as the major product (79%). We also noticed the presence of dithiocarboxylate **10** (6% determined by ^1^H NMR) in the reaction mixture. We interpreted the formation of this dithio compound as a result of the transient formation of some thioanhydride **11** whose opening would have led to **10**. Indeed, in a test experiment (Fig. 5b), preformed **11**^36^ was reacted with Na_2_S (1.5 equiv.) and **10** was obtained in 78% yield.

Under similar conditions with citric acid anhydride **2** (Fig. 5c), the formation of regioisomeric thiocarboxylates **12** and **13** was evidenced by ^13^C NMR (Supplementary Fig. S27). Hydrolysis of citric anhydride and/or its thiocarboxylate derivatives proved however very fast, especially in the presence of ZnCl_2_, in which case only citric acid was observed.

## Discussion

Our results demonstrate that the step of the rTCA cycle from succinate to succinyl thioester could have occurred on early Earth under drying conditions. As the reaction requires quite a long time to reach reasonable conversions, this process may well be more consistent with an “evaporating pond” scenario (in which the pond may stay dry for a considerable duration before being refilled with water)^37^ than with a coastal scenario for which the duration of the dry period is limited by the periodicity of the tides^38^. Of course, it is not possible to be sure that the conditions we used correspond precisely to those which could prevail on the primitive Earth around and in a warm pond (or even if such ponds existed). It is, however, probable that the components which are necessary for our hypothesis were present at some point. Regarding silica, it was probably, as today^39^, the most abundant solid on the surface of the primitive Earth and silica gel could form from rocks by a dissolution-precipitation process^40,41^. This gel was porous and provided a surface of choice to promote organic reactions^40,41^. The possibly lower activity of this primitive silica, compared to the gel we used, could be compensated by the time available, which far exceeded the times envisaged for laboratory reactions.

**Fig. 6:**
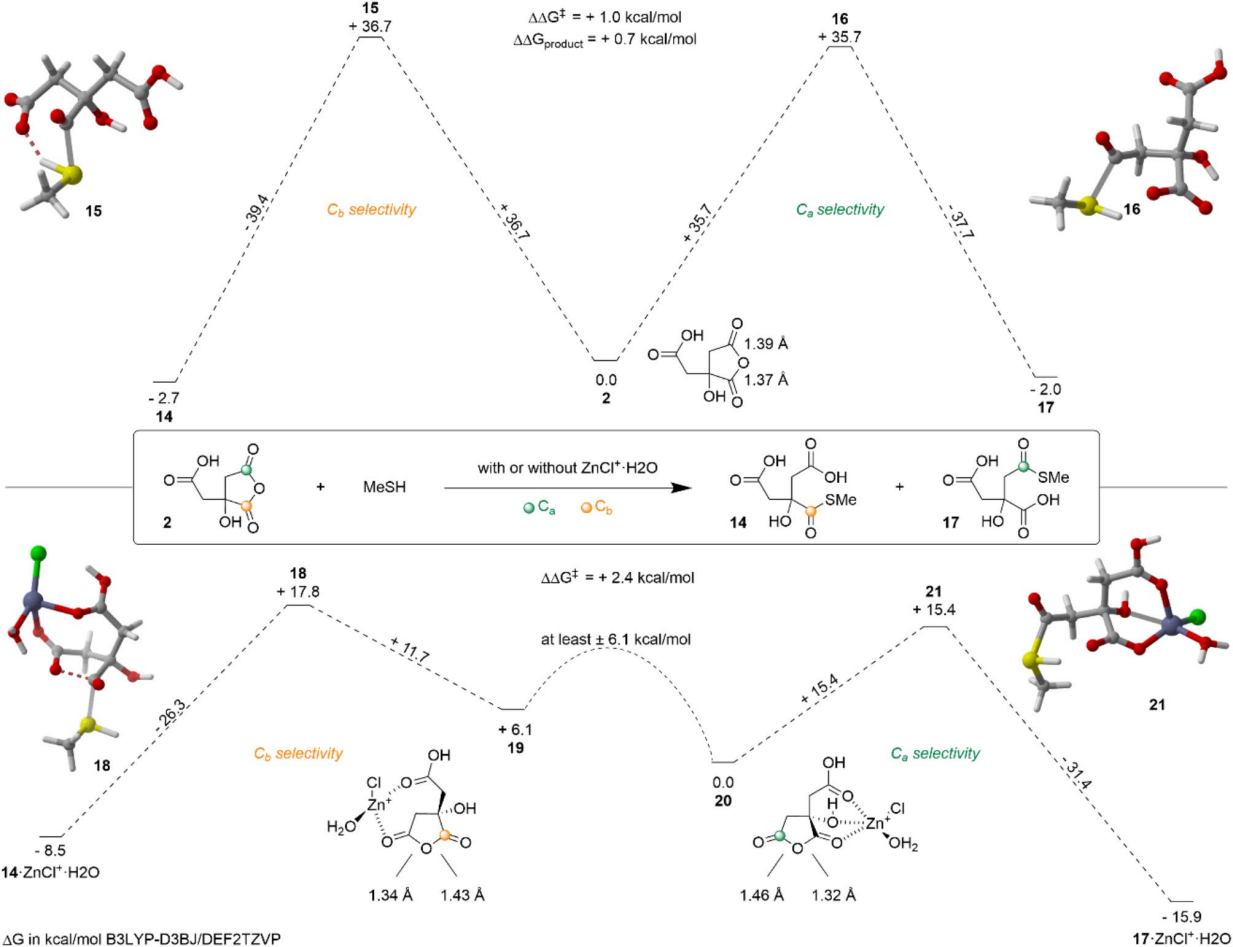
Energy profile of the nucleophilic addition of MeSH on citric anhydride **2** with or without ZnCl^+^·H_2_O as Lewis acid. The Gibbs free energies relative to the starting materials are given in kcal/mol.

The case of citric acid is more complex. Calculations based on density-functional theory were conducted in order to understand the obtained regioselectivities; the lowest pathways for each product, with or without a Lewis acid, are gathered on Fig. 6. The reaction was modeled using citric anhydride **2** and methanethiol, MeSH, as the model nucleophile (Fig. 6-top). The activation barrier of the attack on C_a_ (+ 35.7 kcal/mol) is stabilized by + 1.0 kcal/mol compared to the attack on C_b_ (+ 36.7 kcal/mol), in line with a lower energy of the antibonding orbital of O-C_a_ compared to O-C_b_: Δ(E(BD*_O−Ca_) - E(BD*_O−Cb_)) = − 0.67 eV. Experimentally, after 72 h at 70 °C, only **7** was detected (Table 2, Entry 1), in accordance with the observation that product **14** is + 0.7 kcal/mol more stable than **17**.

The investigation was pursued using ZnCl^+^·H₂O as a model of the Lewis acid (Fig. 6-bottom). Two coordination sites, **19** and **20**, were observed, with **20** being 6.1 kcal/mol more stable than **19**. Despite an extensive investigation into the carbonyl closest to the metal complex (C_a_ in case **19** and C_b_ in case **20**), no transition states (TS) were identified. With regard to the carbonyl furthest from the coordination site, C_b_ in the case of **19**, an activation barrier of + 11.7 kcal/mol was observed for a *trans* attack of MeSH relative to the Zn center (TS **18**). The O-C_b_ bond exhibits a longer bond length, Δ(B_O−Ca_ - B_O−Cb_) = − 0.09 Å, and a stabilized antibonding orbital, Δ(E(BD*_O−Ca_) - E(BD*_O−Cb_)) = + 2.6 eV. For C_a_ in **20**, an activation barrier of + 15.4 kcal/mol was observed for a *trans* attack, TS **21**. The O-C_a_ bond is longer, Δ(B_O−Ca_ - B_O−Cb_) = + 0.14 Å, and the antibonding orbital is more stabilized, Δ(E(BD*_O−Ca_) - E(BD*_O−Cb_)) = − 4.5 eV. Given the energy difference between **19** and **20** of + 6.1 kcal/mol, a + 2.4 kcal/mol gap is calculated between TS **18** and TS **21**, in accordance with the observed experimental selectivity at 100 °C after 16 h in the presence of 1 equiv. ZnCl_2_ (only **8** is formed, Table 2, Entry 5).

It is thus possible to obtain selectively the citryl thioester regioisomer in line with rTCA cycle intermediates in the presence of Zn^2+^ cations. Obviously, sufficient availability of zinc on the early Earth can be questioned. Nowadays the concentration of zinc in the oceans is about 10^− 8^ g/L^42,43^ and in the terrestrial crust about 70.10^− 6^ kg/kg^39,44^, but it is relatively abundant in living cells, about 10^− 3^ g/L^43^. In the human body, the Fe/Cu/Zn ratio is 2:0.03:1, whereas it is 800:1:1 in the terrestrial crust^45^. Therefore it appears that at some stage zinc has been selected^43,46^. Was it used in primitive rTCA cycles? In any case, it is not found in the catalytic site of the enzymes used today for these reactions (but the mechanism they employ does not involve a cyclic anhydride intermediate either)^47^. Moreover, what seems important to us beyond the specific use of zinc is that the correct regioselectivity is possible to achieve, and therefore that the citrate could have been transformed into an analog of citryl-CoA thioester on the primitive Earth under drying conditions, providing at least some emerged lands existed 4 billion years ago, which seems to be probable^40,48–50^.

It is, however, highly unlikely that these lands (from small islands to continents at the beginning of their growth) could have been, even in very specific places, thoroughly dry. Under a generally humid atmosphere, the soils had to remain impregnated with water. This is why we did not seek to use hyper-dry minerals. Under the conditions we applied, silica retains some water and silanol groups are present at its surface (especially of course at 70 °C, but also at 100 °C and even 120 °C)^51–54^. The fact that we observed the dehydration of diacids in environments that were not completely dry makes our hypothesis more plausible. This is also why we introduced a water molecule into some of our theoretical models. As in other studies, a catalytic effect of silica was noted (compare Supplementary Figures S28,S29 and S33,S34), which could be explained by the formation of hydrogen bonds between silica and the substrate^53,55–57^.

Thioesters are crucial high-energy intermediates in the present-day metabolism and have been proposed to be key players in the development of a proto-metabolism on Earth (de Duve’s thioester world)^58^. Their non-enzymatic synthesis from acids is far from obvious. Today they are obtained by means of the transient formation of mixed carboxylic-phosphoric anhydrides^59,60^. Being their own activator, 1,4-diacids have the ability to form intramolecularly an activated form. As this reaction is intramolecular, it is much quicker than similar reaction from monoacids. It is also quicker than anhydride formation from 1,5-diacids. We propose that this is why 1,4-diacids allowed the establishment of a proto-rTCA cycle on the primitive Earth and by consequence why they are so important in today’s rTCA and TCA cycles. In a way, rather than being a tricarboxylic acid cycle (only three of the nine acids involved in the main rTCA cycle (in blue Fig. 1) are triacids), the rTCA cycle is a 1,4-diacid cycle (seven acids in the main rTCA cycle are 1,4-diacids), and the same applies to the direct Krebs cycle. A reasonable amount of heat (about 70–100 °C) was the only necessary energy, provided that at least some islands emerged from the oceans. In the framework of our hypothesis, succinic and citric acids (and maybe other 1,4-diacids), because they were able to form cyclic anhydrides, were the first acids able to be transformed into thioesters. In the debate between a possible thioester world and an acylphosphate world at the origin of life^61^, our proposal is positioned on the side of the thioester world.

In the presented scenario, 1,4-diacid cyclic anhydrides were the first formed high-energy organic derivatives and citryl thioesters were the precursors of thioacetates (horse-shoe extension of rTCA cycle, in red Fig. 1), themselves precursors of fatty acids. Added to the results of previous studies^6^, these findings demonstrate that a non-enzymatic rTCA cycle could have been effective on the early Earth at the very beginning of a (proto-) metabolism network. The steps following these thioester formations still have to be investigated. For the citryl thioester, it is a retroaldol reaction leading to oxaloacetate and thioacetate. For thiosuccinate, it is a reductive carboxylation to ketoglutarate followed by a second carboxylation and a reduction leading to isocitrate.

## Methods

### Experimental

Reagents and solvents were purchased from commercial sources such as Sigma-Aldrich, Alfa Aesar or VWR CHEMICALS and used without further purification. Citric acid anhydride^26^ and thiolane-2,5-dione^36^ were synthesized using known procedures. Authentic samples of 4-morpholino-4-oxobutanoic acid **3**^62^, 5-morpholino-5-oxopentanoic acid **4**^63^ and 2-hydroxy-2-(2-morpholino-2-oxoethyl)succinic acid **5a** were prepared for comparison purposes by reacting morpholine with, respectively, succinic anhydride, glutaric anhydride and citric acid anhydride. Diacid dehydrations were performed over Macherey Nagel Silica Gel 60 (0.063–0.2 mm / 70–230 mesh ASTM). The ultrapure water used throughout the experiments was obtained from an ELGA lab water purification system. pH of the reaction mixtures were estimated by means of pH strips. NMR spectra were recorded at 298 K on Bruker Avance III 400 or Avance III 500 spectrometer at, respectively, 400 MHz or 500 MHz for ^1^H NMR, and 100 or 125 MHz for ^13^C NMR. They were recorded in acetone-*d*_*6*_ (calibration δ_H_ = 2.05 ppm, δ_C_ = 206.3 ppm) or D_2_O (calibration δ_H_ = 4.79 ppm). Multiplicities are declared as follows: s (singlet), d (doublet), dd (doublet of doublet), t (triplet), m (multiplet). Coupling constants (*J*) are given in Hertz. ^1^H and ^13^C resonance assignments were performed using conventional 1D and 2D techniques. Low-resolution mass spectra were recorded on a Bruker amaZon speed spectrometer. High-resolution mass spectra (HRMS) were recorded on a Thermo Scientific LTQ Orbitrap XL spectrometer. Crystal data (sublimate from the dehydration of succinic acid) were collected on a Bruker AXS Incoatec-Enraf-Nonius kappa APEX II diffractometer working at the MoKα wavelength (0.71073 Å) and at 200 K.

#### Typical dehydration procedure: reactions with CoM

A 20 mL glass vial was charged with succinic or citric acid (100 mg), water (1 mL), CoM (1 equiv.) and silica (400 mg). The pH of the solutions were ≈ 1–2. After manual stirring was performed, the vial was placed in an oil bath (70–100 °C). After a time, the residues were washed with water (2 × 5 mL) on a filter funnel. The filtrate was then analyzed; the formation of product(s) was evidenced by ^1^H NMR analysis (with water suppression), ^13^C NMR and HRMS analyses. Conversions were determined from the ^1^H NMR (succinic acid series) spectrum or estimated from the ^13^C NMR (citric acid series) spectrum; no standard was added.

#### Wet-dry cycles

After 3 h of heating at 100 °C, silica was visibly dry. Water (1 mL) and CoM (x equiv.) were then added, the vial was manually stirred and heating was resumed for three more hours.

#### Note

All reactions involving sodium sulfide were carefully carried out in a fume hood.

For each part of the study, the extended data set, detailed experimental procedures, products characterization and representative spectra are provided in Supplementary Information.

### Computational

A reported methodology FASTCAR^64^ was applied to secure the lowest transition state (TS) and products. Briefly, it begins with the use of CREST, an automatic conformational search developed by Grimme and coworkers^65^, with an arbitrary initial TS. The conformers ensemble found was pruned using sPyRMSD^66^. The remaining geometries were then optimized at the B3LYP-D3BJ/DEF2SVP level. Any redundant or incorrect geometries were discarded, and the final ensemble was optimized at the B3LYP-D3BJ/DEF2TZVP level. All optimizations performed using Gaussian were conducted without constraints^67^. In order to ensure that the stationary point was of the appropriate nature in both cases, vibrational frequencies were systematically computed in the case of the transition state search and at the end of the search regarding the minimum. Finally, intrinsic reaction coordinate (IRC) calculations were performed to verify that the transition states connect the reagents and products. Further secondary reaction pathways and a discussion of the Zn species nature (ZnCl^+^.H_2_O vs. ZnCl^+^.MeSH) and of the role of silica in the reaction are included in the Supplementary Information (Supplementary Fig. S28–S34). A two-step process (C-S bond formation followed by a proton transfer) was hypothesized and checked through potential energy surface (PES) scan calculation, but no intermediates were found. Molecular structure images were generated using the CYLview2.0 software^68^.

## Electronic supplementary material

Below is the link to the electronic supplementary material.

Supplementary Material 1

## Data Availability

All data for this work are available in the main text or supplementary materials.
