# Supplementary material for: Prebiotic formation of thioesters via cyclic anhydrides as a key step in the emergence of metabolism
Source: Sci Rep. 2025 Feb 27;15:7039. doi: 10.1038/s41598-025-91547-2 (PMC11868630; doi:10.1038/s41598-025-91547-2)
Supplement: Supplementary file 1 — Supplementary Material 1 [file 41598_2025_91547_MOESM1_ESM.pdf]

Supplementary Information for

Prebiotic formation of thioesters via cyclic anhydrides as a key step in the emergence of metabolism

Abdelkarim El Qami,[1] Jorge Hilari,[1] Véronique Blandin,[1,*] Oscar Gayraud,[1] Anne Milet[1] & Yannick Vallée.[1]

[1] Univ. Grenoble Alpes, CNRS, DCM, 38000 Grenoble, France.

Corresponding authors: Véronique Blandin and Yannick Vallée.

Email: veronique.blandin@univ-grenoble-alpes.fr; yannick.vallee@univ-grenoble-alpes.fr

This PDF file includes:

Supplementary text
Supplementary Figures S1 to S34
Supplementary Tables S1 to S7
SI References

Contents – Part I: Experimental

1. General considerations S6

2. SI references (Part I) S6

3. Dehydration of succinic acid into anhydride 1 .... S7

Table S1. Dehydration of succinic acid into anhydride 1 over a mineral surface. **S7**

Example procedure (Table S1, Entry 2) **S7**

Fig. S1. Dehydration of succinic acid into anhydride 1: ^{1}H NMR spectrum (acetone-d_6) of organics recovered in the vial (Table S1, Entry 2). **S8**

Sublimate formation and analysis (Table S1, Entry 3) **S8**

4. Dehydration of succinic acid in the presence of morpholine S9

Table S2. Dehydration of succinic acid over silica in the presence of morpholine. **S9**

Example procedure (Table S2, Entry 1) **S10**

Fig. S2. ^{1}H NMR spectrum (acetone-d_6) of purified compound 3. .... **S10**

Fig. S3. ^{13}C NMR spectrum (acetone-d_6) of purified compound 3. .... **S11**

Fig. S4. HRMS spectrum of purified compound 3. .... **S11**

Fig. S5. ^{1}H NMR spectrum (D_2O) of the reaction mixture for the dehydration of succinic acid over silica in the presence of 2 equivalents morpholine (Table S2, Entry 3). **S12**

5. Competitive reactions between succinic acid and glutaric acid S13

Fig. S6. Competitive dehydrations of succinic and glutaric acid over silica in the presence of morpholine (1 equiv.); ^{1}H NMR spectrum (D_2O) of the reaction mixture. **S13**

6. Dehydration of citric acid in the presence of morpholine S14

Table S3. Dehydration of citric acid over silica in the presence of morpholine. **S14**

Example procedure (Table S3, Entry 5) **S15**

Fig. S7. ^{1}H NMR spectrum (D_2O) of purified compound 5a. **S15**

Fig. S8. ^{13}C NMR spectrum (D_2O) of purified compound 5a. **S16**

Fig. S9. HRMS spectrum of the reaction mixture for the dehydration of citric acid over silica in the presence of 1 equivalent morpholine (Table S3, Entry 5). **S17**

Fig. S10. ^{1}H NMR spectrum (D_2O) of the reaction mixture for the dehydration of citric acid over silica in the presence of 1 equivalent morpholine (Table S3, Entry 5). **S17**

Example procedure (Table S3, Entry 12) **S18**

Fig. S11. ^{1}H NMR spectrum (D_2O) of purified compound 5c. **S18**

Fig. S12. HRMS spectrum of the reaction mixture for the dehydration of citric acid over silica in the presence of 2 equivalents morpholine (Table S3, Entry 12). **S19**

Fig. S13. ^{1}H NMR spectrum (D_2O) of the reaction mixture for the dehydration of citric acid over silica in the presence of 2 equivalents morpholine (Table S3, Entry 12). **S19**

7. Dehydration of succinic acid in the presence of Coenzyme M S20

Reference NMR spectra **S20**

Fig. S14. ^{1}H NMR spectrum obtained from the reaction of succinic anhydride with CoM in D_2O. **S20**

Fig. S15. ^{13}C NMR spectrum obtained from the reaction of succinic anhydride with CoM in D_2O. **S21**

Table S4. Dehydration of succinic acid over silica in the presence of CoM. **S22**

Example procedure (Table S4, Entry 21) **S24**

Fig. S16. ^{1}H NMR spectrum (D_2O) of the reaction mixture for the dehydration of succinic acid over silica in the presence of 1 equivalent CoM (Table S4, Entry 21). **S24**

Fig. S17. ^{13}C NMR spectrum (D_2O) of the reaction mixture for the dehydration of succinic acid over silica in the presence of 1 equivalent CoM (Table S4, Entry 21). **S25**

Fig. S18. HRMS spectrum of the reaction mixture for the dehydration of succinic acid over silica in the presence of 1 equivalent CoM (Table S4, Entry 3). **S25**

8. Dehydration of citric acid in the presence of Coenzyme M S26

Table S5. Dehydration of citric acid over silica in the presence of CoM. **S26**

Example procedure without additive (Table S5, Entry 2) **S27**

Fig. S19. ^{13}C NMR spectrum (D_2O) of the reaction mixture for the dehydration of citric acid over silica in the presence of 1 equivalent CoM (Table S5, Entry 2). **S28**

Fig. S20. HRMS spectrum of the reaction mixture for the dehydration of citric acid over silica in the presence of 1 equivalent CoM (Table S5, Entry 2). **S28**

Example procedure with additive (Table S5, Entry 18) **S29**

Fig. S21. ^{13}C NMR spectrum (D_2O) of the reaction mixture for the dehydration of citric acid over silica in the presence of 1 equivalent CoM (Table S5, Entry 18). **S29**

9. Reaction of succinic anhydride 1 with sodium sulfide S30

Table S6. Product distribution for the reaction of succinic anhydride 1 with Na_2S. **S30**

Example procedure (Table S6, Entry 3) **S30**

Fig. S22. ^{1}H NMR spectrum (D_2O) of the reaction mixture of succinic anhydride 1 with 2 equivalents of Na_2S in water (Table S6, Entry 3). **S31**

Fig. S23. ^{13}C NMR spectrum (D_2O) of the reaction mixture of succinic anhydride 1 with 2 equivalents of Na_2S in water (Table S6, Entry 3). **S32**

Fig. S24. ^{13}C NMR spectra (D_2O) of thioacetic acid (contaminated with acetic acid): (*bottom*) recorded directly (pH ≈ 4); (*top*) recorded after addition of a solution of NaOH (1M) up to pH ≈ 12. .... **S32**

Table S7. Reaction of succinic thioanhydride 11 with Na_2S. **S33**

Example procedure (Table S7, Entry 2) **S33**

Fig. S25. ^{1}H NMR spectrum (D_2O) of the reaction mixture of succinic thioanhydride 11 with 1.5 equivalents of Na_2S in water (Table S7, Entry 2). **S34**

Fig. S26. ^{13}C NMR spectrum (D_2O) of the reaction mixture of succinic thioanhydride 11 with 1.5 equivalents of Na_2S in water (Table S7, Entry 2). **S34**

10. Reaction of citric anhydride 2 with sodium sulfide S35

Fig. S27. ^{13}C NMR spectrum (D_2O) of the reaction mixture of citric anhydride 2 with 1 equivalent of Na_2S. **S35**

Contents – Part II: Computational

11. Computational details **S36**

12. Nucleophilic addition of MeSH to citric anhydride without Lewis acid **S37**

Fig. S28. Nucleophilic addition of MeSH to the C_a site of citric anhydride without Lewis acid.......... **S37**

Fig. S29. Nucleophilic addition of MeSH to the C_b site of citric anhydride without Lewis acid.......... **S45**

13. Nucleophilic addition of MeSH to citric anhydride with $Zn^+Cl.H_2O$ as Lewis acid **S52**

Fig. S30. Nucleophilic addition of MeSH to the C_a site of citric anhydride with $ZnCl^+H_2O$ as Lewis acid. . **S52**

Fig. S31. Nucleophilic addition of MeSH to the C_b site of citric anhydride with $ZnCl^+H_2O$ as Lewis acid. . **S61**

14. Catalyst choice **S70**

Fig. S32. SI_{DFT} 1 complex with $Zn^+Cl.L$; L being either H_2O or MeSH. **S70**

Fig. S33. Nucleophilic addition of MeSH to the C_a site of citric anhydride with a model of silica as catalyst. **S76**

Fig. S34. Nucleophilic addition of MeSH to the C_b site of citric anhydride with a model of silica as catalyst. **S92**

SI references (Part II) **S107**

Part I: Experimental

1. General considerations

Reagents and solvents were purchased from commercial sources such as Sigma-Aldrich, Alfa Aesar or VWR CHEMICALS and used without further purification. Citric acid anhydride[1] and thiolane-2,5-dione[2] were synthesized using known procedures. Authentic samples of 4-morpholino-4-oxobutanoic acid **3**[3], 5-morpholino-5-oxopentanoic acid **4**[4] and 2-hydroxy-2-(2-morpholino-2-oxoethyl)succinic acid **5a** were prepared for comparison purposes by reacting morpholine with, respectively, succinic anhydride, glutaric anhydride and citric acid anhydride. Diacid dehydrations were performed over Macherey Nagel Silica Gel 60 (0.063-0.2 mm / 70-230 mesh ASTM). The ultrapure water used throughout the experiments was obtained from an ELGA lab water purification system. The pH of the reaction mixtures were estimated by means of pH strips. NMR spectra were recorded at 298K on Bruker Avance III 400 or Avance III 500 spectrometer at, respectively, 400 MHz or 500 MHz for ^{1}H NMR, and 100 or 125 MHz for ^{13}C NMR. They were recorded in acetone-d_6 (calibration δ_H = 2.05 ppm, δ_C = 206.3 ppm) or D_2O (calibration δ_H = 4.79 ppm). No standard was added. Multiplicities are declared as follows: s (singlet), d (doublet), dd (doublet of doublet), t (triplet), m (multiplet). Coupling constants (*J*) are given in Hertz. ^{1}H and ^{13}C resonance assignments were performed using conventional 1D and 2D techniques. Low resolution mass spectra were recorded on a Bruker amaZon speed spectrometer. High-resolution mass spectra (HRMS) were recorded on a Thermo Scientific LTQ Orbitrap XL spectrometer. Crystal data (sublimate from the dehydration of succinic acid) were collected on a Bruker AXS Incoatec-Enraf-Nonius kappa APEX II diffractometer working at the MoKα wavelength (0.71073 Å) and at 200K.

2. SI references (Part I)

1. Repta, A. J. & Higuchi, T. Synthesis, isolation, and some chemistry of citric acid anhydride. *J. Pharm. Sci.* **58**, 1110-1114 (1969).
2. Kates, M. J. & Schauble, J. H. Synthesis of small-medium ring thioanhydrides. *J. Heterocyclic Chem.* **32**, 971-978 (1995).
3. Milanos, L. et al. Discovery and characterization of biased allosteric agonists of the chemokine receptor CXCR3. *J. Med. Chem.* **59**, 2222-2243 (2016).
4. Astiel, L. D. & Fleisch, J. H. Leukotriene antagonists for use in the treatment or prevention of Alzheimer's disease. Eur. patent EP0743064A1 (1996).
5. Ferretti, V., Gilli, P. & Gavezzotti, A. *Chem. Eur. J.* **8,** 1710-1718 (2002).

3. Dehydration of succinic acid into anhydride 1

Table S1. Dehydration of succinic acid into anhydride **1** over a mineral surface.[a]

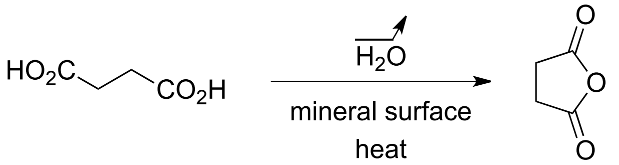

Entry	Mineral surface	T (°C)	Time (h)	Amount of 1 (%) in the organics[b]
1	–	120	6	0
2	Silica	120	6	37
3	Silica	120	6	92[c]
4	Aluminum oxide	120	6	0
5	Sand	120	6	<1
6	FeS_2	120	6	0
7	$MgSO_4$	120	6	5
8	Silica	100	6	3
9	Silica	70	6	0
10	Silica	70	24	0

[a] Unless otherwise stated, reactions were performed in a 20 mL glass vial starting with 0.85 mmol of succinic acid, 400 mg of solid phase and 1 mL H_2O. [b] Determined from the ^1H NMR spectrum of the crude material. [c] Amount of anhydride **1** in the sublimate at the top of the vial; see below: "sublimate formation and analysis".

Example procedure (Table S1, Entry 2)

A 20 mL glass vial was charged with succinic acid (100 mg, 0.85 mmol), water (1 mL) and silica (400 mg). After manual stirring was performed, the vial was placed in a sand bath (120°C) for 6h. All the residues in the vial were taken up in anhydrous THF (5 mL). The silica was filtered off and washed with anhydrous THF (2x5 mL). The filtrate was concentrated under reduced pressure and the formation of succinic anhydride **1** was evidenced by NMR analysis. **^{1}H NMR** (400 MHz, acetone-d_6): δ_H 3.06 (s, 4H, CH_2).

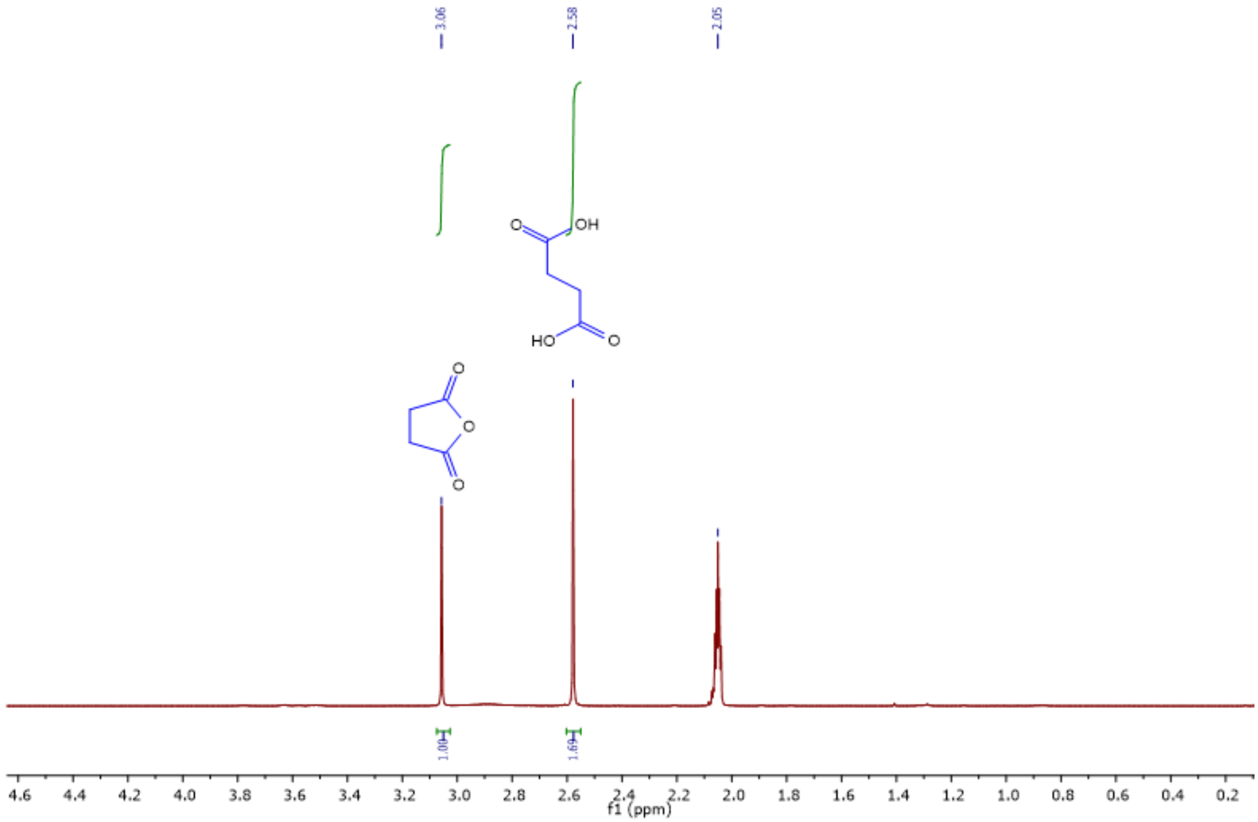

Fig. S1. Dehydration of succinic acid into anhydride 1: ^{1}H NMR spectrum (acetone-d_6) of organics recovered in the vial (Table S1, Entry 2).

Sublimate formation and analysis (Table S1, Entry 3)

While heating at 120°C (see above procedure), a sublimate built up at the neck of the vial. X-ray diffraction analysis of the crystals confirmed the formation of succinic anhydride **1**. The structure collected at 200K displays cell parameters that are logically inserted between those found at 150K and at 225K.[5] Data for the crystal structure have been deposited at the Cambridge Crystallographic Data Centre (reference no. CCDC 2331086). Besides, ^{1}H NMR analysis (acetone-d_6) of a sample showed a 92:8 ratio of succinic anhydride **1** and succinic acid.

4. Dehydration of succinic acid in the presence of morpholine

Table S2. Dehydration of succinic acid over silica in the presence of morpholine.[a]

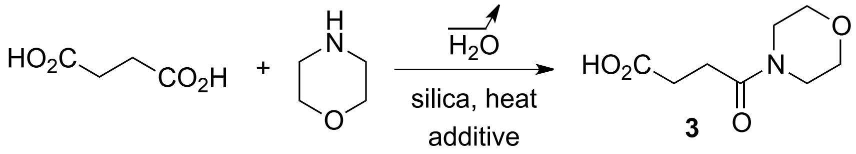

Entry	Morpholine x equiv.	Additive	T (°C)	Time (h)	Conversion (%) into 3[b]
1	1	–	100	3	80
2[c]	1	–	100	3	78
3[d]	1	–	100	70	56
4[c]	2	–	100	3	67
5	2	–	100	3	85
6[d]	3	–	100	3	10
7	1	$ZnCl_2$[e]	100	3	7
8[c]	1	$ZnCl_2$[e]	100	3	14
9	1	–	100	6	85
10	2	–	100	6	92
11	2	–	70	3	0
12	2	–	70	6	6
13	2	–	70	56	88
14	1	–	70	6	6
15	1	–	70	72	49

[a] Unless otherwise stated, reactions were performed in a 20 mL glass vial starting with 0.85 mmol of succinic acid, morpholine (x equiv.), 400 mg of silica and 1 mL H_2O; the pH of the solutions were ≈ 5-6 (x = 1) or 8-9 (x = 2). [b] Determined from the ^1H NMR spectrum of the reaction mixture (see example procedure). [c] Performed using 34 wt-% aqueous NaCl solution (1 mL) instead of water. [d] Performed without silica. [e] 1 equiv. of $ZnCl_2$.

Example procedure (Table S2, Entry 1)

A 20 mL glass vial was charged with succinic acid (100 mg, 0.85 mmol), a solution of morpholine (74 mg, 0.85 mmol) in water (1 mL) and silica (400 mg). After manual stirring was performed, the vial was placed in an oil bath (100°C) for 3h. The residues were washed with water (2x5 mL) on a filter funnel. A sample (0.45 μL) of the filtrate ("reaction mixture") was transferred into a NMR tube together with D_2O (0.05 μL) and NMR experiments (with water suppression in the case of ^{1}H NMR) were performed. The remaining filtrate was concentrated under reduced pressure (< 10 mbar, 40 °C). An analytical sample of 4-morpholino-4-oxobutanoic acid **3** (colorless oil) was obtained by column chromatography purification of the crude product (POLYGOPREP 60-50 C_{18}; eluent DCM/MeOH – 9.8:0.2). **^{1}H NMR** (400 MHz, acetone-*d_6*): δ_H 3.65-3.57 (m, 4H, C*H_2*-O), 3.54-3.50 (m, 4H, C*H_2*-N), 2.65-2.61 (m, 2H, C*H_2*-CON), 2.59-2.55 (m, 2H, C*H_2*-CO_2). **^{13}C NMR** (100 MHz, acetone-*d_6*): δ_C 174.2 (*C*O_2H), 170.6 (*C*ON), 67.0 (*C*H_2-O), 66.9 (*C*H_2-O), 46.2 (*C*H_2-N), 42.5 (*C*H_2-N), 29.3 (*C*H_2-CO_2), 28.0 (*C*H_2-CON). **HRMS** (ESI): *m/z* $[M+H]^+$ Calcd for $C_8H_{14}NO_4$ 188.0917; Found 188.0920.

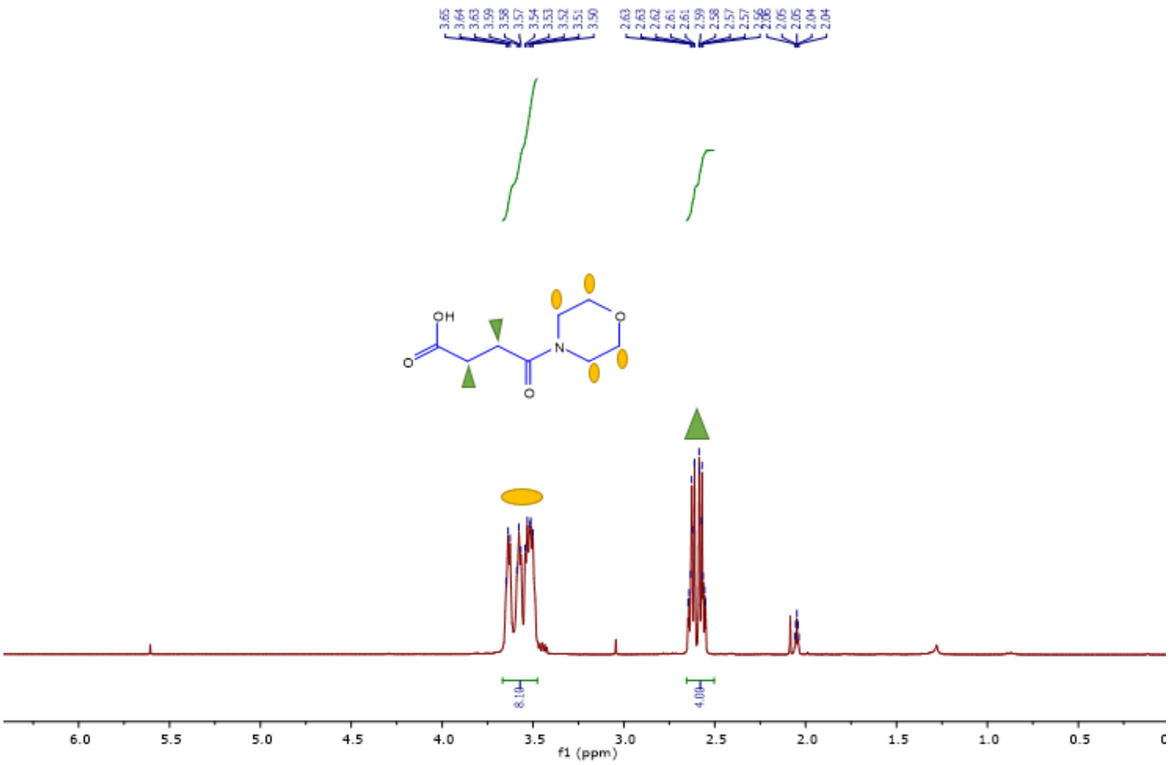

Fig. S2. ^{1}H NMR spectrum (acetone-*d_6*) of purified 4-morpholino-4-oxobutanoic acid **3**.

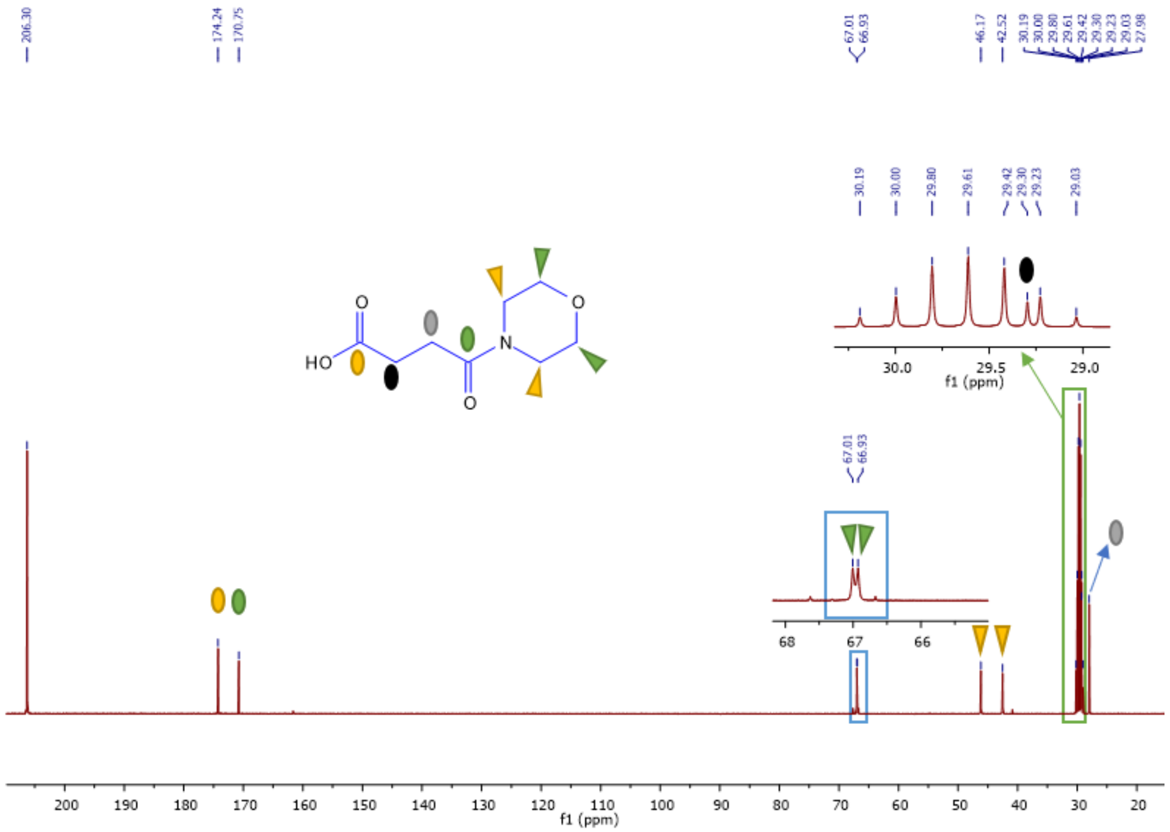

Fig. S3. ^{13}C NMR spectrum (acetone-d_6) of purified 4-morpholino-4-oxobutanoic acid **3**.

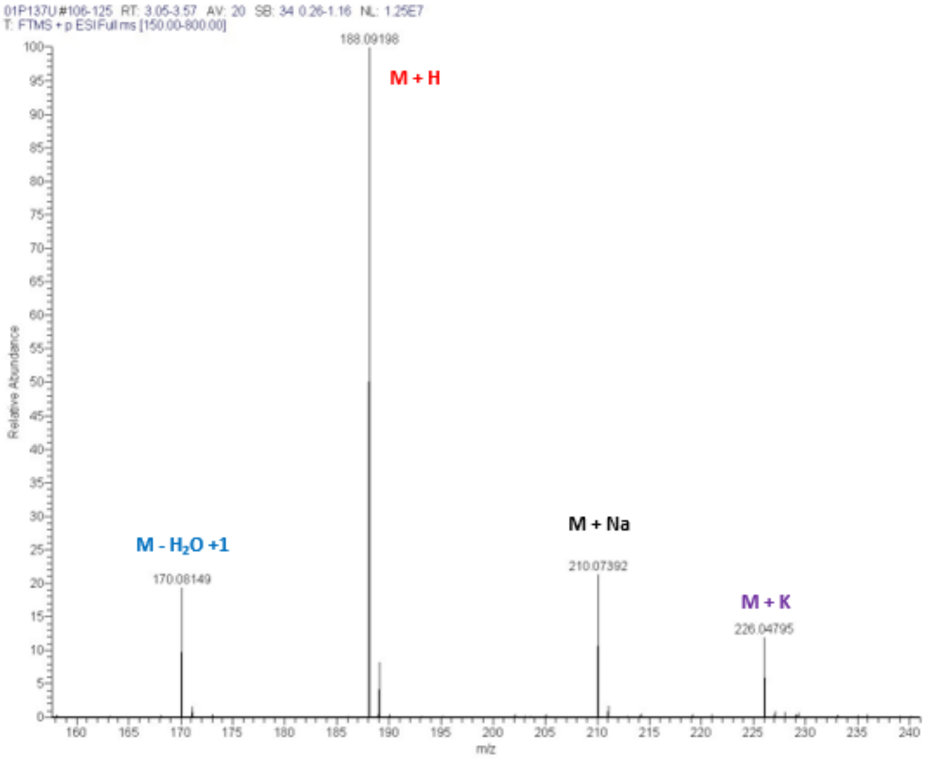

Fig. S4. HRMS spectrum of purified 4-morpholino-4-oxobutanoic acid **3**.

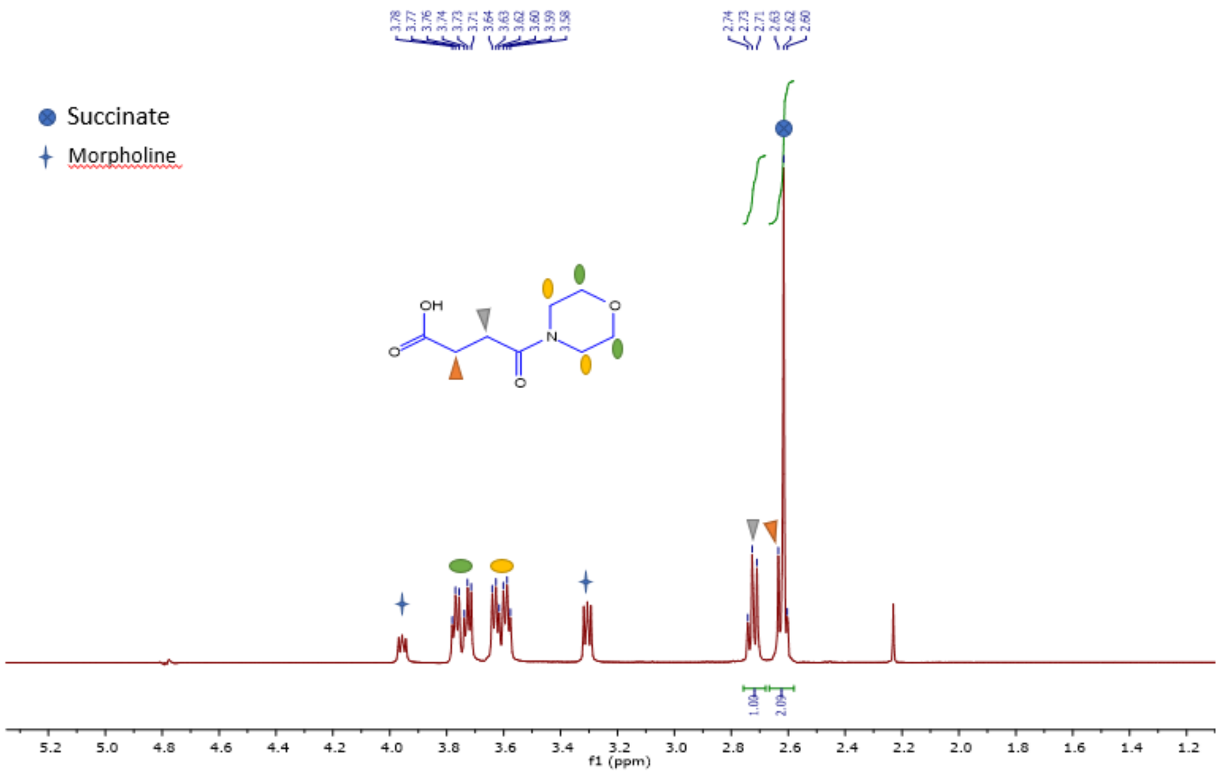

Fig. S5. ^{1}H NMR spectrum (D_2O) of the reaction mixture for the dehydration of succinic acid over silica in the presence of 2 equivalents morpholine (Table S2, Entry 4).

5. Competitive reactions between succinic acid and glutaric acid

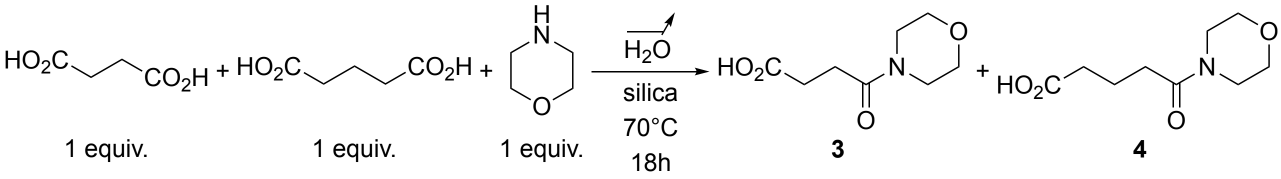

succinic acid conversion into **3**: 55%

glutaric acid conversion into **4**: 2%

The conversions were estimated as follows (Fig. S6):

- for succinic acid: by comparison of the ^{1}H NMR signals of the CH_2CO in the product (yellow and green ovals) and in the starting material (blue oval);

- for glutaric acid: first, the integral value for 8H in **4** (0.39) was deduced from the overlayed signals of the morpholine moiety (CH_2O and CH_2N in **3** and **4**; 8.39) by substraction of the estimated value for 8H in **3** (8.00; from CH_2CO-N, green oval). Second, the obtained value reduced to 2H (respectively 4H) in **4** was compared to the integral value of overlayed CH_2 signals of glutaric acid and **4**, grey vs. green triangles (resp. orange and yellow vs. blue triangles).

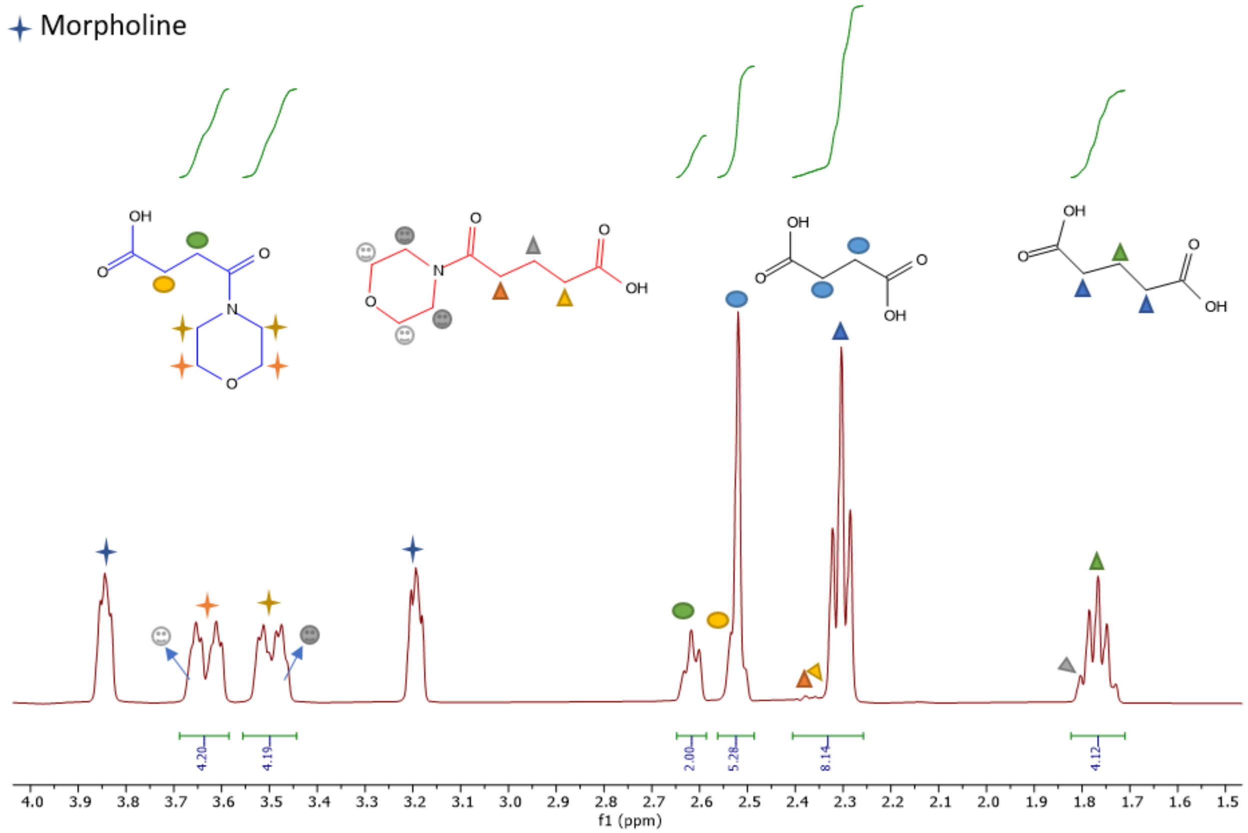

Fig. S6. Competitive dehydrations of succinic and glutaric acid over silica in the presence of morpholine (1 equiv.); ^{1}H NMR spectrum (D_2O) of the reaction mixture.

6. Dehydration of citric acid in the presence of morpholine

Table S3. Dehydration of citric acid over silica in the presence of morpholine.[a]

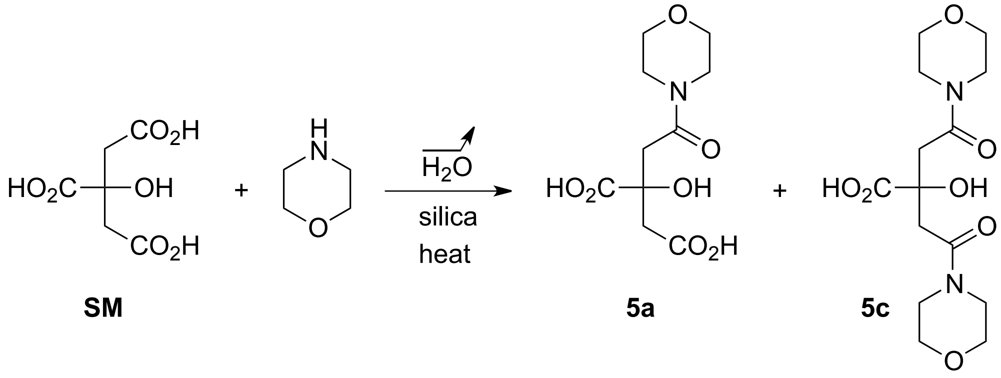

Entry	Morpholine x equiv.	Additive	T (°C)	Time (h)	Ratio (%) [b]		
					SM	5a	5c
1	2	–	70	3	100	0	0
2	2	–	70	6	82	18	0
3	1	–	70	6	92	8	0
4	1	–	70	72	87	13	0
5	1	–	100	3	73	27	0
6[c]	1	–	100	3	79	21	0
7[c]	2	–	100	3	55	38	7
8	2	–	100	3	86	14	0
9	1	$ZnCl_2$ [d]	100	3	99	trace	0
10[c]	1	$ZnCl_2$ [d]	100	3	90	10	0
11	1	–	100	6	61	30	9
12	2	–	100	6	51	36	13
13	1	–	120	3	22	54	24
14	1	–	120	6	25	49	26
15	2	–	120	3	0	30	70
16	2	–	120	6	7	45	48

[a] Unless otherwise stated, reactions were performed in a 20 mL glass vial starting with 0.52 mmol of citric acid, morpholine (x equiv.), 400 mg of silica and 1 mL H_2O; the pH of the solutions were ≈ 1-2 (x = 1) or 5-6 (x = 2). [b] Ratio between citric acid (**SM**), monoamide (**5a**) and symmetrical diamide (**5c**), determined from the ^{1}H NMR spectrum of the reaction mixture (see example procedures). [c] Performed using 34 wt-% aqueous NaCl solution (1 mL) instead of water. [d] 1 equiv. of $ZnCl_2$.

Example procedure (Table S3, Entry 5)

A 20 mL glass vial was charged with citric acid (100 mg, 0.52 mmol), a solution of morpholine (45 mg, 0.52 mmol) in water (1 mL) and silica (400 mg). After manual stirring was performed, the vial was placed in an oil bath (100°C) for 3h. The residues were washed with water (2x5 mL) on a filter funnel. A sample (0.45 μL) of the filtrate ("reaction mixture") was transferred into a NMR tube together with D_2O (0.05 μL) and NMR experiments (with water suppression in the case of ^{1}H NMR) were performed. The remaining filtrate was concentrated under reduced pressure (< 10 mbar, 40 °C). An analytical sample of 2-hydroxy-2-(2-morpholino-2-oxoethyl)succinic acid **5a** (colorless oil) was obtained by column chromatography purification of the crude product (POLYGOPREP 60-50 C_{18}; eluent DCM/MeOH – 9.7:0.3). **^{1}H NMR** (400 MHz, D_2O): δ_H 3.69-3.64 (m, 4H, CH_2-O), 3.55-3.50 (m, 4H, CH_2-N), 3.02-2.91 (m, 2H), 2.94 (d, J = 16 Hz, 1H), 2.76 (d, J = 16 Hz, 1H). **^{13}C NMR** (100 MHz, D_2O): δ_C 177.4, 173.8, 169.8, 73.9, 66.2, 46.4, 43.5, 42.1, 40.9. **HRMS** (ESI): m/z $[M-H]^-$ Calcd for $C_{10}H_{14}NO_7$ 260.0776; Found 260.0772.

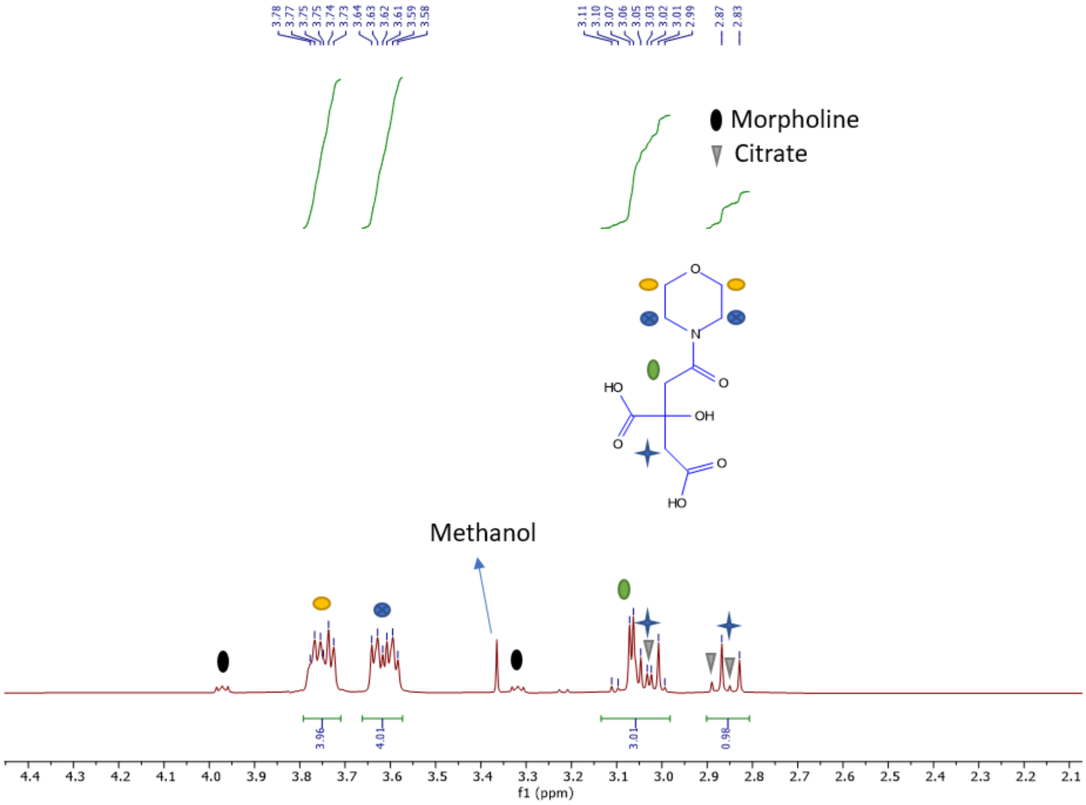

Fig. S7. ^{1}H NMR spectrum (D_2O) of purified 2-hydroxy-2-(2-morpholino-2-oxoethyl)succinic acid **5a**.

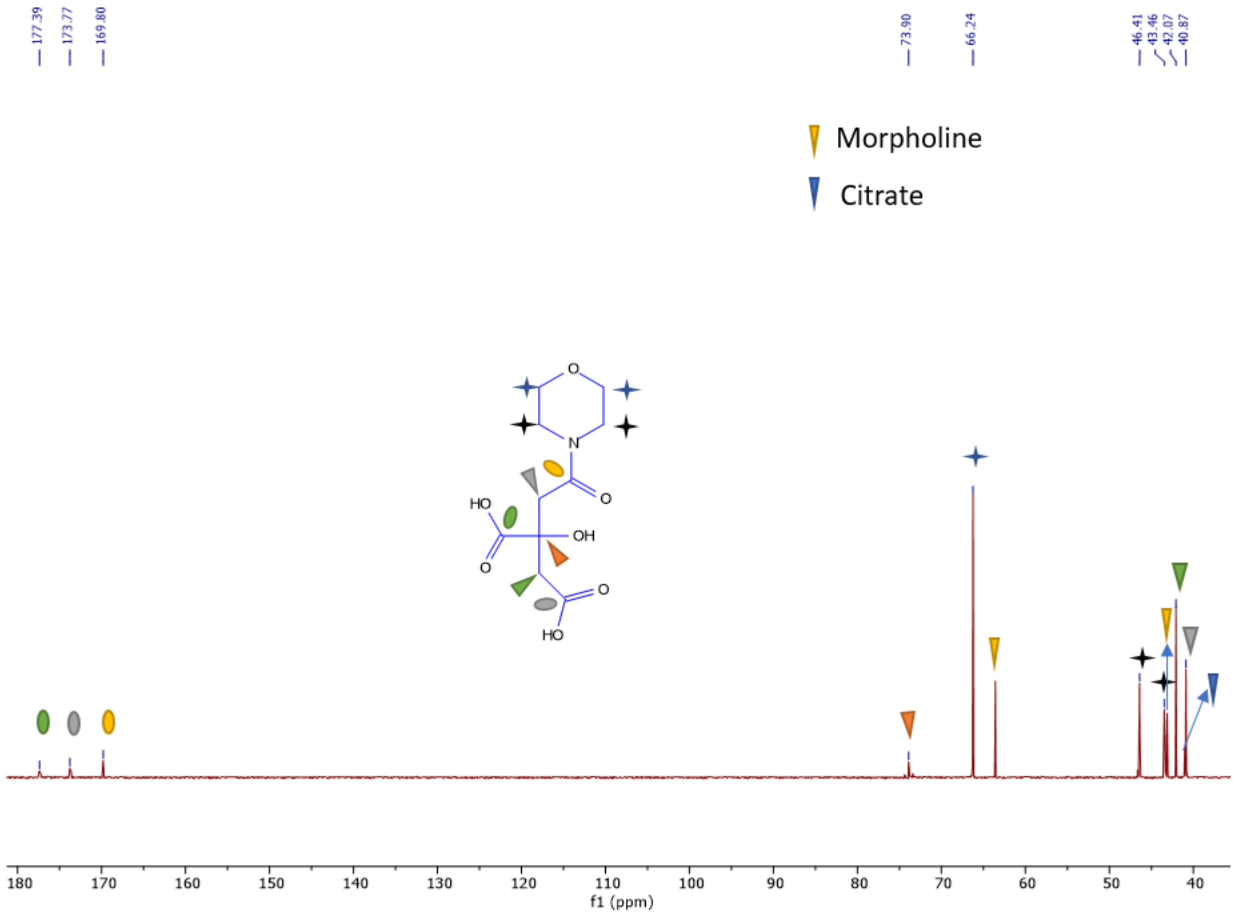

Fig. S8. ^{13}C NMR spectrum (D_2O) of purified 2-hydroxy-2-(2-morpholino-2-oxoethyl)succinic acid **5a**.

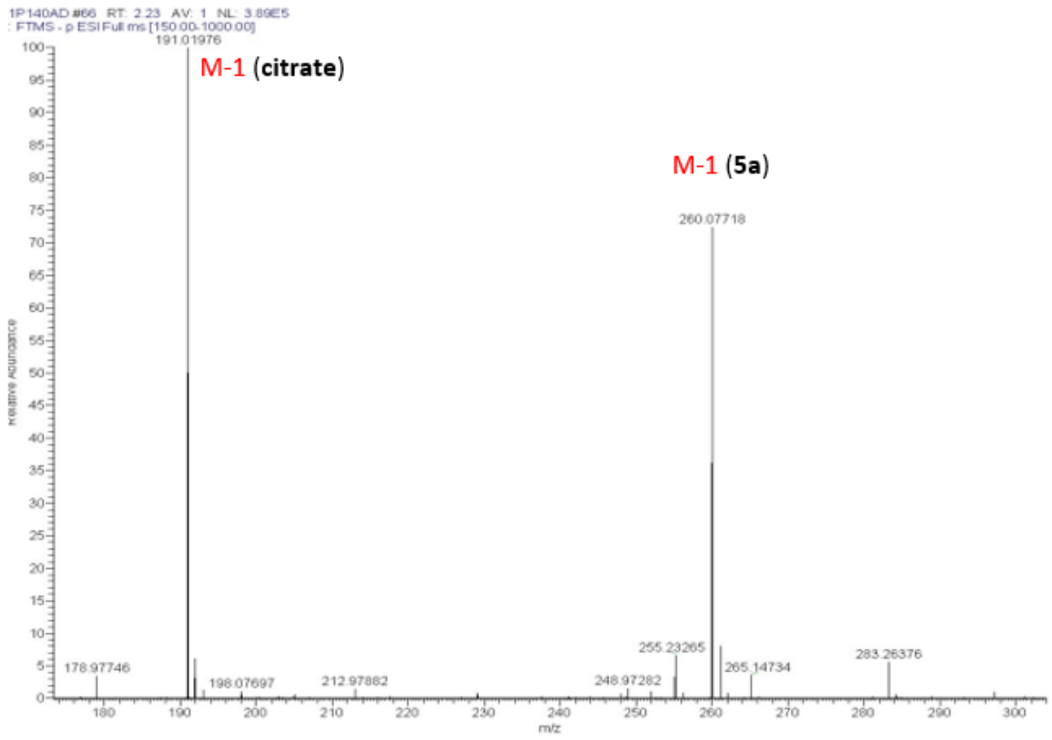

Fig. S9. HRMS spectrum of the reaction mixture for the dehydration of citric acid over silica in the presence of 1 equivalent morpholine (Table S3, Entry 5).

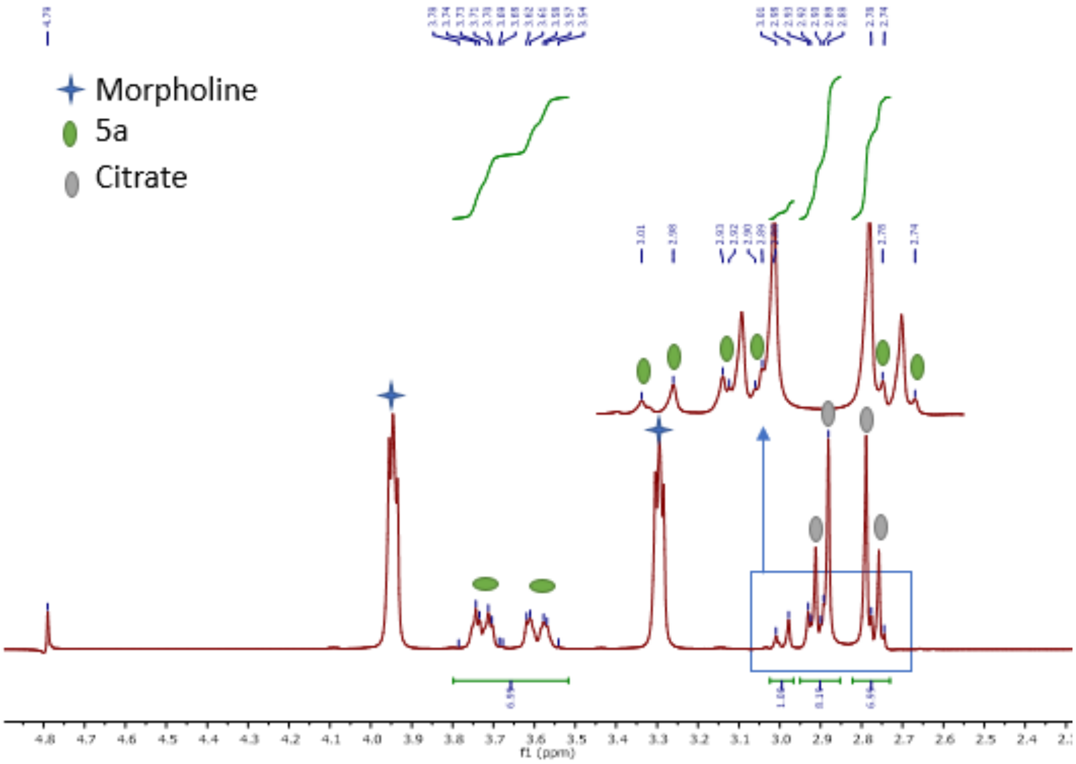

Fig. S10. ^{1}H NMR spectrum (D_2O) of the reaction mixture for the dehydration of citric acid over silica in the presence of 1 equivalent morpholine (Table S3, Entry 5).

Example procedure (Table S3, Entry 12)

A 20 mL glass vial was charged with citric acid (100 mg, 0.52 mmol), a solution of morpholine (90 mg, 1.04 mmol) in water (1 mL) and silica (400 mg). After manual stirring was performed, the vial was placed in an oil bath (100°C) for 6h. The residues were washed with water (2x5 mL) on a filter funnel. A sample (0.45 μL) of the filtrate ("reaction mixture") was transferred into a NMR tube together with D_2O (0.05 μL) and NMR experiments (with water suppression in the case of ^1H NMR) were performed. The remaining filtrate was concentrated under reduced pressure (< 10 mbar, 40 °C). An analytical sample of 2-hydroxy-4-morpholino-2-(2-morpholino-2-oxoethyl)-4-oxobutanoic acid **5c** (colorless oil) was obtained by column chromatography purification of the crude product (POLYGOPREP 60-50 C_{18}; eluent DCM/MeOH – 9.5:0.5). **^{1}H NMR** (500 MHz, D_2O): δ_H 3.78-3.73 (m, 8H), 3.64-3.59 (m, 8H), 3.06 (d, *J* = 16 Hz, 2H), 2.98 (d, *J* = 16 Hz, 2H). **HRMS** (ESI): *m/z* $[M-H]^-$ Calcd for $C_{14}H_{21}N_2O_7$ 329.1354; Found 329.1349.

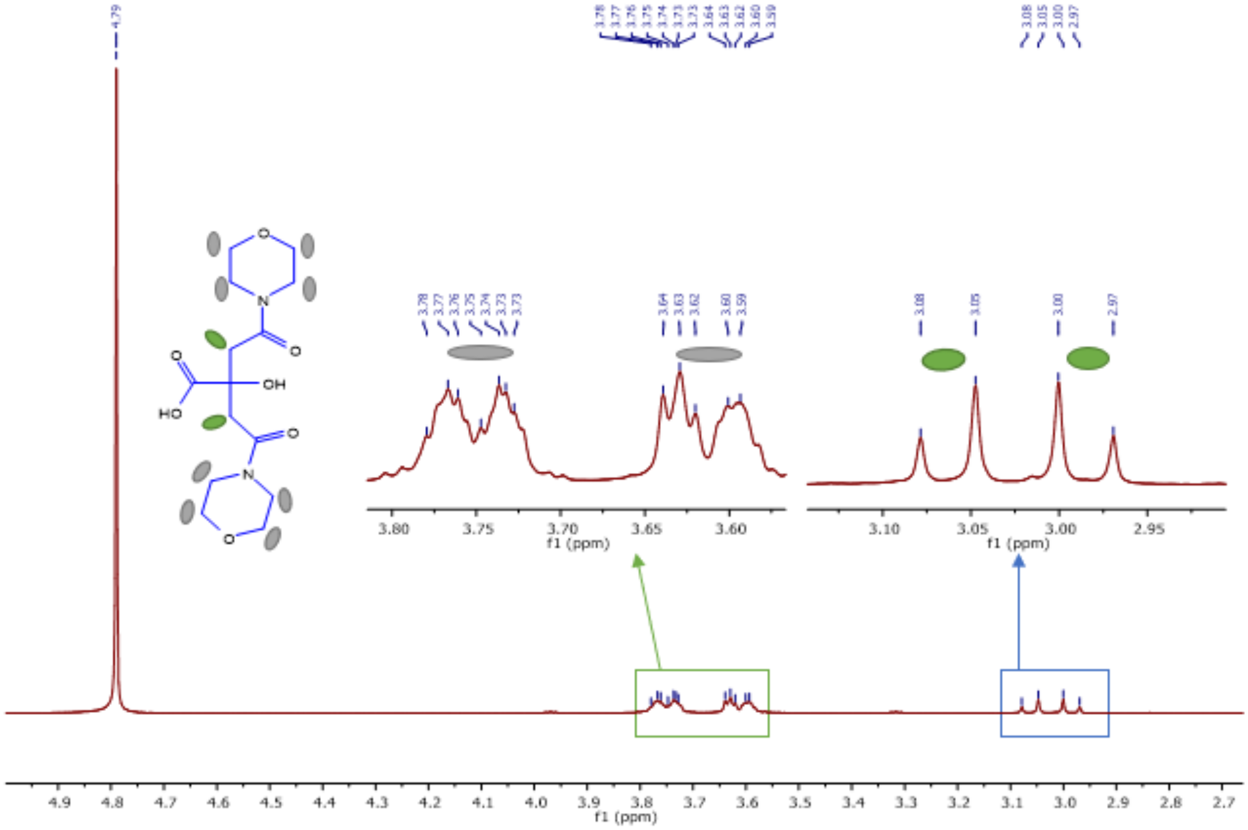

Fig. S11. ^{1}H NMR spectrum (D_2O) of purified 2-hydroxy-4-morpholino-2-(2-morpholino-2-oxoethyl)-4-oxobutanoic acid **5c**.

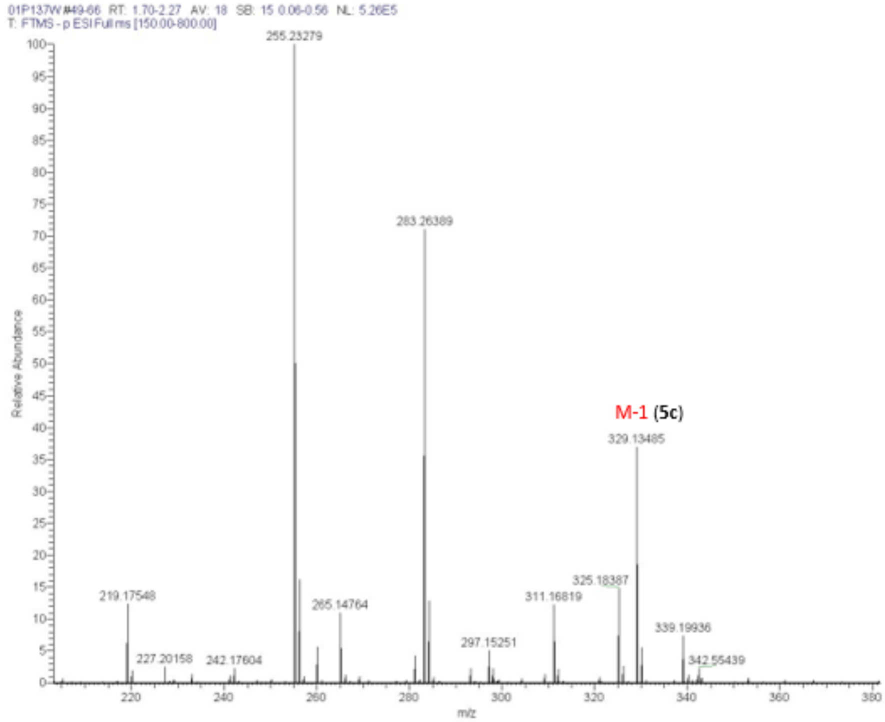

Fig. S12. HRMS spectrum of the reaction mixture for the dehydration of citric acid over silica in the presence of 2 equivalents morpholine (Table S3, Entry 12). Peaks at *m/z* 255 and 283 correspond to contaminants from the MS experiment.

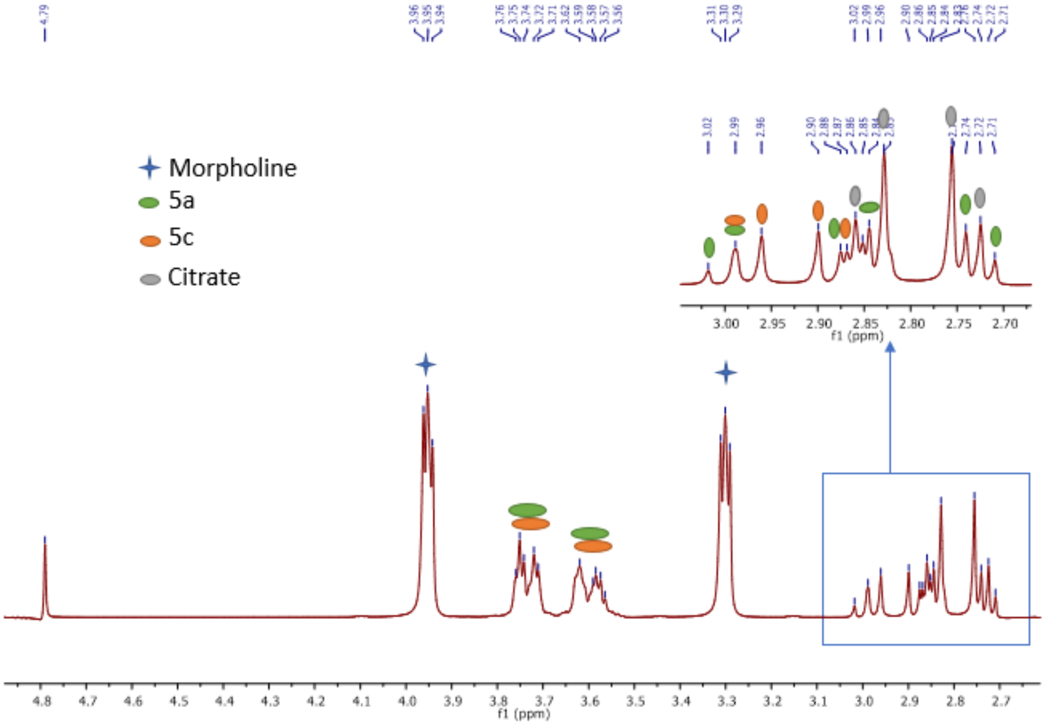

Fig. S13. ^1H NMR spectrum (D_2O) of the reaction mixture for the dehydration of citric acid over silica in the presence of 2 equivalents morpholine (Table S3, Entry 12).

7. Dehydration of succinic acid in the presence of Coenzyme M

Reference NMR spectra

Succinic anhydride (10 mg, 0.1 mmol) and sodium 2-mercaptoethanesulfonate (CoM, 1 equiv.) were mixed in D_2O (0.5 mL) in an NMR tube. The ^1H and ^{13}C NMR spectra (Fig. S14-S15) were recorded immediately, showing a mixture of succinic acid, CoM and thioester **6** (sodium 2-((3-carboxypropanoyl)thio)ethanesulfonate).

Characterization data for **6**: **^{1}H NMR** (500 MHz, D_2O): δ_H 3.27 (dd, *J* = 8.5, 5.0 Hz, 2H), 3.16 (dd, *J* = 8.5, 5.0 Hz, 2H), 3.00 (t, *J* = 6.9 Hz, 2H), 2.76 (t, *J* = 6.9 Hz, 2H). **^{13}C NMR** (125 MHz, D_2O): δ_C 201.7, 176.6, 50.3, 38.0, 29.0, 23.4.

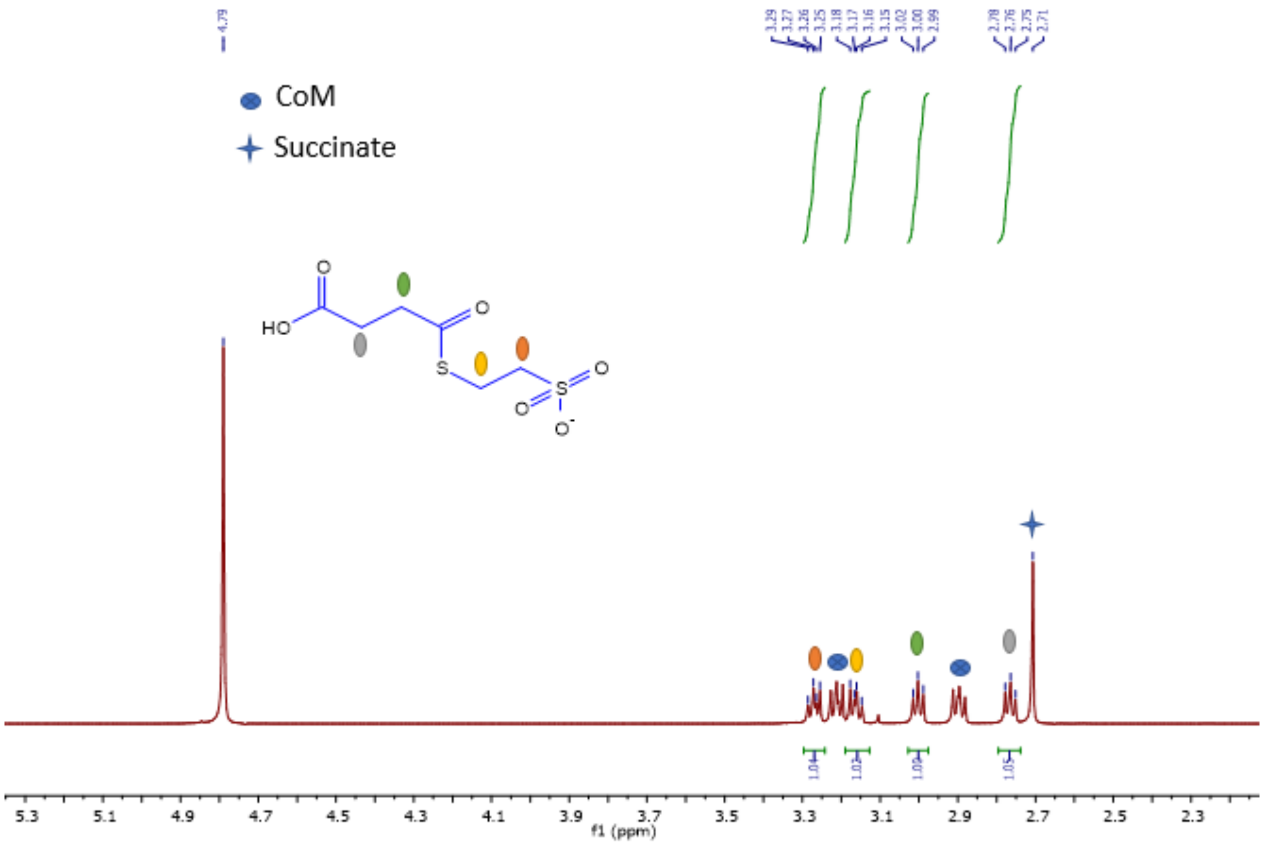

Fig. S14. ^{1}H NMR spectrum obtained from the reaction of succinic anhydride with CoM in D_2O.

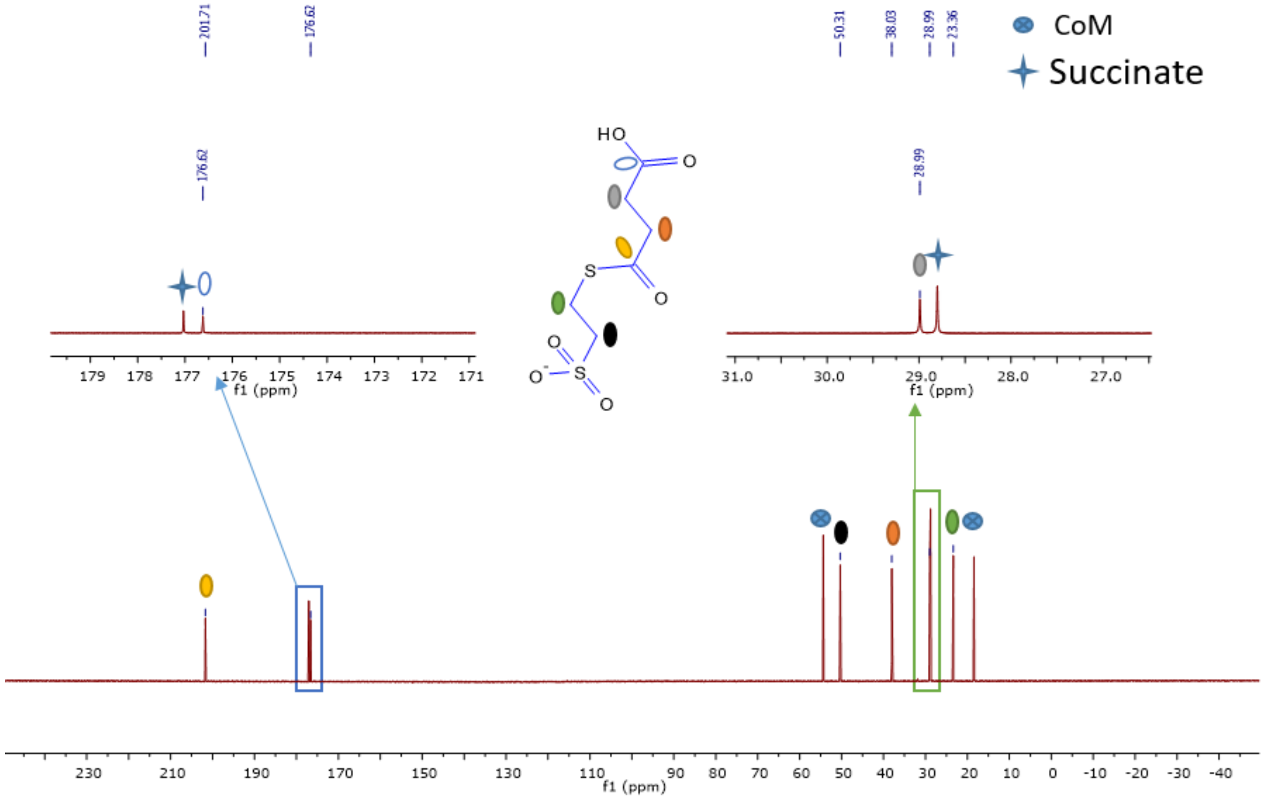

Fig. S15. ^{13}C NMR spectrum obtained from the reaction of succinic anhydride with CoM in D_2O.

Table S4. Dehydration of succinic acid over silica in the presence of CoM.[a]

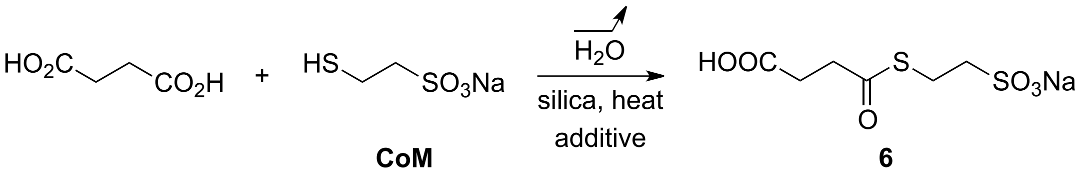

Entry	CoM x equiv.	Additive (y equiv.) [b]	Wet/dry cycles	T (°C)	Time (h)	Conversion (%) into 6 [c]
1	1	–	1	70	0.25	0
2	1	–	1	70	16	5
3	1	–	1	70	48	6
4	3	–	1	70	16	9
5	0.5	–	1	70	16	5
6	1	–	2	70	16	5
7	1	–	5	70	48	6
8	1	$CaCl_2$ (1)	1	70	16	0
9	1	$CuCl_2$ (1)	1	70	16	0
10	1	$MgCl_2$ (1)	1	70	16	0
11	1	$ZnCl_2$ (1)	1	70	16	5
12	1	$BaCl_2$ (1)	1	70	16	5
13	1	–	1	100	3	9
14	1	–	1	100	16	21
15	1	–	3	100	3x3	24
16	2	–	1	100	3	27
17	3	–	1	100	3	26
18	2	–	1	100	16	27
19	1 + 2x0.5 [d]	–	3	100	3x3	22
20 [e]	1	–	1	100	3	21

21 [e]	1	–	1	100	16	22
22 [e]	1	–	3	100	3x3	23
23 [e]	2	–	1	100	3	15
24 [e]	3	–	1	100	3	10
25	6x0.5 [f]	–	6	100	6x3	36
26	1	$ZnCl_2$ (1)	1	100	3	14
27	1	$ZnCl_2$ (1)	1	100	16	14
28	1	$CaCl_2$ (1)	1	100	3	2
29	1	$CaCl_2$ (1)	1	100	16	11
30	1	$MgCl_2$ (1)	1	100	3	0
31	1	$MgCl_2$ (1)	1	100	16	Trace
32	1	$KHCO_3$ (1)	1	100	3	11
33	1	K_2CO_3 (0.5)	1	100	3	11
34	1	$NaHCO_3$ (1)	1	100	3	10
35	1	Na_2CO_3 (0.5)	1	100	3	15
36	1	Na_2CO_3 (0.5)	1	100	16	16
37	1	Na_2CO_3 (0.5)	3	100	3x3	15
38	1	Cs_2CO_3 (0.5)	1	100	3	10
39	1	Na_3PO_4 (0.33)	1	100	3	17
40	1	Na_3PO_4 (0.33)	1	100	16	20
41	1	Na_3PO_4 (0.33)	3	100	3x3	21

[a] Unless otherwise stated, reactions were performed in a 20 mL glass vial starting with 0.85 mmol of succinic acid, CoM (x equiv.), 400 mg of silica and 1 mL H_2O; the pH of the solutions were ≈ 1-2 for entries 1-31 and ≈ 4-6 for entries 32-41. [b] The additive was added before silica in the procedure. [c] Determined from the ^{1}H NMR spectrum of the reaction mixture (see example procedure). [d] The reaction was started with 1 equiv. CoM. After every 3h, more water (1 mL) and CoM (0.5 equiv.) were added. [e] Performed using 34 wt-% aqueous NaCl solution (1 mL) instead of water. [f] The reaction was started with 0.5 equiv. CoM. After every 3h, more water (1 mL) and CoM (0.5 equiv.) were added.

Example procedure (Table S4, Entry 21)

A 20 mL glass vial was charged with succinic acid (100 mg, 0.85 mmol), 34 wt-% aqueous NaCl solution (1 mL), sodium 2-mercaptoethanesulfonate (CoM, 140 mg, 0.85 mmol) and silica (400 mg). After manual stirring was performed, the vial was placed in an oil bath (100°C) for 16h. The residues were washed with water (2x5 mL) on a filter funnel. A sample (0.45 μL) of the filtrate ("reaction mixture") was transferred into a NMR tube together with D_2O (0.05 μL) and NMR experiments (with water suppression in the case of ^{1}H NMR) were performed. The formation of thioester **6** (sodium 2-((3-carboxypropanoyl)thio)ethanesulfonate) was evidenced by the NMR analyses (Fig. S16-S17) and by HRMS analysis of the filtrate (Fig. S18). **HRMS** (ESI): *m/z* $[M-Na]^-$ Calcd for $C_6H_9O_6S_2$ 240.9846; Found 240.9842.

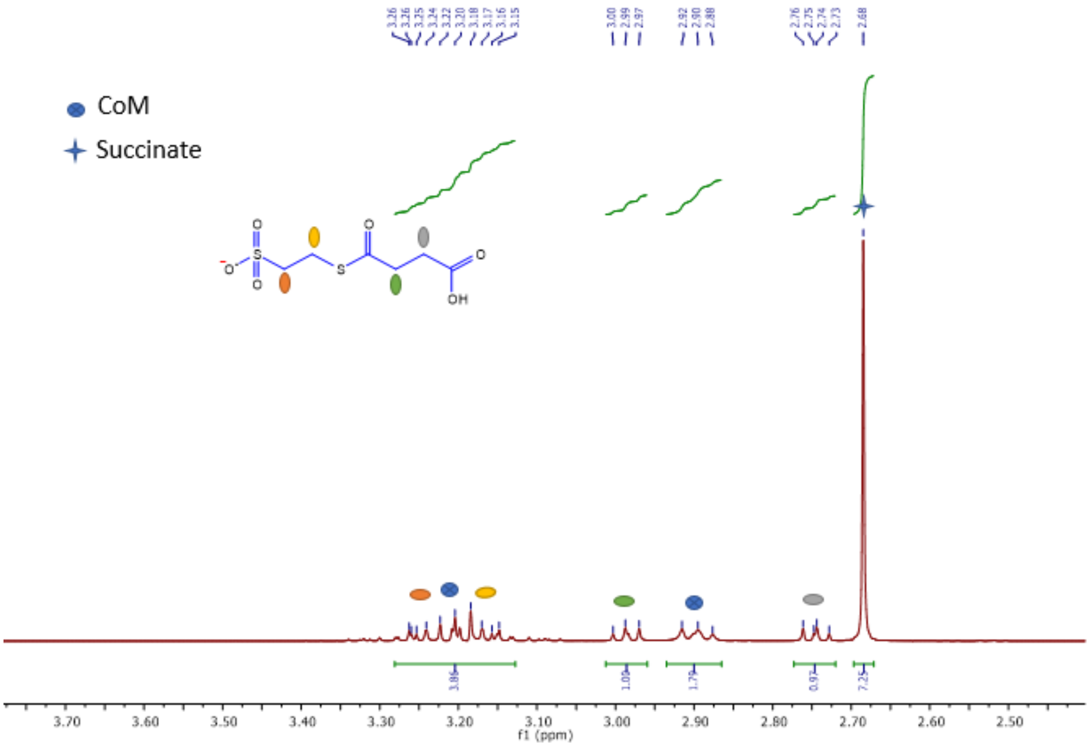

Fig. S16. ^{1}H NMR spectrum (D_2O) of the reaction mixture for the dehydration of succinic acid over silica in the presence of 1 equivalent CoM (Table S4, Entry 21).

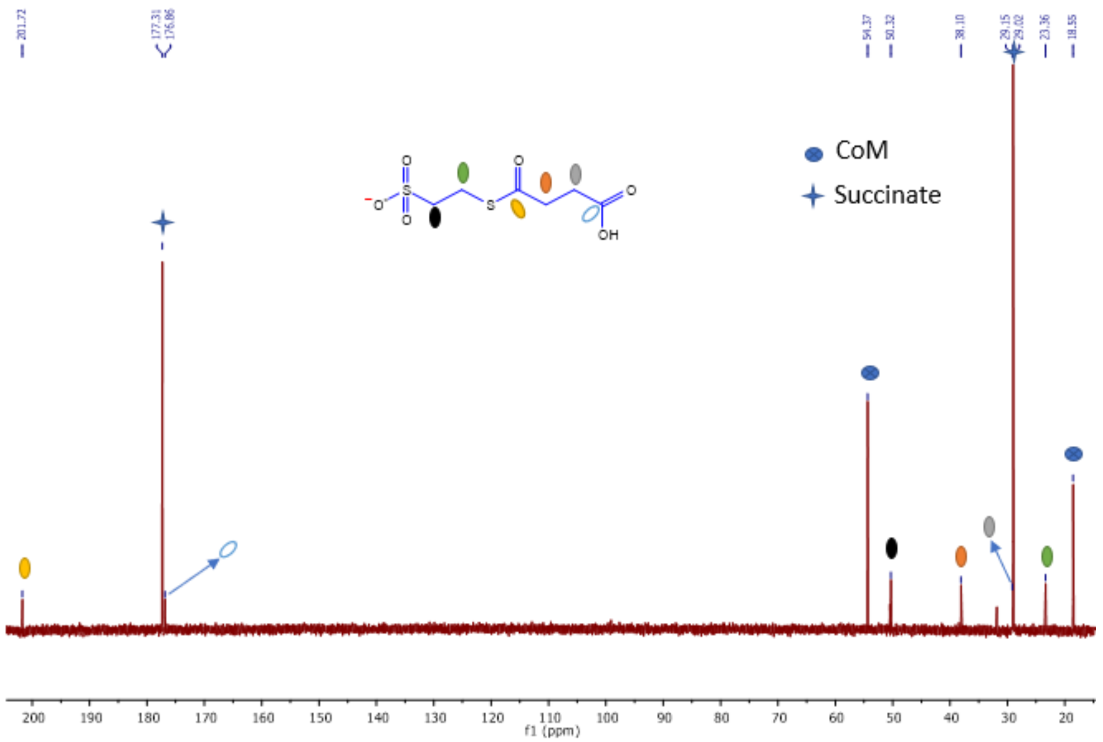

Fig. S17. ^{13}C NMR spectrum (D_2O) of the reaction mixture for the dehydration of succinic acid over silica in the presence of 1 equivalent CoM (Table S4, Entry 21).

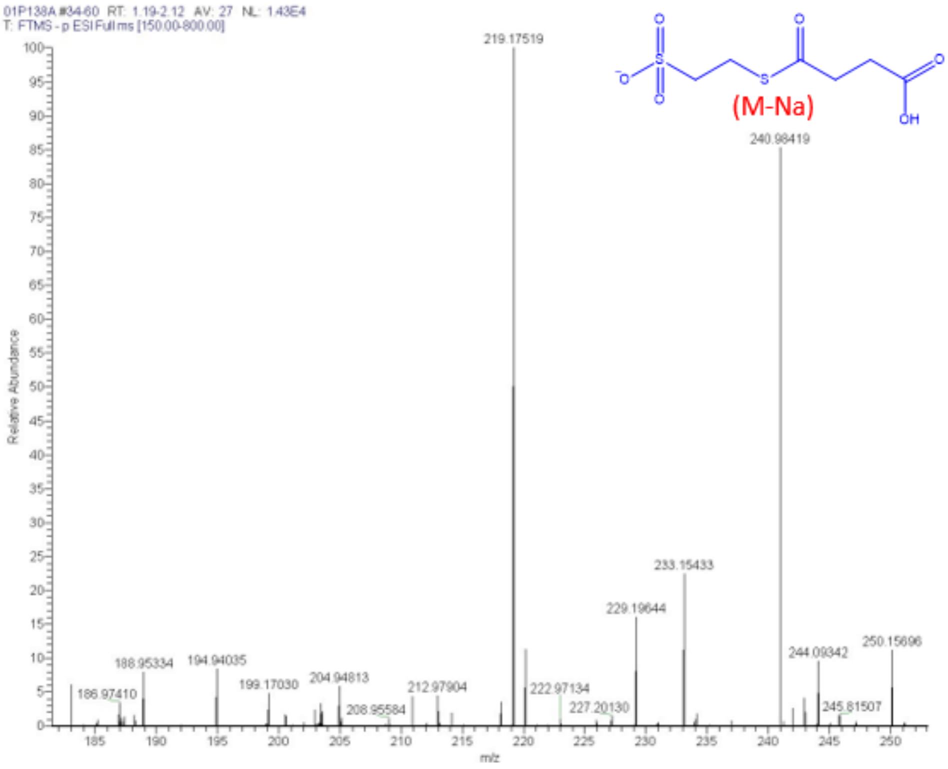

Fig. S18. HRMS spectrum of the reaction mixture for the dehydration of succinic acid over silica in the presence of 1 equivalent CoM (Table S4, Entry 3).

8. Dehydration of citric acid in the presence of Coenzyme M

Table S5. Dehydration of citric acid over silica in the presence of CoM.[a]

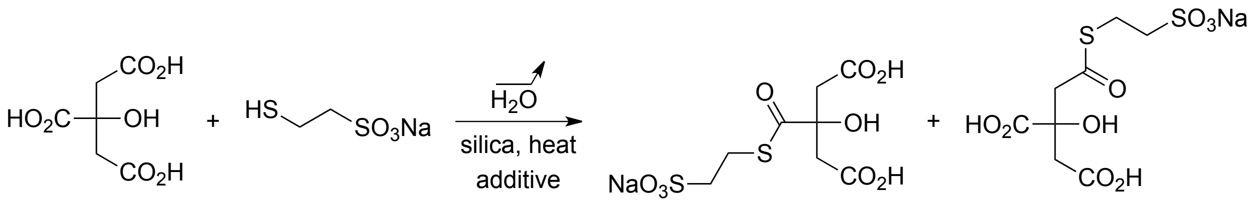

CoM 7 8

Entry	Additive (x equiv.)[b]	T (°C)	Time (h)	Conversion (%)[c] into:	
				7	8
1	–	70	16	0	0
2	–	70	72	9	0
3	$CaCl_2$ (1)	70	72	0	0
4	$MgCl_2$ (1)	70	72	0	0
5	$Zn(OH)_2$ (1)	70	72	0	0
6[d]	$FeCl_2$ (1)	70	72	0	0
7[d]	$FeCl_3$ (1)	70	72	0	0
8	$BaCl_2$ (1)	70	72	0	<1
9	$ZnCl_2$ (1)	70	72	0	7
10	$ZnCl_2$ (3)	70	72	0	6
11	$ZnCl_2$ (0.5)	70	72	8	5
12[e]	$ZnCl_2$ (1)	70	72	0	7
13	$ZnCl_2$ (1)	70	16	<1	<1
14	$ZnCl_2$ (1)	70	4.5	0	0
15	$CaCl_2$ (1)	100	3	0	0
16	$MgCl_2$ (1)	100	3	0	0
17	$ZnCl_2$ (1)	100	3	0	16
18	$ZnCl_2$ (1)	100	16	0	27

19	$ZnCl_2$ (1)	100	40	0	24
20 [f]	$ZnCl_2$ (1)	100	3x3	0	11
21 [f]	$ZnCl_2$ (1)	100	6x3	0	19
22	$ZnCl_2$ (2)	100	3	0	5
23	$ZnCl_2$ (2)	100	16	0	11
24 [e]	$ZnCl_2$ (1)	100	3	0	22
25 [e]	$ZnCl_2$ (1)	100	16	0	28
26	–	100	3	4	0
27	–	100	16	7	7

[a] Unless otherwise stated, reactions were performed in a 20 mL glass vial starting with 0.52 mmol of citric acid, CoM (1 equiv.), 400 mg of silica and 1 mL H_2O; the pH of the solutions were ≈ 1-2. [b] The additive was added before silica in the procedure. [c] Estimated from the ^{13}C NMR spectrum of the reaction mixture (see example procedures). [d] A treatment with Chelex 100 resin was applied before NMR analysis. [e] Performed using 34 wt-% aqueous NaCl solution (1 mL) instead of water. [f] The reaction was started with 0.5 equiv. CoM. After every 3h, more water (1 mL) and CoM (0.5 equiv.) were added.

Example procedure without additive (Table S5, Entry 2)

A 20 mL glass vial was charged with citric acid (100 mg, 0.52 mmol), water (1 mL), sodium 2-mercaptoethanesulfonate (CoM, 85 mg, 0.52 mmol) and silica (400 mg). After manual stirring was performed, the vial was placed in an oil bath (70°C) for 72h. The residues were washed with water (2x5 mL) on a filter funnel. A sample (0.45 μL) of the filtrate ("reaction mixture") was transferred into a NMR tube together with D_2O (0.05 μL) and NMR experiments were performed. The formation of thioester **7** (sodium 2-((3-carboxy-2-(carboxymethyl)-2-hydroxypropanoyl)thio)ethanesulfonate) was evidenced by the ^{13}C NMR analysis (Fig. S19) and by HRMS analysis of the filtrate (Fig. S20). **^{13}C NMR** (100 MHz, D_2O): δ_c 206.6 (*C*OS), 172.9 (*C*OO), 78.6, 49.9, 43.0, 23.1. **HRMS** (ESI): *m/z* $[M-Na]^-$ Calcd for $C_8H_{11}O_9S_2$ 314.9850; Found 314.9845.

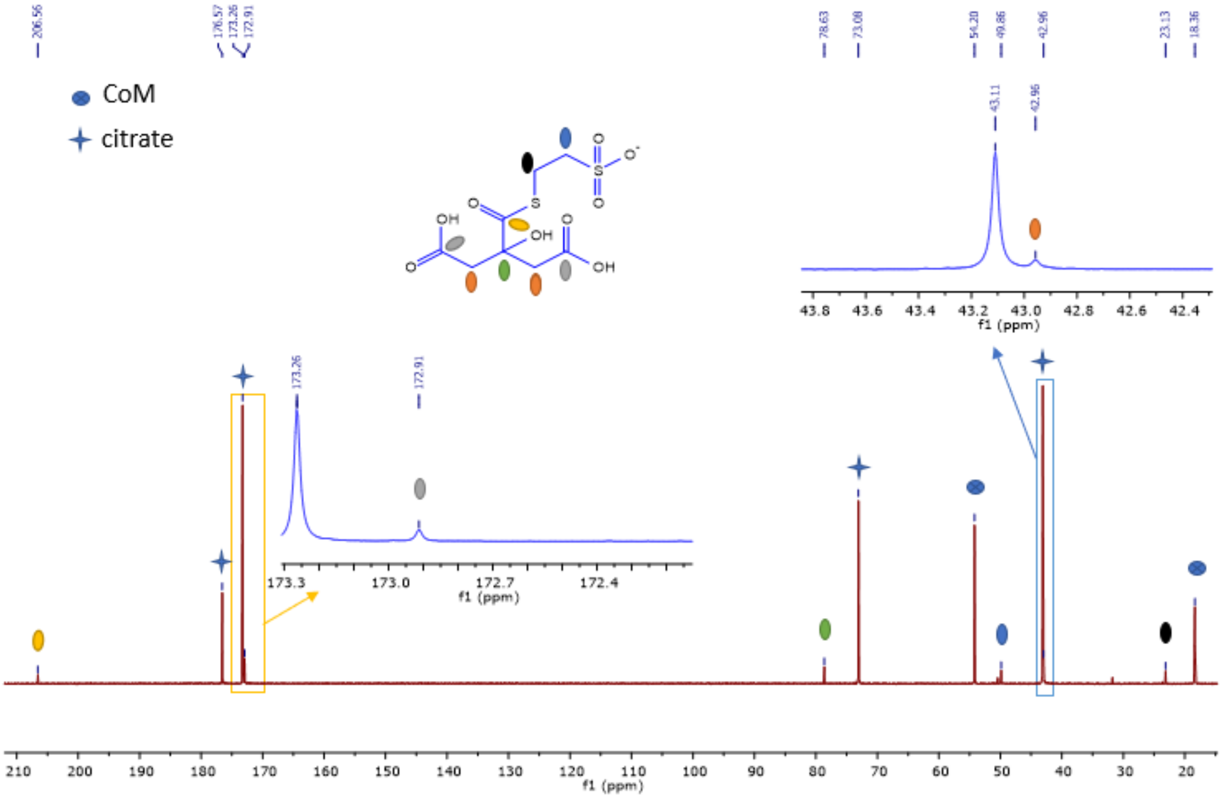

Fig. S19. ^{13}C NMR spectrum (D_2O) of the reaction mixture for the dehydration of citric acid over silica in the presence of 1 equivalent CoM (Table S5, Entry 2).

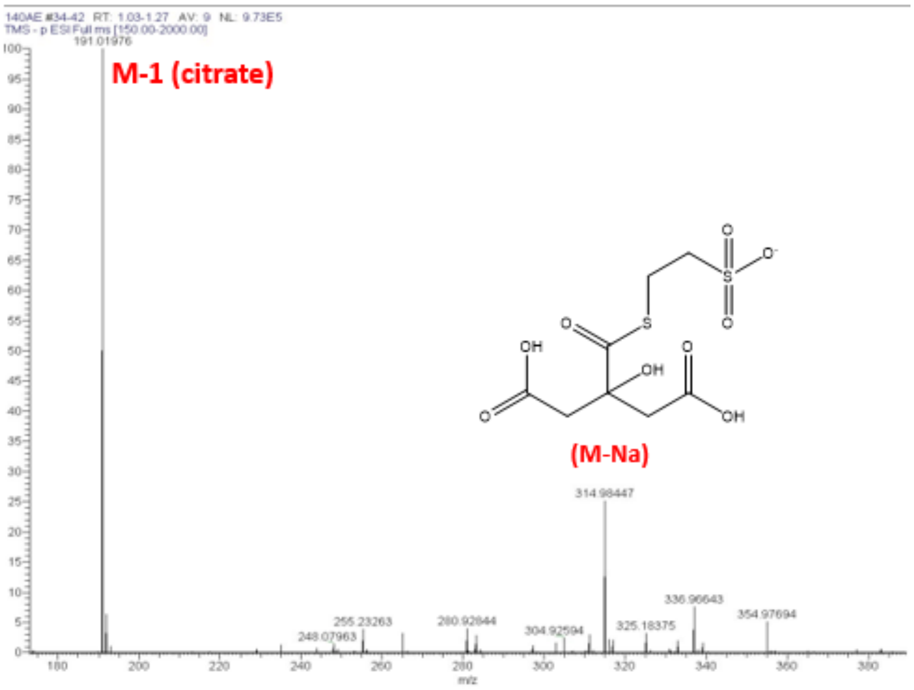

Fig. S20. HRMS spectrum of the reaction mixture for the dehydration of citric acid over silica in the presence of 1 equivalent CoM (Table S5, Entry 2).

Example procedure with additive (Table S5, Entry 18)

A 20 mL glass vial was charged with citric acid (100 mg, 0.52 mmol), water (1 mL), sodium 2-mercaptoethanesulfonate (CoM, 85 mg, 0.52 mmol), $ZnCl_2$ (71 mg, 0.52 mmol) and silica (400 mg). After manual stirring was performed, the vial was placed in an oil bath (100 °C) for 16h. The residues were washed with water (2x5 mL) on a filter funnel. A sample (0.45 μL) of the filtrate ("reaction mixture") was transferred into a NMR tube together with D_2O (0.05 μL) and NMR experiments were performed. The formation of thioester **8** (sodium 2-((3,4-dicarboxy-3-hydroxybutanoyl)thio)ethanesulfonate) was evidenced by the ^{13}C NMR analysis (Fig. S21) and HRMS analyses. **^{13}C NMR** (100 MHz, D_2O): δ_c 198.4 (*C*OS), 178.1 (*C*OO), 174.9 (*C*OO), 74.3, 51.4, 50.2, 43.7, 23.7.

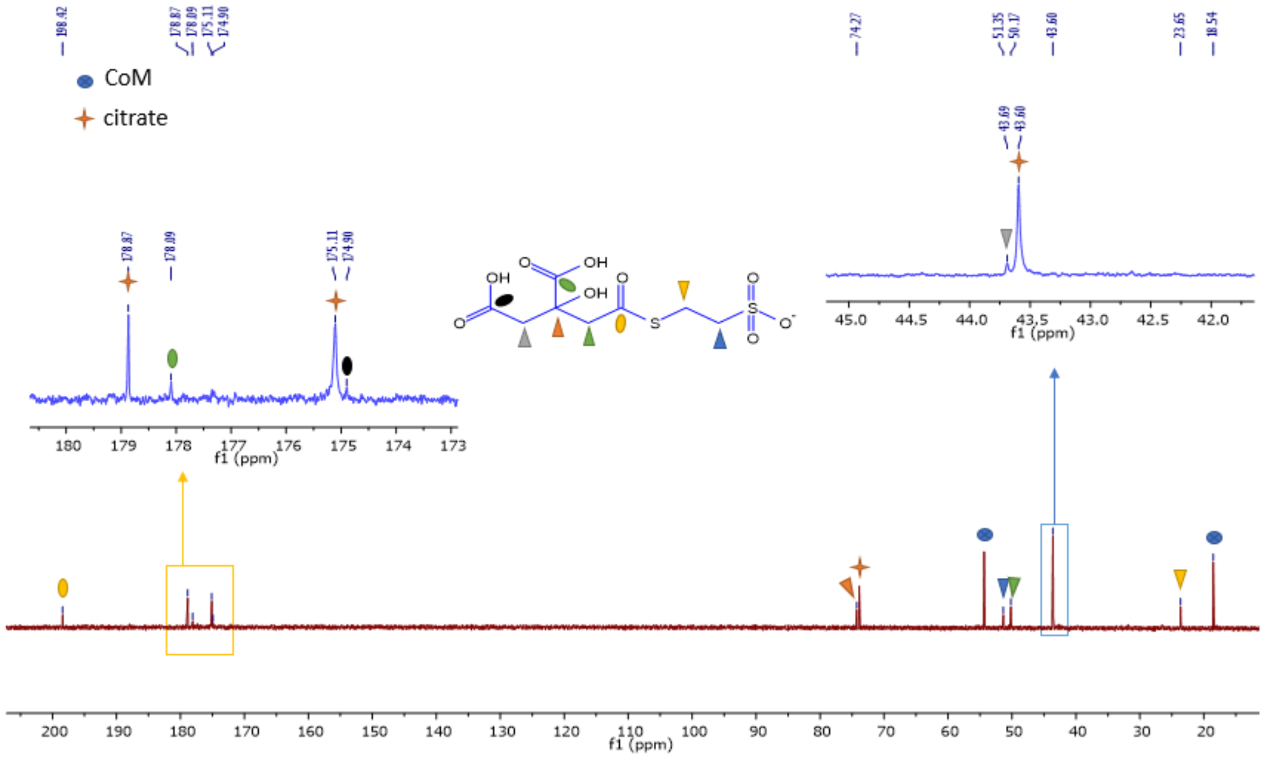

Fig. S21. ^{13}C NMR spectrum (D_2O) of the reaction mixture for the dehydration of citric acid over silica in the presence of 1 equivalent CoM (Table S5, Entry 18).

9. Reaction of succinic anhydride 1 with sodium sulfide

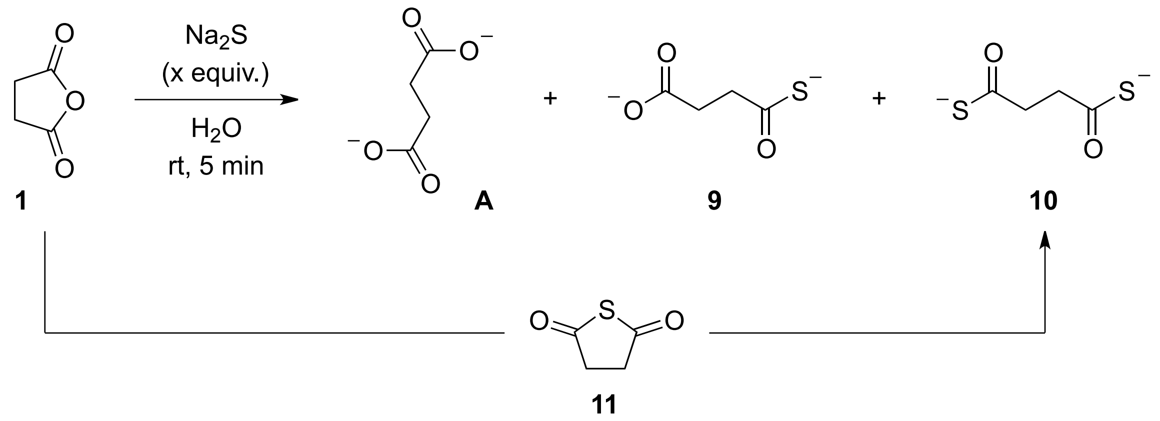

Products **9** (sodium 4-oxo-4-sulfidobutanoate) and **10** (sodium butanebis(thioate)), obtained when succinic anhydride **1** reacts with sodium sulfide, were identified through analysis of low resolution mass spectra, ^{1}H and especially ^{13}C NMR spectra of:

- reaction mixtures of succinic anhydride **1** and sodium sulfide in water (Table S6),
- aqueous solutions of thioacetic acid at different pH (Fig. S24),
- reaction mixtures of thioanhydride **11** (thiolane-2,5-dione) and sodium sulfide in water (Table S7).

Note. All reactions involving sodium sulfide were carefully carried out in a fume hood.

Table S6. Product distribution for the reaction of succinic anhydride **1** with Na_2S.

Entry	Na_2S x equiv.	Ratio (%) [a]			
		**1**	**A**	**9**	**10**
1	0.5	19	59	22	0
2	1	<1	14	79	6
3	2	0	33	65	2

[a] Ratio between succinic anhydride (**1**), succinic acid (**A**), thiocarboxylate (**9**) and dithiocarboxylate (**10**), determined from the ^{1}H NMR spectrum of the reaction mixture.

Example procedure (Table S6, Entry 3)

To a mixture of succinic anhydride **1** (100 mg, 1 mmol) and $Na_2S \bullet 9H_2O$ (480 mg, 2 mmol) was added water (1.5 mL). The reaction mixture (pH ≈ 12) was stirred at room temperature for 5 min under Argon. A sample of the reaction mixture (0.45 μL) was transferred into a NMR tube together with D_2O (0.05 μL) and NMR

experiments (with water suppression in the case of ^{1}H NMR) were performed. Formation of sodium 4-oxo-4-sulfidobutanoate **9** vas evidenced (Fig. S22-S23): ^{1}**H NMR** (400 MHz, D_2O): δ_H 2.93-2.89 (m, 2H), 2.47-2.43 (m, 2H). ^{13}**C NMR** (100 MHz, D_2O): δ_c 222.9 (*C*OS$^-$), 182.2 (*C*OO$^-$), 47.1, 35.1.

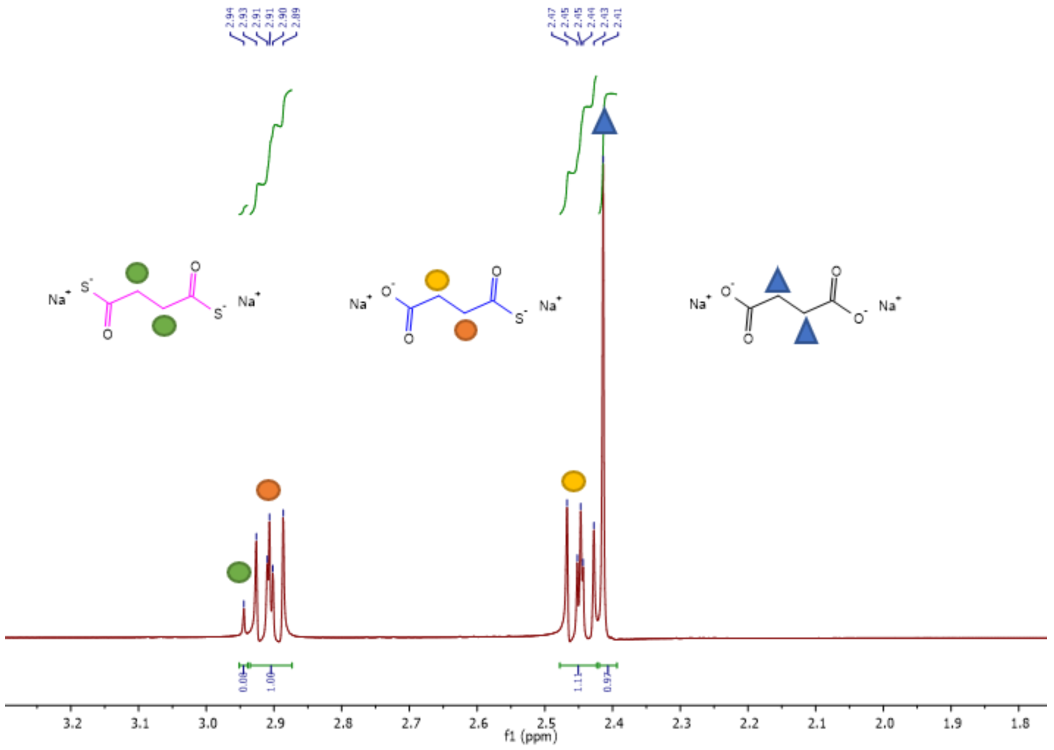

Fig. S22. ^{1}H NMR spectrum (D_2O) of the reaction mixture of succinic anhydride **1** with 2 equivalents of Na_2S in water (Table S6, Entry 3).

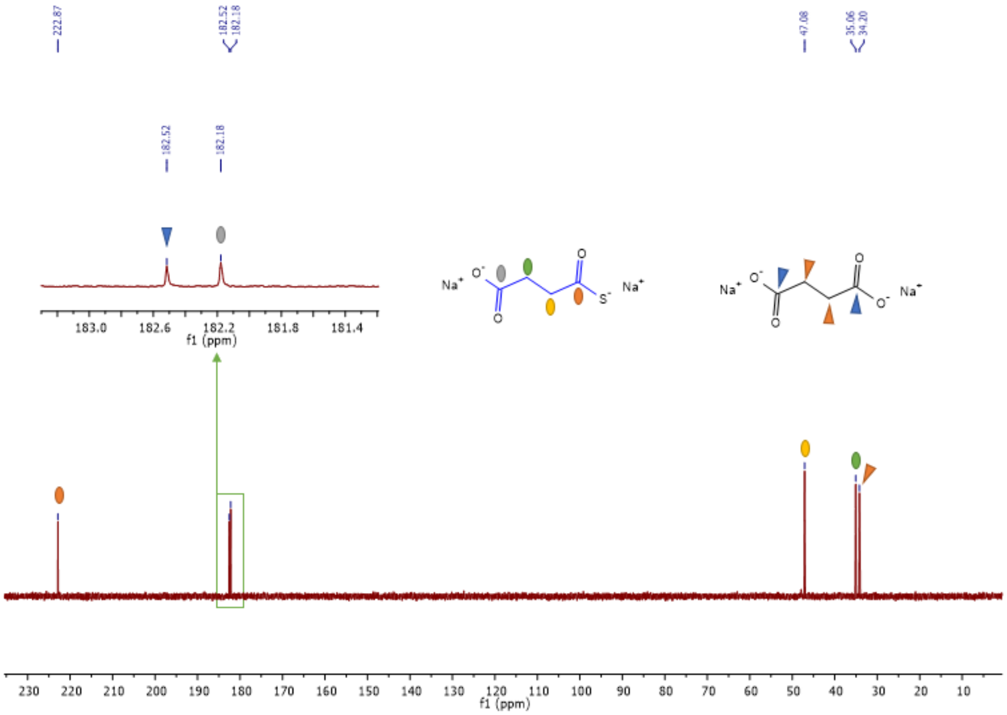

Fig. S23. ^{13}C NMR spectrum (D_2O) of the reaction mixture of succinic anhydride **1** with 2 equivalents of Na_2S in water (Table S6, Entry 3).

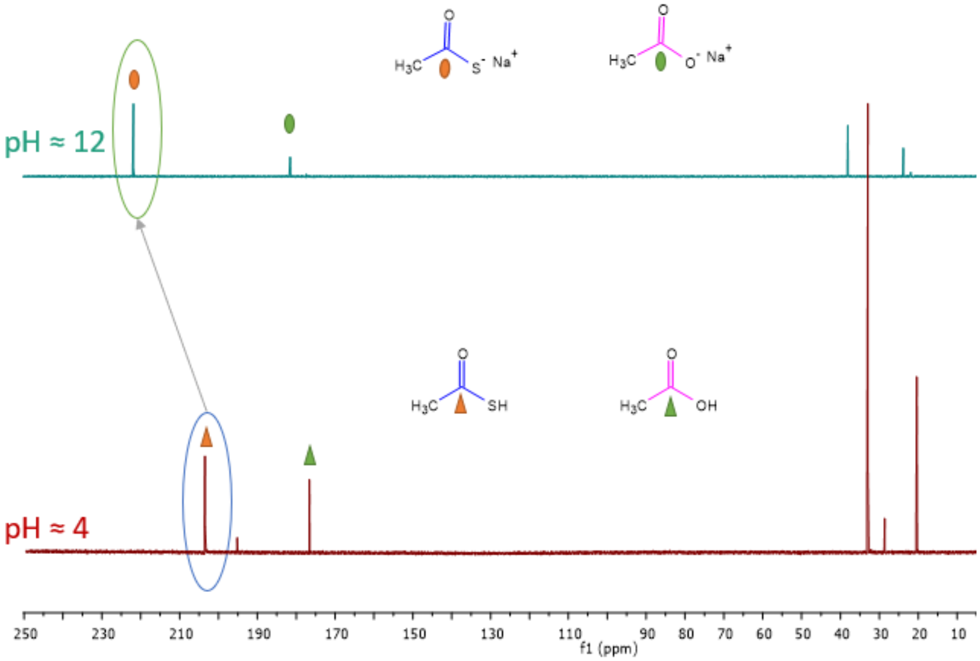

Fig. S24. ^{13}C NMR spectra (D_2O) of thioacetic acid (contaminated with acetic acid): (*bottom*) recorded directly (pH ≈ 4); (*top*) recorded after addition of a solution of NaOH (1M) up to pH ≈ 12. The *C*OS signal shifted from 203.5 ppm (pH ≈ 4) to 221.9 ppm (pH ≈ 12).

Table S7. Reaction of succinic thioanhydride **11** with Na_2S.

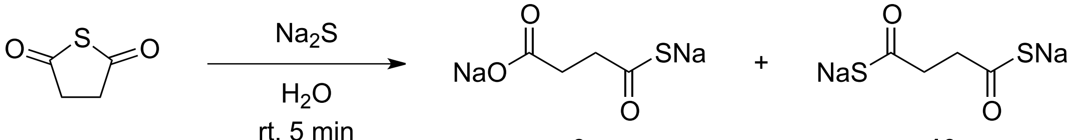

11 **9** **10**

Entry	Na_2S x equiv.	Conversion (%)[a] into:	
		9	10
1	1	26	52
2	1.5	22	78

[a] Ratio between succinic thioanhydride (**11**), thiocarboxylate (**9**) and dithiocarboxylate (**10**), determined from the ^{1}H NMR spectrum of the reaction mixture.

Example procedure (Table S7, Entry 2)

To a mixture of succinic thioanhydride **11** (thiolane-2,5-dione; 100 mg, 0.86 mmol) and $Na_2S{\bullet}9H_2O$ (311 mg, 1.3 mmol) was added water (1.5 mL). The reaction mixture (pH ≈ 12) was stirred at room temperature for 5 min under Argon. A sample of the reaction mixture (0.45 μL) was transferred into a NMR tube together with D_2O (0.05 μL) and NMR experiments (with water suppression in the case of ^{1}H NMR) were performed. Formation of sodium butanebis(thioate) **10** vas evidenced (Fig. S25-S26): **^{1}H NMR** (400 MHz, D_2O): δ_H 2.87 (s, 4H). **^{13}C NMR** (100 MHz, D_2O): δ_c 222.5 (*C*OS$^-$), 47.9.

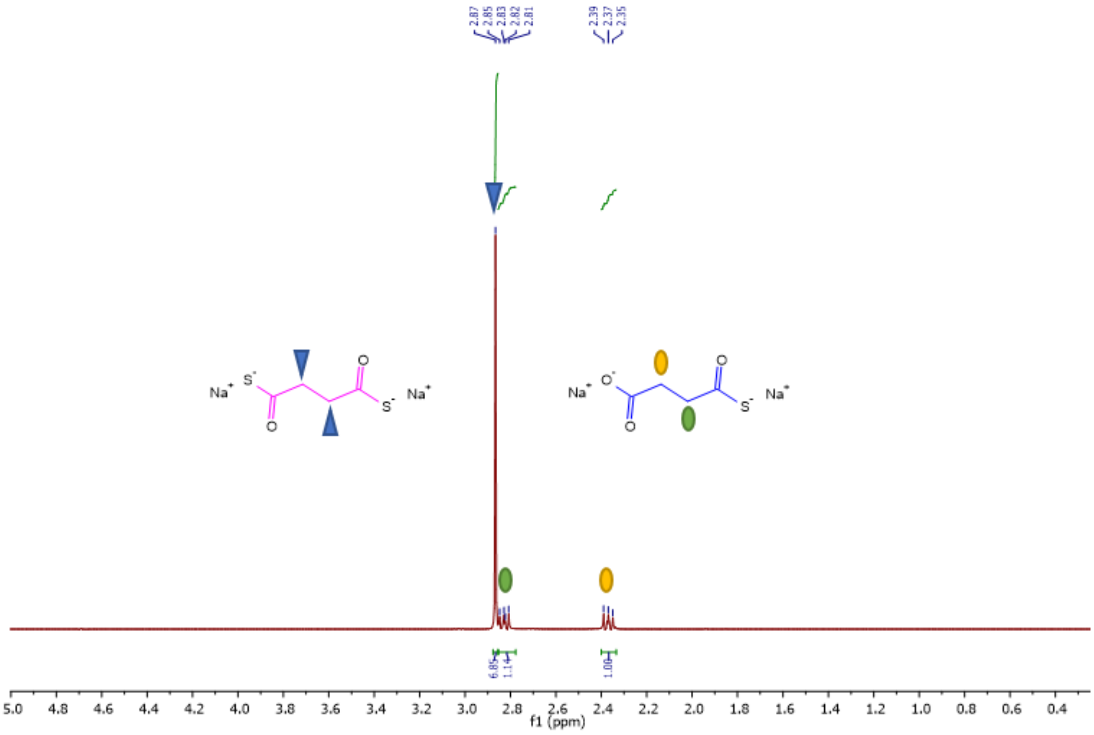

Fig. S25. ^{1}H NMR spectrum (D_2O) of the reaction mixture of succinic thioanhydride **11** with 1.5 equivalents of Na_2S in water (Table S7, Entry 2).

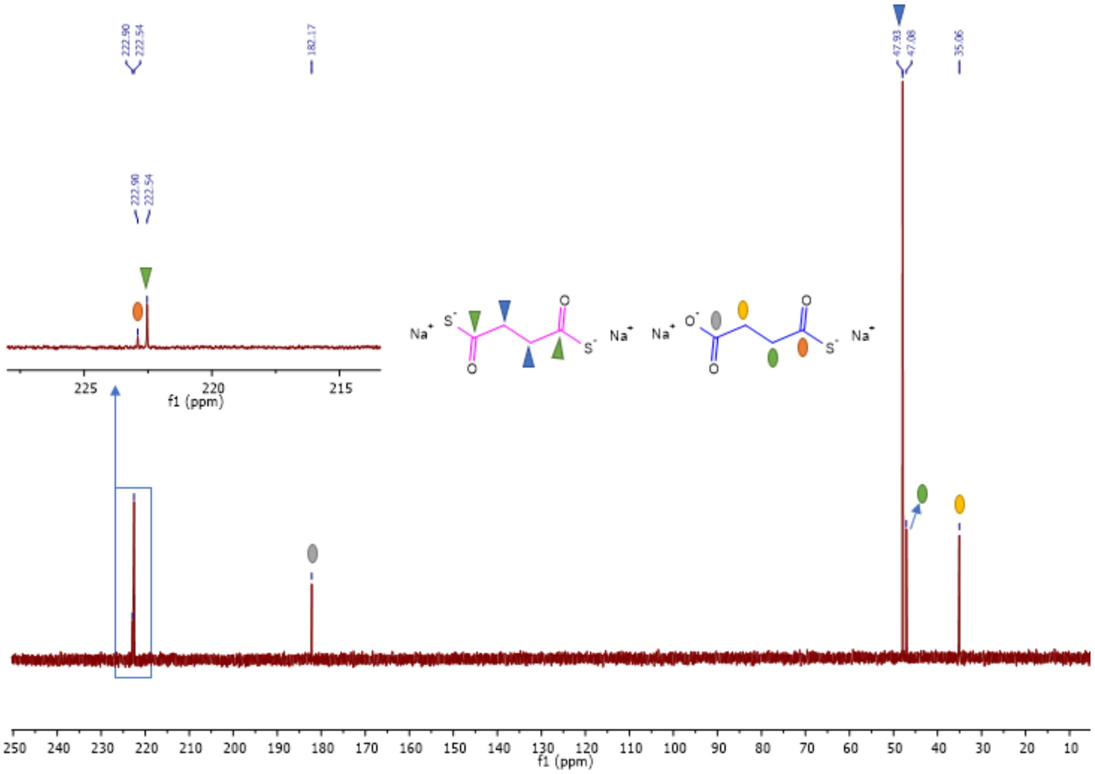

Fig. S26. ^{13}C NMR spectrum (D_2O) of the reaction mixture of succinic thioanhydride **11** with 1.5 equivalents of Na_2S in water (Table S7, Entry 2).

10. Reaction of citric anhydride 2 with sodium sulfide

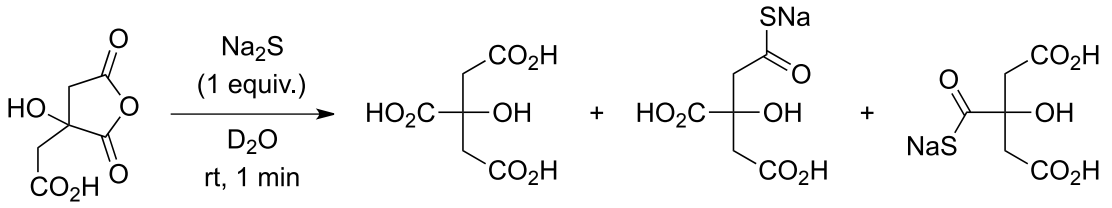

2 **B** **12** **13**

ratio: [a] 77% 11% 12%

[a] Estimated from the ^{13}C NMR spectrum of the reaction mixture.

Procedure: Citric acid anhydride (10 mg, 0.057 mmol), $Na_2S{\bullet}9H_2O$ (13.7 mg, 0.057 mmol) and D_2O (0.5 mL) were introduced into an NMR tube. The ^{13}C NMR spectrum was recorded immediately, showing complete disappearance of **2** and the presence of thiocarboxylates **12** (sodium 3,4-dicarboxy-3-hydroxybutanethioate) and **13** (sodium 3-carboxy-2-(carboxymethyl)-2-hydroxypropanethioate).

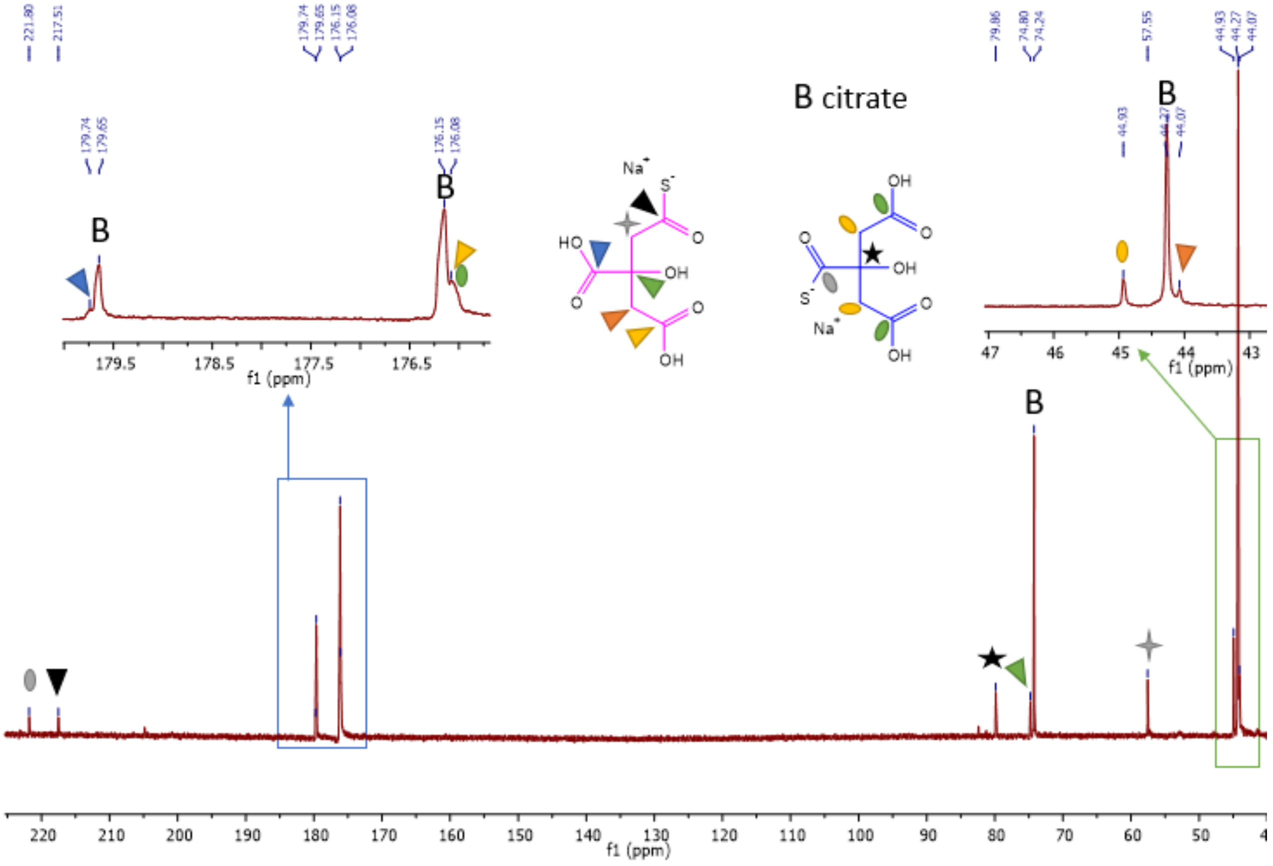

Fig. S27. ^{13}C NMR spectrum (D_2O) of the reaction mixture of citric anhydride **2** with 1 equivalent of Na_2S.

11 Computational details

A reported methodology FASTCAR[1] was applied to secure the lowest transition state (TS) and products. Briefly, it begins with the use of CREST, an automatic conformational search developed by Grimme and coworkers,[2] with an arbitrary initial TS. The conformers ensemble found was pruned using sPyRMSD.[3] The remaining geometries were then optimized at the B3LYP-D3BJ/DEF2SVP level.[4,5] Any redundant or incorrect geometries were discarded and the final ensemble was optimized at the B3LYP-D3BJ/DEF2TZVP level.

All optimizations conducted under Gaussian were conducted without constraints.[6] In order to ensure that the stationary point was of the appropriate nature in both cases, vibrational frequencies were systematically computed in the case of the transition state search and at the end of the search regarding the minimum. Finally, intrinsic reaction coordinate (IRC) calculations were performed to verify that the transition states connect the reagents and products. The starting point for every reaction path is the reactant, which is optimised alone, with the exception of zinc as a Lewis acid, where a complex with citric anhydride is taken as the starting point. Molecular structure images were generated using the CYLview2.0 software.[7]

12 Nucleophilic addition of MeSH to citric anhydride without Lewis acid

12.1 C_a selectivity

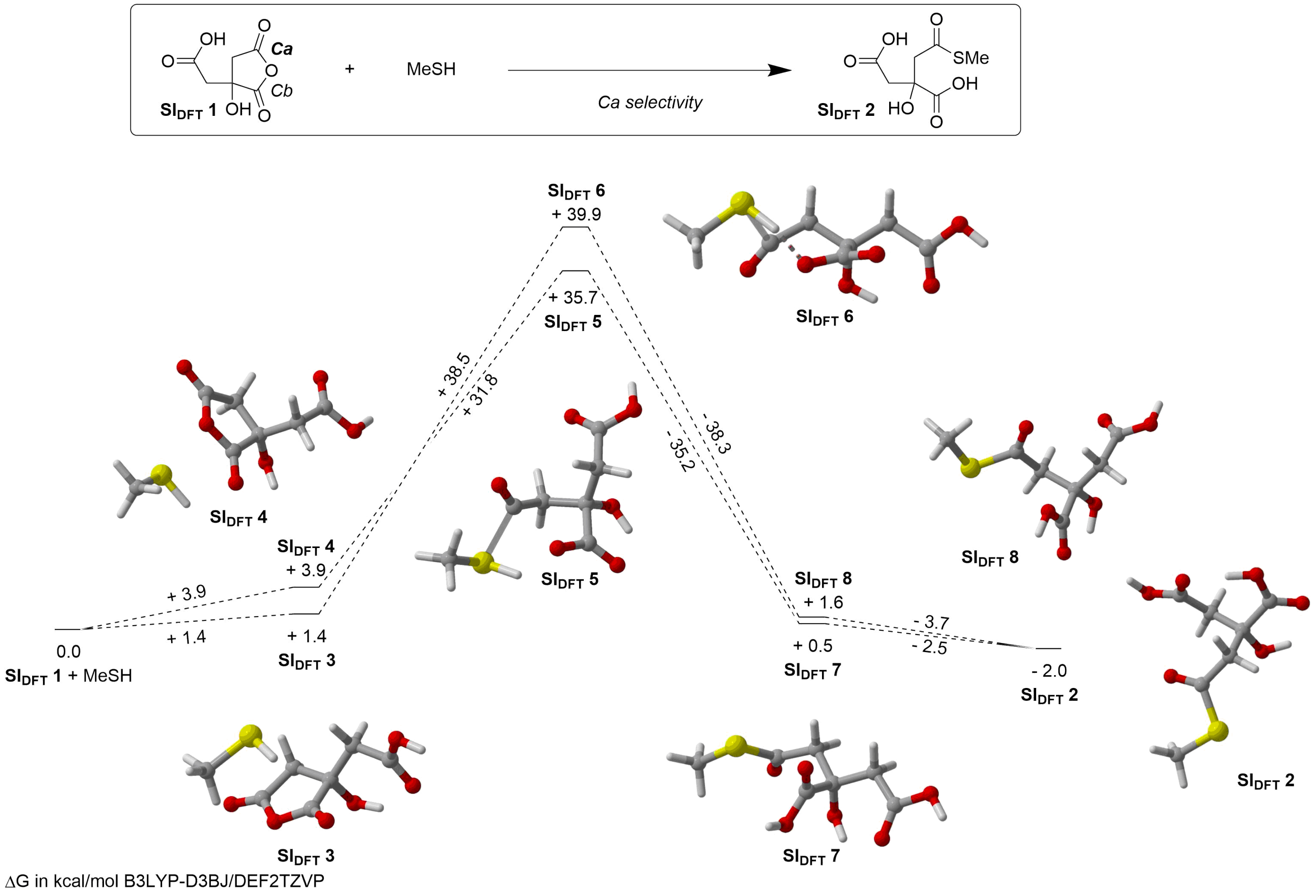

Figure S28: Nucleophilic addition of MeSH to the C_a site of citric anhydride without Lewis acid. The Gibbs free energies relative to the starting materials are given in kcal/mol.

MeSH
E(RB3LYP) = -438.755847914 A.U.
First frequency = 239.2599 cm^{-1}

C	-5.5524039929	-0.4436895877	-0.00076238
S	-3.7297443231	-0.4278743329	-0.0120583296
H	-5.953046562	-0.5763165201	-1.0035376894
H	-5.9530475023	0.4618866723	0.4498806092
H	-5.8440937987	-1.2971898806	0.6089082492
H	-3.5790897022	0.658018288	-0.787730881
H	-3.5790897022	0.658018288	-0.787730881

SI DFT 001
E(RB3LYP) = -683.943554931 A.U.
First frequency = 39.5727 cm^{-1}

C	-4.2897899951	0.3426114674	-0.1601333534
O	-3.0010336053	0.2500559709	0.3106321378
C	-2.4253330117	1.5119138766	0.466015973
C	-3.3763218202	2.5492204493	-0.0839229399
C	-4.722052795	1.8217331712	-0.1612740558
H	-3.3985490022	3.4312006282	0.5503326347
H	-3.0047725856	2.8448698257	-1.0684077925
O	-1.3433616467	1.6430790313	0.9309904435
O	-4.9249023453	-0.6048026854	-0.4874525079
O	-5.4581030436	1.9686673276	1.0403086563
H	-5.7833154131	2.8830968962	1.0493614396
C	-5.57373119	2.1287794921	-1.3890201896
H	-6.4286971369	1.4480564211	-1.4013514447
H	-5.0248276968	1.9549058268	-2.3155987644
C	-6.0982635312	3.5398856521	-1.3774485008
O	-6.1105473835	4.270242239	-0.4132385353
O	-6.5777225526	3.9112197574	-2.5733489345
H	-6.931590935	4.8114168026	-2.4848934861
H	-6.931590935	4.8114168026	-2.4848934861

SI DFT 002
E(RB3LYP) = -1122.72655915 A.U.
First frequency = 34.7960 cm^{-1}

C	-3.88092	-1.60775	-0.02351
S	-2.20466	-2.14389	0.39491
C	-1.38785	-0.58078	0.41291
O	-1.9454	0.46883	0.18002
C	0.08331	-0.65601	0.75743
C	0.98815	-0.24563	-0.43299
C	0.62862	1.11472	-1.0675
C	0.39795	2.26666	-0.1337
O	-0.37591	3.20967	-0.66409
C	2.45418	-0.30602	0.06575
O	2.84977	0.59702	0.94716
O	3.18188	-1.1818	-0.33332
O	0.85198	-1.19213	-1.46587
O	0.88825	2.38545	0.97495
H	0.24182	0.00665	1.60848
H	0.3659	-1.66782	1.04717
H	1.68053	-1.70419	-1.46453
H	-0.26169	0.97901	-1.67552
H	1.43262	1.40186	-1.75194
H	-0.46517	3.92539	-0.01389
H	-4.49974	-2.50259	-0.04022
H	-4.24899	-0.91095	0.72553
H	-3.88607	-1.13104	-1.00069
H	2.14575	1.27623	1.11172
H	2.14575	1.27623	1.11172

SI DFT 003
E(RB3LYP) = -1122.71433276 A.U.
First frequency = 22.0521 cm^{-1}

C	0.0623670704	0.1674977774	-1.0482930049
O	-1.1337369117	0.7611632068	-1.3798648166
C	-1.4733498692	1.746760755	-0.453679145
C	-0.3988555741	1.823473453	0.6040626591
C	0.7570163408	1.0079849707	0.0426567628
H	-0.1170100512	2.8567386403	0.7932411497
H	-0.8066207791	1.3875772547	1.5162784312
O	-2.4906606363	2.3496077971	-0.5475608596

O	0.4588658003	-0.8144491981	-1.5910867494
O	1.6372905346	1.9052176418	-0.6084657543
H	2.4359575156	1.4006685872	-0.8496142187
C	1.4722489567	0.1228923434	1.0625985427
H	0.7691016068	-0.5581604907	1.5365578212
H	1.8980637247	0.7691323895	1.8348469761
C	2.6158303916	-0.6485902341	0.4491751207
O	3.3991983971	-0.1931984552	-0.3516543148
O	2.7052256543	-1.8986901831	0.921337605
H	3.4660093892	-2.3210739762	0.4896109454
S	-2.0202568787	-1.5432172751	1.1111998783
H	-1.3389601507	-2.3155277771	0.2494405446
C	-3.4537550257	-1.2012276198	0.03569186
H	-3.1354594035	-0.7639246137	-0.906487262
H	-4.0282974819	-2.1082788232	-0.1352926492
H	-4.0713002282	-0.4814511739	0.5691717089
H	-4.0713002282	-0.4814511739	0.5691717089

SI DFT 004
E(RB3LYP) = -1122.70877044 A.U.
First frequency = 22.8109 cm^{-1}

C	-0.7030149936	-1.7750930987	-0.8976316642
O	-1.0817045534	-0.5696638905	-1.5200515072
C	-0.4606883793	0.4899124731	-0.925095614
C	0.660003841	-0.0144881265	-0.0078080419
C	0.2744253245	-1.4735239699	0.2098267691
O	-0.7515421158	1.6265286586	-1.1395967322
O	-1.1518649685	-2.806962718	-1.2645661202
S	-2.9335570722	-0.0546050347	1.3398844371
H	-2.4089781112	1.1477284606	1.6225312426
C	-3.9552072765	0.4598468259	-0.0793531093
H	-4.3750544415	-0.4537938622	-0.4957174447
H	-4.7647094988	1.1114728285	0.241258557
H	-3.3482001304	0.9478462762	-0.8377340645
H	-0.2556873964	-1.5739107029	1.1568907522
H	1.1087963661	-2.1695199404	0.1957051007
C	1.9898932928	0.1685656875	-0.7750204896

H	1.9851498923	-0.4419931406	-1.6796720339
H	2.0942298208	1.2144009313	-1.0597152206
C	3.1581243595	-0.2648922862	0.0787632518
O	3.4023173278	-1.4037318557	0.3805522037
O	3.9064328546	0.7805916941	0.4787161293
H	4.6190596071	0.4294386059	1.0372064446
O	0.6787651156	0.6929830096	1.2092231264
H	0.6668971357	1.639220175	1.0105110277
H	0.6668971357	1.639220175	1.0105110277

SI DFT 005
E(RB3LYP) = -1122.66104448 A.U.
First frequency = -225.1721 cm^{-1}

C	-1.061606898	0.878547402	-0.0746475862
O	-1.0809769063	-1.1109893013	0.9498567553
C	-0.0834812109	-1.6739147441	0.3804311883
C	0.8805122712	-0.6577752006	-0.2950992876
C	-0.0305963148	0.3595850808	-1.0190884821
O	0.1753526931	-2.8645968332	0.2690413261
O	-1.0956023144	1.7294964594	0.723196271
S	-3.006729248	0.1656888954	-0.6719001611
H	-2.52987774	-0.8964889594	0.0788636489
C	-3.9720830201	0.9902001416	0.6134898648
H	-4.5758605086	1.7580738076	0.1372957279
H	-4.6108203015	0.2605565138	1.103598455
H	-3.2812830971	1.4384586313	1.3274522904
H	-0.4948932349	-0.1442856818	-1.8632482915
H	0.5454347667	1.2192829152	-1.3648020462
C	1.7722493961	-0.0175603223	0.7825279234
H	1.1652331076	0.5489817872	1.4908821229
H	2.2747444949	-0.8154407842	1.3253125394
C	2.7950342592	0.9208401801	0.1929443004
O	2.550665435	1.8495921862	-0.5379796066
O	4.0471615771	0.6272943828	0.591195536
H	4.6382580095	1.2758850612	0.1757974089
O	1.6759039978	-1.3189429052	-1.2424349664
H	1.5202716899	-2.2659367497	-1.0575689317

H	1.5202716899	-2.2659367497	-1.0575689317

SI DFT 006
E(RB3LYP) = -1122.65451198 A.U.
First frequency = -490.5676 cm^{-1}

C	0.3243008051	-0.6654056128	-0.7137383941
O	-0.9025357994	-0.542269643	-1.1078886695
C	-1.7274175375	0.8493696346	0.3339969889
C	-0.4953629775	0.6975438178	1.1829353883
C	0.7244728939	0.4653102991	0.2873605024
H	-0.3821990713	1.6274838938	1.7421832494
H	-0.6229921541	-0.1328227353	1.8748544501
O	-2.2450836543	1.7563485052	-0.1853724192
O	1.1229539743	-1.52963087	-1.0223588954
O	0.9549181648	1.6792367173	-0.3972883316
H	1.8165361131	1.5888750446	-0.8421712699
C	1.9425017422	0.0397085619	1.1132333995
H	1.7474245051	-0.8846499095	1.6527434374
H	2.1770912787	0.8246618011	1.8379098534
C	3.1628758673	-0.1385124394	0.2433285037
O	3.4636355807	0.5870403354	-0.6771613176
O	3.9406968707	-1.1511656519	0.64886047
H	4.6972666801	-1.1933841079	0.0420638603
S	-3.0048785439	-0.8575083494	0.6355204765
H	-2.0405517728	-1.2163486062	-0.3578893826
C	-4.345417976	-0.3344853483	-0.4587242937
H	-4.0799002058	0.6366499972	-0.8785089184
H	-4.4715217564	-1.0668964559	-1.2512150006
H	-5.2550120468	-0.2559703982	0.1305860944
H	-5.2550120468	-0.2559703982	0.1305860944

SI DFT 007
E(RB3LYP) = -1122.71896856 A.U.
First frequency = 20.3100 cm^{-1}

C	-1.2294310867	-0.7933714926	0.2256352246
O	-0.6318094481	1.5384507732	-1.4968049616

C	0.0702397256	1.9393983126	-0.4299227602
C	0.821789402	0.8035884156	0.270134231
C	-0.2119966001	-0.0466741207	1.0584596517
O	0.0896786449	3.0765343568	-0.0313284126
O	-0.9459323899	-1.589041256	-0.6347781225
S	-2.902990054	-0.3797973027	0.683944638
H	-1.1219353149	2.3042741253	-1.8363110652
C	-3.8400263706	-1.448580675	-0.4364814805
H	-4.4081403728	-2.1749564548	0.1400908206
H	-4.5139743142	-0.839714329	-1.0348608142
H	-3.1210900945	-1.9576330047	-1.0773475378
H	-0.708310487	0.6104498037	1.7730695327
H	0.3666586925	-0.7869511076	1.6106962965
C	1.6434163209	0.0134531067	-0.7527946871
H	0.9837585436	-0.4952681947	-1.4560927767
H	2.2700871194	0.703890918	-1.31632992
C	2.5295813225	-1.0437854091	-0.140163478
O	2.3299193066	-1.6609283666	0.8719102022
O	3.6108370985	-1.2650949578	-0.9213834018
H	4.1152096944	-1.9832914725	-0.5072654575
O	1.7088347511	1.3851250186	1.2008960977
H	1.445481277	2.3122840724	1.3061689396
H	1.445481277	2.3122840724	1.3061689396

SI DFT 008
E(RB3LYP) = -1122.71830856 A.U.
First frequency = 23.2187 cm^{-1}

C	0.3380634594	-1.0510106196	-0.4539477517
O	-0.0414521728	-0.9560530286	-1.7329777913
C	-1.8837369953	0.8171570322	0.6385586229
C	-0.5104978252	0.5915749034	1.2473236277
C	0.5992071679	0.3141081904	0.2112452275
H	-0.2467325	1.5082214229	1.7745307314
H	-0.5418306485	-0.2386664236	1.9528356276
O	-2.3118299577	1.9031303635	0.3511808186
O	0.4661680852	-2.0956272599	0.1319129116
O	0.5985073001	1.3821315504	-0.699310793

H	1.2388407946	1.1728738164	-1.3940419558
C	1.9385128842	0.2181735884	0.9641508907
H	1.9191540742	-0.5870825412	1.6980461296
H	2.1095658797	1.1530886466	1.5016287146
C	3.1033383338	-0.0061996597	0.0380500895
O	3.0445996127	-0.0143588602	-1.1684027691
O	4.2519366629	-0.1923242964	0.7102097316
H	4.9569277845	-0.3185047132	0.0548171969
S	-2.823928493	-0.6842031224	0.4172573712
H	-0.1934965361	-1.8602296679	-2.0507774885
C	-4.2820750614	0.0093887742	-0.3981066583
H	-4.7495448636	0.7518250268	0.2437496151
H	-3.9977985346	0.4703072635	-1.340998106
H	-4.9653600919	-0.8188881082	-0.5749184949
H	-4.9653600919	-0.8188881082	-0.5749184949

12.2 C_b selectivity

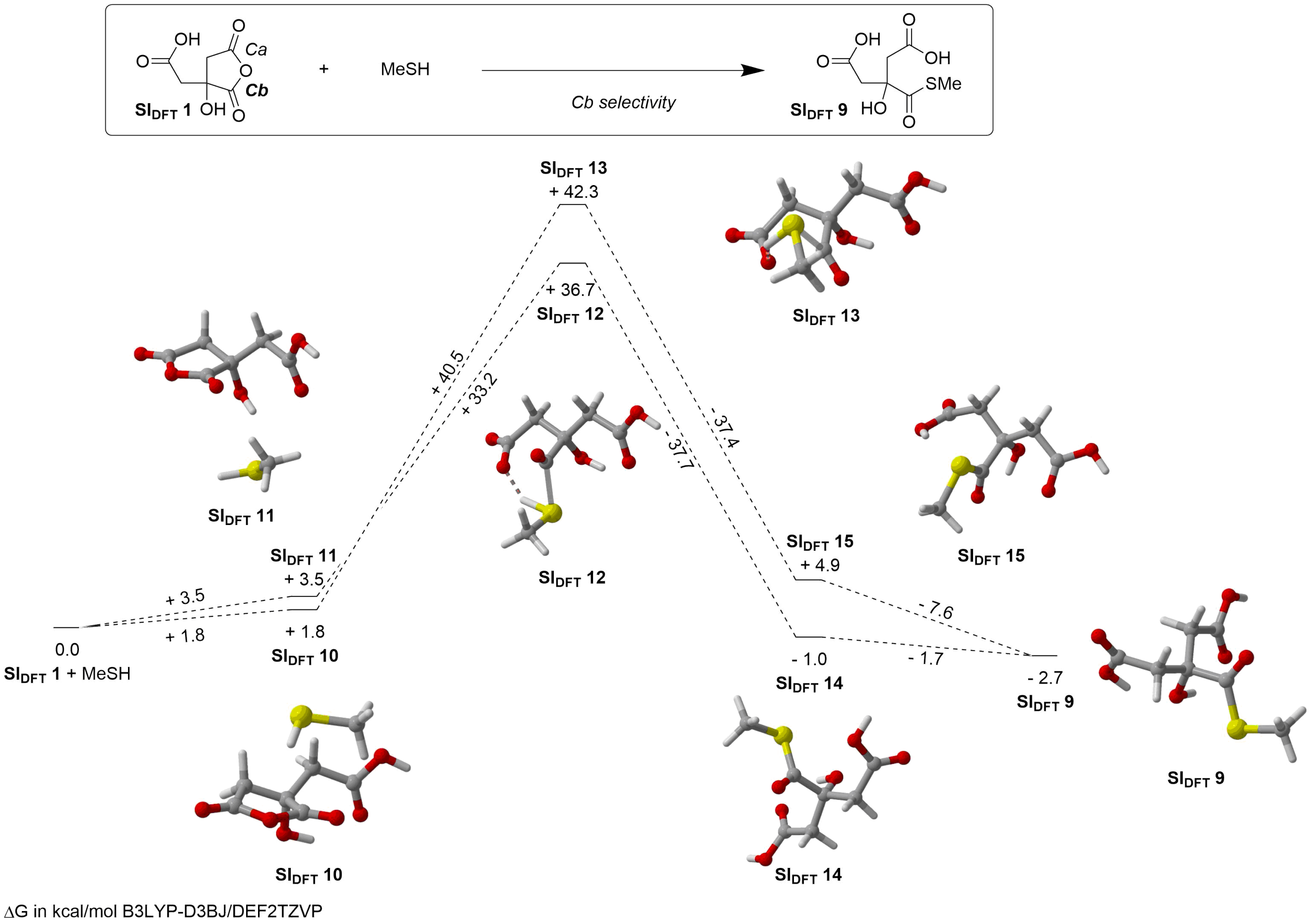

Figure S29: Nucleophilic addition of MeSH to the C_b site of citric anhydride without Lewis acid. The Gibbs free energies relative to the starting materials are given in kcal/mol.

SI DFT 009
E(RB3LYP) = -1122.72733325 A.U.
First frequency = 40.8383 cm^{-1}

C	-0.05029	1.13148	-0.68303
O	-1.62843	-2.15842	1.76886
C	-2.04868	-1.98405	0.50411
C	-1.68085	-0.65113	-0.14215
C	-0.23283	-0.19358	0.08701
H	-1.87846	-0.73434	-1.20784
H	-2.34393	0.11526	0.26785
O	-2.69333	-2.82064	-0.06361
O	0.18019	1.1508	-1.86709
O	-0.07637	0.01589	1.48681
H	0.87524	0.16078	1.64588
C	0.76348	-1.22921	-0.45141
H	0.64492	-1.33982	-1.52712
H	0.56552	-2.20384	0.00288
C	2.19128	-0.87036	-0.13981
O	3.06463	-1.50318	-0.92944
O	2.53669	-0.11411	0.74253
S	-0.26752	2.57794	0.31137
C	-0.08541	3.81667	-0.99686
H	-0.84969	3.67958	-1.75786
H	-0.20238	4.78694	-0.51833
H	0.89925	3.74026	-1.45125
H	-1.07804	-1.3939	2.0356
H	3.95927	-1.25026	-0.64813
H	3.95927	-1.25026	-0.64813

SI DFT 010
E(RB3LYP) = -1122.71447786 A.U.
First frequency = 30.9340 cm^{-1}

C	-0.4903712073	-0.3339801342	0.9899404729
O	-1.832293805	-0.6192801908	1.0439541656
C	-2.2192184698	-1.4026041213	-0.0463131884
C	-1.0247145241	-1.6120642234	-0.9465715861

C	0.1704712739	-1.2059353643	-0.0972341141
H	-0.9652687785	-2.6456681474	-1.2787294581
H	-1.149992092	-0.960402584	-1.8123927562
O	-3.3406559776	-1.7642432655	-0.1737196593
O	0.0301550444	0.4384078484	1.7326711293
O	0.6609377378	-2.3732911653	0.5361644871
H	1.4973404123	-2.133721503	0.9753166355
C	1.2621488615	-0.4433568341	-0.8462373828
H	0.8468132022	0.4444134998	-1.3165968651
H	1.6588491915	-1.0970521911	-1.6275766522
C	2.4172233104	-0.0715399161	0.0506852522
O	2.9056906333	-0.8023732936	0.8804978763
O	2.88694645	1.1601259667	-0.1961091548
H	3.6308948626	1.3144376556	0.4091609288
S	-1.4477602701	2.2028759302	-1.1421531415
H	-2.3800581562	2.0291824959	-0.1916742898
C	-0.2801567529	3.1909821548	-0.1479996008
H	-0.7146858192	4.1548873117	0.1055567547
H	0.0139388764	2.6520496883	0.7485051125
H	0.5960183086	3.3482141211	-0.7742895057
H	0.5960183086	3.3482141211	-0.7742895057

SI DFT 011
E(RB3LYP) = -1122.71073371 A.U.
First frequency = 21.7608 cm^{-1}

C	0.7539262084	-0.5407469222	1.0257954792
O	1.9194240104	-1.2292828638	1.2561747847
C	1.9288794161	-2.4431196965	0.5601172464
C	0.5834717365	-2.6300585772	-0.1006595854
C	-0.0305106291	-1.233990027	-0.1061706095
H	0.6962657088	-3.0344693297	-1.1033848948
H	0.0098858925	-3.3363262147	0.5031731402
O	2.8755585637	-3.1545735681	0.5798010474
O	0.4726188521	0.4528800606	1.6191985033
O	0.3507120035	-0.6408678134	-1.3253079638
H	0.1989795701	0.3265200874	-1.3099430775
C	-1.5412771556	-1.2217144664	0.1414209161

H	-1.780990268	-1.7811806162	1.0444380437
H	-2.0150641534	-1.7193441307	-0.7080434603
C	-2.1369268267	0.1636513701	0.2364054596
O	-1.9558082223	1.0546267531	-0.5520962153
O	-2.9498521525	0.2831443843	1.3036505499
H	-3.3061937455	1.1860360159	1.2954004348
S	0.9842691747	2.5736023693	-1.4558581958
H	2.1861897205	2.3916855452	-0.8843303154
C	0.1725679258	3.389240633	-0.043329541
H	-0.8638050543	3.5294096352	-0.3404054324
H	0.2050310108	2.7520410717	0.8356922649
H	0.6312473743	4.3551112273	0.1553262524
H	0.6312473743	4.3551112273	0.1553262524

SI DFT 012
E(RB3LYP) = -1122.65887683 A.U.
First frequency = -621.1073 cm^{-1}

C	0.0987996142	-0.3984297721	-0.6036098672
O	2.1831362032	-0.2999958699	-0.3973457671
C	2.4119222716	0.9255818171	0.0089283546
C	1.1451188227	1.7816096921	-0.1142264518
C	-0.0716735613	0.9229293977	0.2007687295
H	1.1859431903	2.6322515857	0.5630594427
H	1.0850551534	2.149646394	-1.1408336307
O	3.4607503655	1.3703746039	0.412966325
O	-0.226985287	-0.6329032857	-1.7122284125
O	-0.0656403329	0.6598547632	1.580819962
H	-0.9166899573	0.2447210979	1.8007546636
C	-1.3732622	1.5954801212	-0.2594340721
H	-1.3605353708	1.7576804842	-1.3361332404
H	-1.4441018448	2.5770432246	0.2164238948
C	-2.6111984627	0.8208354854	0.106575798
O	-2.6566447074	-0.0685271879	0.9271439634
O	-3.6903873171	1.2386706997	-0.5665364923
H	-4.4520195829	0.7196109462	-0.2601479278
S	0.3645123959	-1.9725939724	0.6851700231
H	1.6607892798	-1.3740861039	0.5148990754

C	0.4901679409	-3.2587527392	-0.5815487762
H	-0.2341781313	-4.0350653304	-0.3504263956
H	0.2680219304	-2.8067593598	-1.5489059388
H	1.4966515774	-3.6680090273	-0.5813856481
H	1.4966515774	-3.6680090273	-0.5813856481

SI DFT 013
E(RB3LYP) = -1122.64964852 A.U.
First frequency = -855.4773 cm^{-1}

C	-0.0970499365	-0.4495767388	-0.59985816
O	-2.1257759651	-0.6675842975	-0.4926490305
C	-2.5435814638	0.4324407453	0.1117760311
C	-1.3809386394	1.2060495449	0.7426195675
C	-0.1152434648	1.0108449911	-0.072762382
H	-1.6161897007	2.2655205233	0.8091236305
H	-1.250214353	0.8252942728	1.7595948019
O	-3.691027487	0.7841326862	0.2233104927
O	0.342993443	-0.9193265348	-1.5741273374
O	-0.2036105233	1.8500698519	-1.1890031742
H	0.6402267342	1.7999904535	-1.6704528571
C	1.1596233398	1.27101856	0.7502112938
H	1.2286812183	0.5988280743	1.6025819967
H	1.0872845772	2.2889116895	1.142858465
C	2.4331262668	1.2000852223	-0.0557327983
O	2.5270584128	1.4349101744	-1.2364492737
O	3.4891518576	0.8646042326	0.7027360559
H	4.2759464717	0.8778558438	0.1330286839
S	-0.2073859263	-1.8554746179	1.0822334497
H	-1.5111980101	-1.5391243226	0.470326445
C	0.2322050803	-3.3330434825	0.1360801882
H	-0.5861264201	-4.0466905281	0.1805015881
H	0.4171614339	-3.0384047086	-0.8978778984
H	1.129046204	-3.7647389885	0.5725306393
H	1.129046204	-3.7647389885	0.5725306393

SI DFT 014
E(RB3LYP) = -1122.72300364 A.U.
First frequency = 20.1086 cm^{-1}

C	0.1280455737	0.6630170169	0.573729986
O	2.8305665093	-0.472979238	0.4470495719
C	2.2442331388	-1.6121390152	0.0165785999
C	0.8380244944	-1.7584357191	0.5470890009
C	-0.1602124588	-0.7360846904	-0.0243341415
H	0.5027866122	-2.7574910428	0.2776296747
H	0.8527167614	-1.6573035812	1.6330214315
O	2.8058752292	-2.4128280497	-0.6795653162
O	-0.0009372136	0.8789672454	1.7545239624
O	-0.0252877186	-0.7488284065	-1.4242867752
H	-0.7974568914	-0.2900622647	-1.7965696619
C	-1.5742784269	-1.1537760307	0.4259228379
H	-1.6525859583	-1.1160606579	1.5103477274
H	-1.7498154917	-2.1860051601	0.1104364642
C	-2.6686209507	-0.3198906529	-0.1868699128
O	-2.5943120128	0.2597072059	-1.2458897427
O	-3.7814206056	-0.3102840308	0.5626731561
H	-4.4502112476	0.2096667352	0.0876165711
S	0.6105996929	1.8814034125	-0.6105131641
H	3.7084379752	-0.4305114184	0.036925776
C	0.8030857813	3.2739354858	0.5295364646
H	1.1095035394	4.1266711358	-0.0732209867
H	-0.1380699744	3.4887646291	1.0299028077
H	1.5644668947	3.0461745132	1.2718795034
H	1.5644668947	3.0461745132	1.2718795034

SI DFT 015
E(RB3LYP) = -1122.71377112 A.U.
First frequency = 31.1867 cm^{-1}

C	-0.0376125053	0.6269508669	0.5306110439
O	-2.9318588004	-0.1965696212	0.1672289266
C	-2.2591541776	-1.352978898	-0.0520588331
C	-1.0031017339	-1.2423400776	-0.9039239046
C	0.2526831349	-0.7283451057	-0.1491010847
H	-0.768040029	-2.2455588408	-1.2531262848
H	-1.1978813211	-0.6072086277	-1.7706320393
O	-2.6643857043	-2.3835567259	0.3983959246

O	-0.0544573258	0.7288672175	1.7280626453
O	0.6158556994	-1.6481494724	0.8465177042
H	0.4713921141	-1.2204385842	1.7059828894
C	1.4070980929	-0.6369941957	-1.1571559468
H	1.1481170061	0.0204001926	-1.9907381775
H	1.5874167042	-1.6301754431	-1.5650720154
C	2.6749498737	-0.1010406501	-0.5400480726
O	2.7248880998	0.7583770117	0.3000502233
O	3.7712961439	-0.6732997249	-1.0734860349
H	4.5447648128	-0.2619286348	-0.6551108066
S	-0.4641733766	1.9684039264	-0.5625773298
H	-2.4794236941	0.5566890997	-0.2474909795
C	-0.7107614691	3.2685658345	0.6735185004
H	-1.0316055691	4.1538109717	0.1287386973
H	-1.4709292804	2.9650835315	1.3880962708
H	0.2280674486	3.4590978427	1.1869449652
H	0.2280674486	3.4590978427	1.1869449652

13 Nucleophilic addition of MeSH to citric anhydride with $Zn^+Cl \cdot H_2O$ as Lewis acid

13.1 C_a selectivity

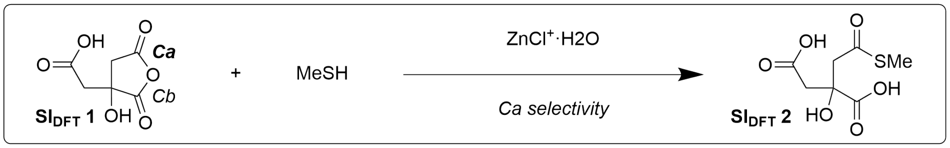

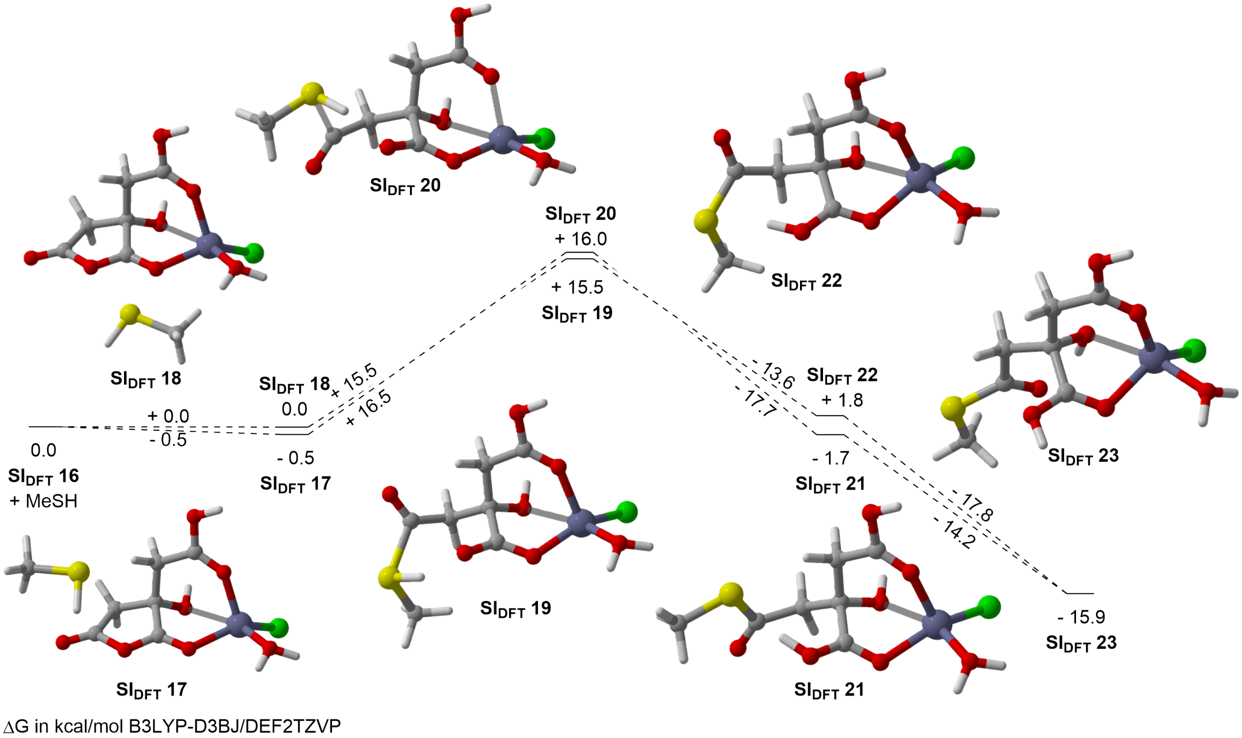

Figure S30: Nucleophilic addition of MeSH to the C_a site of citric anhydride with $ZnCl^+H_2O$ as Lewis acid. The Gibbs free energies relative to the starting materials are given in kcal/mol.

SI DFT 016

E(RB3LYP) = -2999.96182137 A.U.

First frequency = 32.5636 cm^{-1}

C	1.33284	-0.70482	-0.496
16 O	2.53623	-1.06168	-0.89367
C	3.52424	-0.73823	0.13615
C	2.75749	-0.24423	1.33834
C	1.39068	0.16393	0.77083
H	2.63476	-1.08434	2.02683
H	3.30977	0.54361	1.84585
O	4.66173	-0.89961	-0.07582
O	0.31311	-1.05	-1.05656
O	0.26435	-0.24185	1.50973
H	0.12503	0.27562	2.31402
C	1.36647	1.66479	0.42947
H	2.21662	1.93741	-0.20155
H	1.50207	2.23419	1.3539
C	0.11742	2.19154	-0.23971
O	-0.87288	1.54815	-0.54785
O	0.21129	3.48261	-0.46808
H	-0.60224	3.81414	-0.88942
Zn	-1.51348	-0.40967	-0.22805
Cl	-3.04127	-0.85676	1.2077
O	-2.37987	-0.74432	-2.10476
H	-3.30266	-1.02997	-2.05472
H	-1.94842	-1.24624	-2.80801
H	-1.94842	-1.24624	-2.80801

SI DFT 017

E(RB3LYP) = -3438.73483281 A.U.

First frequency = 16.5628 cm^{-1}

C	-0.4787830633	-1.0007038649	0.4156592494
O	-1.5989356414	-1.593450815	0.773649537
C	-2.5253662186	-1.6460415662	-0.3471845735
C	-1.8039970563	-1.0919078041	-1.5497387434
C	-0.5900168007	-0.3493516468	-0.9735365755

H	-1.4707949681	-1.9380484791	-2.15582326
H	-2.4797416671	-0.4823547604	-2.145170258
O	-3.5877926571	-2.1173507592	-0.2068296906
O	0.5247766266	-1.0151647738	1.0991717282
O	0.6541148496	-0.6445314636	-1.570799843
H	0.774448092	-0.2110011703	-2.4260272817
C	-0.8698820838	1.1577846662	-0.8875263736
H	-1.7999555596	1.3295760882	-0.3304408343
H	-1.0603864605	1.5374944178	-1.8954604208
C	0.1967817925	2.0242164288	-0.2743867989
O	1.2533548174	1.6553556381	0.2189792821
O	-0.1401837098	3.295008965	-0.3133309976
H	0.559507845	3.841905224	0.0864562687
Zn	2.2591406677	-0.1509635618	0.2839748388
Cl	3.9811661613	-0.5414634324	-0.9357645827
O	3.011490548	-0.0063672292	2.2383259859
H	3.9702724689	-0.1280329567	2.2776913675
H	2.6202637533	-0.4674325748	2.9911019255
S	-3.8825869882	1.1392386522	1.1608986546
C	-5.474982641	0.6782136894	0.399251236
H	-3.8156419267	0.1411202936	2.0569849498
H	-5.6580967679	1.4041477262	-0.3903900186
H	-6.2715294898	0.7485558513	1.1350301253
H	-5.4318353066	-0.3227706183	-0.0219705737
H	-5.4318353066	-0.3227706183	-0.0219705737

SI DFT 018
E(RB3LYP) = -3438.73569280 A.U.
First frequency = 31.6377 cm^{-1}

C	-1.1153771821	0.6361480939	0.7212691716
O	-2.1730558639	1.1765346289	1.2895464746
C	-3.0042481306	1.8196271388	0.2796542653
C	-2.330014902	1.6255637522	-1.0574556443
C	-0.9087534129	1.1760925656	-0.7003583377
H	-2.8532863609	0.8244449465	-1.5805564454
H	-2.3939227122	2.5387594856	-1.6459110254
O	-3.9982653077	2.3492961818	0.5956070049

O	-0.3638105382	-0.1328361413	1.2911671453
O	-0.3790012116	0.0911466372	-1.4240586995
H	-0.1737872391	0.3117845757	-2.3417493178
C	0.0498431183	2.3838694136	-0.6897171451
H	-0.3263950532	3.1783923919	-0.0398865195
H	0.0685508413	2.8203328916	-1.6927929369
C	1.4856041084	2.119922794	-0.3003869586
O	1.9759455998	1.0487852327	0.0218041495
O	2.2006309943	3.222542768	-0.3523304
H	3.126209512	3.038217145	-0.1109157722
Zn	1.2313825267	-0.8825636692	0.1793049609
Cl	1.761712479	-2.5231141379	-1.1041613801
O	2.2175606656	-1.3263040586	1.9868627815
H	2.732501701	-2.1438915782	1.9482020198
H	1.8026538204	-1.2725832324	2.8568315419
S	-3.0101762202	-1.7658604984	-0.3878791952
C	-1.8450374616	-3.0165995186	0.2389902228
H	-3.9311324425	-1.8677063324	0.5845952721
H	-1.0346113818	-3.0775293547	-0.4845512879
H	-2.3333355016	-3.9852553607	0.304596906
H	-1.4488359878	-2.7288693589	1.2110187774
H	-1.4488359878	-2.7288693589	1.2110187774

SI DFT 019
E(RB3LYP) = -3438.71247230 A.U.
First frequency = -176.5746 cm^{-1}

C	-0.5987469098	-0.3231070505	0.7545101811
O	-1.6120550423	-0.1785136948	1.4590292264
C	-3.1792811349	0.610326705	-0.5554015659
C	-1.9841196893	0.139418579	-1.3282430938
C	-0.6223861654	0.4118682614	-0.6255931235
H	-2.0330549525	-0.9221705746	-1.5507913875
H	-2.0092850281	0.6869372935	-2.2736185999
O	-3.5831133969	1.7009578308	-0.4028085239
O	0.4324634093	-0.969295662	1.059218507
O	0.3857967418	-0.1852388619	-1.419791931
H	0.6331863005	0.3467041571	-2.1881807067

C	-0.3819418119	1.9124228927	-0.4251973489
H	-1.150768376	2.343177358	0.2167179511
H	-0.4652376862	2.4396118525	-1.38126433
C	0.96252958	2.2748090025	0.1640579254
O	1.8796566771	1.5128398363	0.40953685
O	1.0503857788	3.574161171	0.3850323435
H	1.9289550527	3.7864516418	0.7463160077
Zn	2.1413231497	-0.6060578893	0.099023584
Cl	3.5945924756	-1.1368563292	-1.4087483005
O	3.2023966715	-0.913590479	1.9085020741
H	4.0755588222	-1.3079221634	1.7823883786
H	2.7710237609	-1.3591081494	2.6481479224
S	-4.234501822	-0.7548442678	0.4208103807
C	-3.8071252855	-2.3877619895	-0.2484776368
H	-3.1787569897	-0.6603013641	1.3232113517
H	-4.1856793489	-2.4529630186	-1.265649935
H	-4.3213436619	-3.1163674137	0.3764970667
H	-2.7348411186	-2.5549206739	-0.2059902669
H	-2.7348411186	-2.5549206739	-0.2059902669

SI DFT 020
E(RB3LYP) = -3438.71115416 A.U.
First frequency = -153.0125 cm^{-1}

C	-0.5067765347	-0.5518411103	0.7459115645
O	-1.5861085086	-0.5009414466	1.3527127738
C	-3.0051177062	-1.0615516658	-0.6470986099
C	-1.7759882176	-0.7734163958	-1.4593177667
C	-0.5547366606	-0.1173063292	-0.758341224
H	-1.4492453698	-1.7482598055	-1.8220045105
H	-2.0912925592	-0.1756741434	-2.3194158744
O	-3.5507604951	-2.0728232307	-0.4415876065
O	0.6048959894	-0.8862513512	1.2220732837
O	0.6183605484	-0.6293802396	-1.3658935951
H	0.8205278734	-0.2282388418	-2.2219301167
C	-0.5903483459	1.4146835837	-0.8577646316
H	-1.4869810764	1.8248885776	-0.3930448666
H	-0.6417846009	1.7153759699	-1.9098058538

C	0.5960619457	2.141158756	-0.2564942224
O	1.6215634065	1.6413145616	0.1583782591
O	0.3913928075	3.4492693153	-0.2491549223
H	1.1759269749	3.8993239353	0.1108452787
Zn	2.2870529628	-0.4405311321	0.2638543546
Cl	3.9294534788	-0.851609813	-1.0777668646
O	3.2429384506	-0.2808495433	2.1503822593
H	4.1771174127	-0.5276571294	2.1371691945
H	2.8276794912	-0.6999226216	2.9146822758
S	-3.9633741618	0.5938621771	0.0248937367
C	-5.4016548986	-0.1891705593	0.7914217764
H	-3.0548494147	0.4968227793	1.046980772
H	-6.2025639985	-0.185442717	0.0568290805
H	-5.6776659087	0.4110790738	1.6552576319
H	-5.1398888843	-1.2043796539	1.0807584236
H	-5.1398888843	-1.2043796539	1.0807584236

SI DFT 021
E(RB3LYP) = -3438.74314986 A.U.
First frequency = 31.5652 cm^{-1}

C	-0.5057323616	-0.5386683385	0.8839572787
O	-1.4999211867	-0.4152971569	1.7179086386
C	-3.2518332111	-0.9325614425	-0.6952204564
C	-1.8563337869	-0.8286855975	-1.3076639574
C	-0.6888989115	-0.1046674588	-0.5938247504
H	-1.5388396501	-1.8584753838	-1.4669608989
H	-1.9760723499	-0.3666874226	-2.2927397757
O	-3.8560235406	-1.9648288968	-0.6991978739
O	0.573680072	-0.9450509036	1.2824927111
O	0.5124477727	-0.5608120779	-1.2076224199
H	0.5644586979	-0.3438176693	-2.1480502912
C	-0.7589109922	1.4311879205	-0.6789616796
H	-1.6414322094	1.8291570346	-0.1773334289
H	-0.8662982303	1.7231206497	-1.7276033523
C	0.4481339574	2.1617565078	-0.1306197588
O	1.4680967095	1.6553788997	0.3053964253
O	0.2736178867	3.4671224756	-0.1731751527

H	1.067495047	3.921379263	0.1614863293
Zn	2.2592495883	-0.33158592	0.1965994974
Cl	3.8305809643	-0.7096688583	-1.2293347148
O	3.246705654	-0.3096653989	2.0487460985
H	4.1899980683	-0.5112777924	1.9811826838
H	2.8932568174	-0.7643007009	2.823804237
S	-3.9405004178	0.5741087212	-0.0000447828
C	-5.5174899721	-0.0604773719	0.6295601012
H	-2.3301414715	-0.0523134602	1.3154161909
H	-6.1367592388	-0.366712629	-0.2089254134
H	-5.9832320958	0.7638969628	1.1646526255
H	-5.341085609	-0.9005249549	1.2951918899
H	-5.341085609	-0.9005249549	1.2951918899

SI DFT 022
E(RB3LYP) = -3438.73838058 A.U.
First frequency = 28.9598 cm^{-1}

C	-0.6103531329	-0.5138352026	0.8362498986
O	-1.615416508	-0.5978322334	1.6646066234
C	-3.3080471046	0.5514106388	-0.6737374891
C	-2.0599603207	-0.0864266049	-1.2697472901
C	-0.7513891597	0.2433074237	-0.5098053573
H	-2.1317544434	-1.1658857153	-1.3697950562
H	-1.9594137109	0.3370586244	-2.2709958409
O	-3.4879634013	1.7389290879	-0.7279346944
O	0.4649839827	-0.9914210912	1.1612785508
O	0.3333699753	-0.2768767576	-1.2683399056
H	0.4199508473	0.144974991	-2.1338536956
C	-0.5834229247	1.7566742363	-0.2659956618
H	-1.35876446	2.1426668927	0.3955956973
H	-0.7460052721	2.287451266	-1.2079557552
C	0.7527699523	2.2097496531	0.2749626205
O	1.7396024877	1.5213684605	0.4834612877
O	0.7495000826	3.5057351264	0.5071198786
H	1.622167184	3.7896919978	0.8330226487
Zn	2.1780507757	-0.490692234	0.0712509244
Cl	3.5654352765	-1.0140391729	-1.4930299042

O	3.2689182005	-0.8667765145	1.831876568
H	4.1532863101	-1.2151309193	1.653932378
H	2.902702847	-1.3429568638	2.5875232107
S	-4.4620956591	-0.4337454262	0.2725763289
C	-4.1497516154	-2.1754182776	-0.1637939926
H	-2.5037107825	-0.3351507565	1.3192085107
H	-4.2655443613	-2.328864386	-1.23365766
H	-4.9293983383	-2.7312669587	0.3547503921
H	-3.1824897268	-2.5286292841	0.1850107846
H	-3.1824897268	-2.5286292841	0.1850107846

SI DFT 023
E(RB3LYP) = -3438.76654153 A.U.
First frequency = 27.8679 cm^{-1}

C	-0.41843	-0.61975	-1.35469
O	-1.21717	-1.2549	-2.18567
C	-2.83601	-0.02106	0.39145
C	-2.02081	-1.30299	0.49878
C	-0.5517	-1.06779	0.11351
H	-2.04424	-1.61752	1.54527
H	-2.45856	-2.10142	-0.0976
O	-2.31417	1.06571	0.59754
O	0.38146	0.22846	-1.70705
O	0.02513	-0.05474	0.91886
H	-0.66318	0.65114	1.01785
C	0.26235	-2.35112	0.33619
H	-0.06647	-3.14762	-0.3337
H	0.07096	-2.7038	1.35229
C	1.75997	-2.21243	0.20489
O	2.38408	-1.18981	-0.03668
O	2.36649	-3.36622	0.38812
H	3.33153	-3.24999	0.32778
Zn	1.77514	0.80367	-0.1492
Cl	2.1757	2.56652	1.03061
O	3.18534	1.20064	-1.70652
H	2.93668	1.28131	-2.63548
H	3.63004	2.02279	-1.45203

S	-4.50883	-0.24999	-0.00658
C	-5.11366	1.45345	0.11281
H	-1.07845	-0.94264	-3.09747
H	-4.9586	1.83059	1.12018
H	-6.17656	1.41068	-0.11412
H	-4.59893	2.08226	-0.60847
H	-4.59893	2.08226	-0.60847

13.2 C_b selectivity

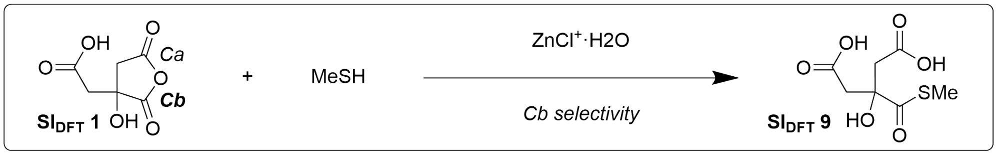

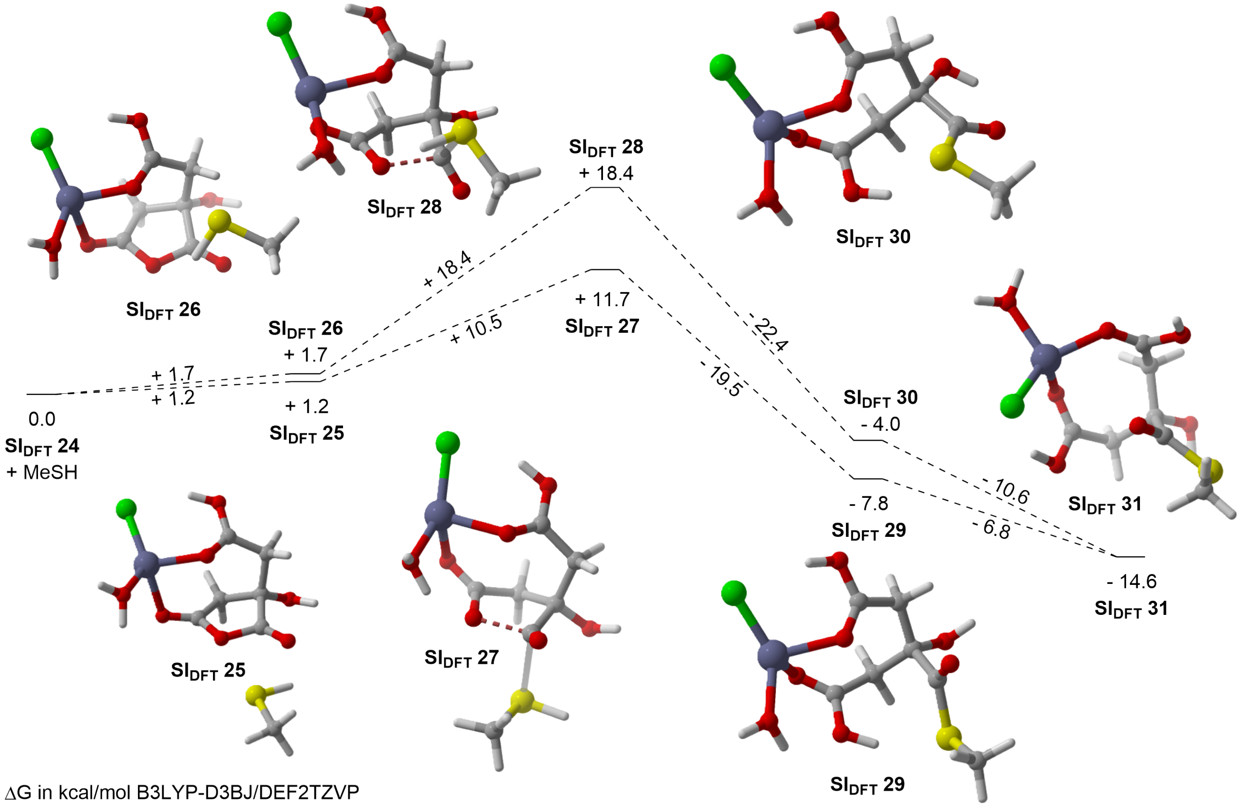

Figure S31: Nucleophilic addition of MeSH to the C_b site of citric anhydride with $ZnCl^+H_2O$ as Lewis acid. The Gibbs free energies relative to the starting materials are given in kcal/mol.

SI DFT 024
E(RB3LYP) = -2999.95291343 A.U.
First frequency = 31.4317 cm^{-1}

Zn	-1.5785	0.17215	0.00038
Cl	-2.38827	0.60052	-1.9536
O	-2.50651	0.29807	1.77781
O	0.19176	1.26588	0.05371
O	0.54588	1.77708	-2.09885
O	-0.40275	-1.43793	0.63149
O	3.26699	0.43438	2.24744
O	1.37868	-0.74422	1.76948
O	4.06454	-0.51843	-0.4188
C	2.43192	1.25071	-0.7615
C	0.79317	-1.19092	0.6537
C	1.77198	-1.23296	-0.48019
C	0.95193	1.46428	-0.90524
C	2.76592	-0.12314	-0.09918
C	2.58651	-0.06345	1.43053
H	-3.39642	0.6504	1.91145
H	-2.29756	-0.31854	2.49259
H	2.85413	2.05996	-0.16045
H	2.9044	1.26427	-1.74148
H	-0.4421	1.74387	-2.16634
H	4.71216	0.0557	0.01527
H	1.28792	-1.13623	-1.44916
H	2.29616	-2.19257	-0.45388
H	2.29616	-2.19257	-0.45388

SI DFT 025
E(RB3LYP) = -3438.72326630 A.U.
First frequency = 22.9500 cm^{-1}

Zn	2.2277591476	-0.8121233813	0.0725584071
Cl	3.8201425604	0.3880246615	0.9045330828
S	-4.1815301651	-0.7283578406	1.273136934
O	-2.5546445862	2.2722287372	0.2907153596
O	-1.3025682055	-0.9153573576	-0.497538138

O	-2.74599095	0.2244553687	-1.845089122
O	0.3919337319	-1.3460505869	0.872127616
O	1.8340979784	2.5128930473	-0.3387672412
O	1.1543229874	0.5219411841	-1.1083920391
O	2.4334733748	-2.5736797146	-0.8813864452
C	-4.9762611015	-1.6431532124	-0.087660315
C	-2.0051883712	0.2508674707	-0.9320795747
C	-1.5037043153	1.4454175803	-0.0997383505
C	0.9338626473	1.7066209486	-0.8204144359
C	-0.8921900415	0.7281111069	1.111548396
C	-0.5262251851	-0.6069868493	0.5391118166
C	-0.4512463751	2.2783797466	-0.9143354799
H	-4.80730317	-1.1536460445	-1.043919946
H	-4.5145978571	-2.6284132025	-0.1014470602
H	-6.0398821059	-1.7552682412	0.1049854839
H	-4.8946190599	0.4046200298	1.179484704
H	-1.6822589761	0.5683938094	1.8498081682
H	-0.0509974068	1.2200644569	1.5941329388
H	-3.0920279356	2.5123786996	-0.4774245718
H	2.6814166217	2.0366170348	-0.1474852982
H	-0.4592604158	3.2849869805	-0.5029867956
H	-0.7512203336	2.32438399	-1.963538199
H	1.7601316789	-3.2613979063	-0.7913925817
H	3.2820138286	-2.9853155158	-1.0916513131
H	3.2820138286	-2.9853155158	-1.0916513131

SI DFT 026
E(RB3LYP) = -3438.72105872 A.U.
First frequency = 9.2304 cm^{-1}

Zn	2.0993909825	-0.4686291894	0.4211604038
Cl	3.5895140262	0.6339153871	-0.6818285022
S	-2.3170967099	-1.9769383079	-1.6736936898
O	-2.5511777959	2.7704598149	0.5914618995
O	-1.2658670103	-0.2288723237	1.8303027211
O	-3.3846864867	0.0131738308	1.0309999584
O	0.9322855232	0.0094499948	2.0863529785
O	0.8243211758	1.4917934978	-2.0639182977

O	0.3495894459	-0.1615899254	-0.6308838431
O	2.2435304969	-2.3826616701	1.0363566237
C	-4.1240489849	-2.2166961493	-1.6089755463
C	-2.2873417512	0.4280361578	1.0754161866
C	-1.6772470953	1.6933696983	0.4422729657
C	0.0236069515	0.8549545637	-1.259996786
C	-0.4403677584	1.896666767	1.3325508004
C	-0.1486848329	0.5030771556	1.8011704815
C	-1.3251693718	1.4766758533	-1.0649943445
H	-4.3649698526	-3.2691471743	-1.4848619664
H	-4.5655873767	-1.6259200642	-0.8103326202
H	-4.5097770793	-1.8771864957	-2.5682422395
H	-2.0415763427	-2.4172807474	-0.4355437974
H	-0.7314188857	2.4983953019	2.1984811776
H	0.4198581797	2.3695076868	0.8649718059
H	-3.4233990289	2.5492121715	0.2347685438
H	1.738926606	1.1218553674	-2.0273271539
H	-1.356994948	2.451386504	-1.5464959497
H	-2.0627866264	0.8112201139	-1.5239146623
H	1.8802769019	-2.6448392133	1.8931316034
H	2.978989648	-2.9679916061	0.8115922493
H	2.978989648	-2.9679916061	0.8115922493

SI DFT 027
E(RB3LYP) = -3438.71291451 A.U.
First frequency = -177.7034 cm^{-1}

Zn	-2.4407383782	0.0067816885	0.0141606729
Cl	-3.9089475753	1.5620507302	-0.2464717102
S	3.4767554707	-1.4383965802	-0.4842705806
O	3.1205117516	1.5269955551	-0.6332192547
O	0.56095109	-1.0970360793	-0.046156086
O	2.0297678632	-0.2739199733	1.8052036649
O	-1.0724856756	-0.4497284892	-1.4147604407
O	-1.0788428582	3.0849982328	0.0723714906
O	-0.8757229048	1.0859463958	1.0571553443
O	-2.0424596274	-1.670611258	1.0664583173
C	3.4059277354	-2.8006656684	0.7047167311

C	1.914936586	-0.1340114032	0.6515223389
C	1.8307133516	1.1245874378	-0.266418002
C	-0.3809683984	2.1061925884	0.5851774715
C	1.0727355048	0.6585026116	-1.5106711982
C	0.1009199079	-0.3815457079	-1.0092174383
C	1.1143809186	2.2824691954	0.490243947
H	3.2143094685	-2.4097110301	1.702452151
H	2.5909198557	-3.4465803511	0.3873293011
H	4.3422659874	-3.351600959	0.6747161853
H	4.55218036	-0.8034731811	0.0120931505
H	1.7776235768	0.200785505	-2.2063136454
H	0.5592622741	1.4651280523	-2.0277706244
H	3.5339000168	2.0407000223	0.073616511
H	-2.0460454839	2.8678115496	0.0853942099
H	1.3364413801	3.2028613623	-0.0462704179
H	1.5131648527	2.3534146545	1.5048280447
H	-1.083048509	-1.8295477708	0.9811224462
H	-2.3010666655	-1.7902416884	1.9893595258
H	-2.3010666655	-1.7902416884	1.9893595258

SI DFT 028
E(RB3LYP) = -3438.70167028 A.U.
First frequency = -178.0105 cm^{-1}

Zn	-2.1232710703	0.5718594533	0.4275080963
Cl	-3.8471101948	-0.2568142804	-0.5608347875
S	2.0509883334	1.4313135299	-1.3105110289
O	2.8169521436	-2.4776830886	0.2220605891
O	0.8745386859	0.5135337228	1.4366258312
O	3.3709903295	0.1542140249	1.0226360954
O	-1.0918666199	-0.4565677367	1.8344456752
O	-1.2725226313	-1.942116368	-1.5727777922
O	-0.5536624855	0.0872258255	-0.9730161837
O	-1.2147408419	2.3312381596	0.9177597146
C	3.5523115175	2.3754810575	-0.9538356908
C	2.368308664	-0.149842223	0.5299861196
C	1.7731775109	-1.5596473239	0.1567122391
C	-0.3346009187	-1.0890889703	-1.2615014062

C	0.8326809391	-1.8241549195	1.3419773314
C	0.1327729596	-0.5140175838	1.5997317827
C	1.0545871675	-1.6766288019	-1.2138903589
H	3.3806928034	3.4247168343	-1.1802456013
H	3.843641139	2.2422915622	0.086702887
H	4.3306861811	1.9887504858	-1.6075809174
H	1.1733902546	2.1630773722	-0.6118561042
H	1.4378341717	-2.0986340895	2.2081198285
H	0.1270113688	-2.6255982337	1.1379392113
H	3.4051890101	-2.4077655009	-0.5418088314
H	-2.1675671293	-1.5195776677	-1.5127957595
H	0.9923826713	-2.741487465	-1.4279067888
H	1.6517528682	-1.2033871914	-1.9946299194
H	-0.4121709953	2.069315318	1.4113695504
H	-1.651293832	3.0595060981	1.3788282187
H	-1.651293832	3.0595060981	1.3788282187

SI DFT 029
E(RB3LYP) = -3438.74548643 A.U.
First frequency = 36.9518 cm^{-1}

Zn	2.264831857	-0.6467797414	0.0294055617
Cl	4.0386462593	0.4029312925	-0.59141622
S	-3.4517940615	-0.8065510207	0.2270015537
O	-2.6814796152	2.2326884053	0.935053402
O	-0.5955918807	-1.6104222912	1.1406500747
O	-2.3083761835	0.4992752186	-1.7654732295
O	1.0706690079	-0.2843259674	1.659842123
O	1.5505721004	2.3524964786	-1.1507739512
O	0.6568746742	0.313775416	-0.9048833271
O	1.8257855529	-2.6099583465	-0.1648208506
C	-4.0398841019	-1.7038029552	-1.2337671451
C	-2.3945353321	0.4014496225	-0.5745805043
C	-1.6813378718	1.3834972871	0.3947445688
C	0.5716371275	1.5447260597	-0.852073651
C	-1.1095921191	0.7118726524	1.6774789831
C	-0.1400786364	-0.4240033941	1.4904623763
C	-0.6676189452	2.2428104233	-0.3787603941

H	-4.666754532	-1.0449258782	-1.8283520926
H	-3.1996936335	-2.0556022025	-1.8257238412
H	-4.6253555648	-2.5405905114	-0.8594993796
H	-1.5702023507	-1.5891372712	0.9389669061
H	-1.9654968318	0.3689358103	2.2604246449
H	-0.6060118178	1.4858426325	2.2529183574
H	-3.0819133173	2.7694589673	0.2378221697
H	2.3878566266	1.8594123127	-1.327252403
H	-0.368112294	3.0815286987	0.250397303
H	-1.1569947494	2.6632211287	-1.2613735343
H	1.0790630687	-2.9440976404	0.352828425
H	2.4588009812	-3.319721953	-0.3306271747
H	2.4588009812	-3.319721953	-0.3306271747

SI DFT 030
E(RB3LYP) = -3438.73870917 A.U.
First frequency = 32.3991 cm^{-1}

Zn	2.2154554395	-0.396255857	0.3594740942
Cl	3.8509296184	0.4487730708	-0.7581901676
S	-2.378016427	-1.2738576759	-0.7830871352
O	-2.6970260488	2.6233673021	0.1884272576
O	-0.5353309857	-1.0928806942	1.7178324282
O	-4.1423444691	0.5283540626	-0.0824402712
O	0.9966990799	0.4684140635	1.7597426768
O	1.1898913755	1.7255253975	-1.9715322269
O	0.4952626805	-0.0592721221	-0.8154527601
O	2.0282773483	-2.3150459405	0.9703743317
C	-3.9586539729	-2.1563959479	-0.8983973089
C	-2.9703499452	0.3093307391	-0.2550716944
C	-1.9470999901	1.4515713204	0.0210967459
C	0.2883917657	1.0534078733	-1.3129567729
C	-1.2419290214	1.2388934053	1.4181685733
C	-0.1940652368	0.1826405544	1.6244221373
C	-1.0170449282	1.7733255156	-1.1750565424
H	-3.7087101654	-3.1774237158	-1.1786729684
H	-4.4746610934	-2.1365144919	0.0570864812
H	-4.5738254017	-1.6953699299	-1.6662001649

H	-1.4040083305	-1.2814958476	1.2979998917
H	-2.0499653932	1.0765211531	2.1345879527
H	-0.7748574108	2.1928093724	1.6531687501
H	-3.6318272101	2.3721976274	0.2889027514
H	2.072575555	1.2814772306	-1.938950638
H	-0.7992125457	2.8393893778	-1.1179082338
H	-1.5754343225	1.6402355184	-2.1060878094
H	1.3231725348	-2.5098985391	1.6037108593
H	2.7512081805	-2.9464440094	1.0754604932
H	2.7512081805	-2.9464440094	1.0754604932

SI DFT 031
E(RB3LYP) = -3438.75600913 A.U.
First frequency = 27.6744 cm^{-1}

Zn	2.20186	-0.57628	-0.104
Cl	2.58476	-0.47202	2.03282
S	-3.27942	1.54596	0.48444
O	-3.09661	-0.35259	-1.67792
O	-0.08766	2.15845	-2.08097
O	-0.70214	0.99001	0.59724
O	1.43837	0.69235	-1.45013
O	-0.31235	-1.75806	1.67212
O	0.68786	-1.89624	-0.33076
O	3.85026	-1.19314	-1.15811
C	-2.71941	2.55334	1.88241
C	-1.77949	0.76451	0.08072
C	-1.86815	-0.33421	-0.99704
C	-0.32778	-1.80908	0.37229
C	-0.79897	-0.08167	-2.10839
C	0.27942	0.92862	-1.80707
C	-1.67392	-1.70465	-0.27085
H	-1.96782	3.26307	1.54753
H	-3.59894	3.08085	2.24437
H	-2.31232	1.91609	2.66285
H	0.63058	2.77812	-1.86246
H	-1.35624	0.29989	-2.96198
H	-0.32108	-1.01686	-2.38305

H	-3.81008	-0.64853	-1.09733
H	0.60767	-1.62874	2.01338
H	-1.76522	-2.48332	-1.02775
H	-2.45854	-1.82519	0.47481
H	4.10446	-0.90661	-2.04385
H	4.64738	-1.44221	-0.67193
H	4.64738	-1.44221	-0.67193

14 Catalyst choice

14.1 Lewis acid model choice

As the reaction mixture is not totally dry, we proceeded to model the Lewis acid $ZnCl_2$ by $Zn^+Cl{\cdot}L$, with L either being H_2O or MeSH. Both starting complexes were modelled and it was found that they exhibited similar properties with respect to the bound length and lowest antibonding orbital (Fig S32). A decision was taken to utilise $Zn^+Cl{\cdot}H_2O$ as the Lewis acid.

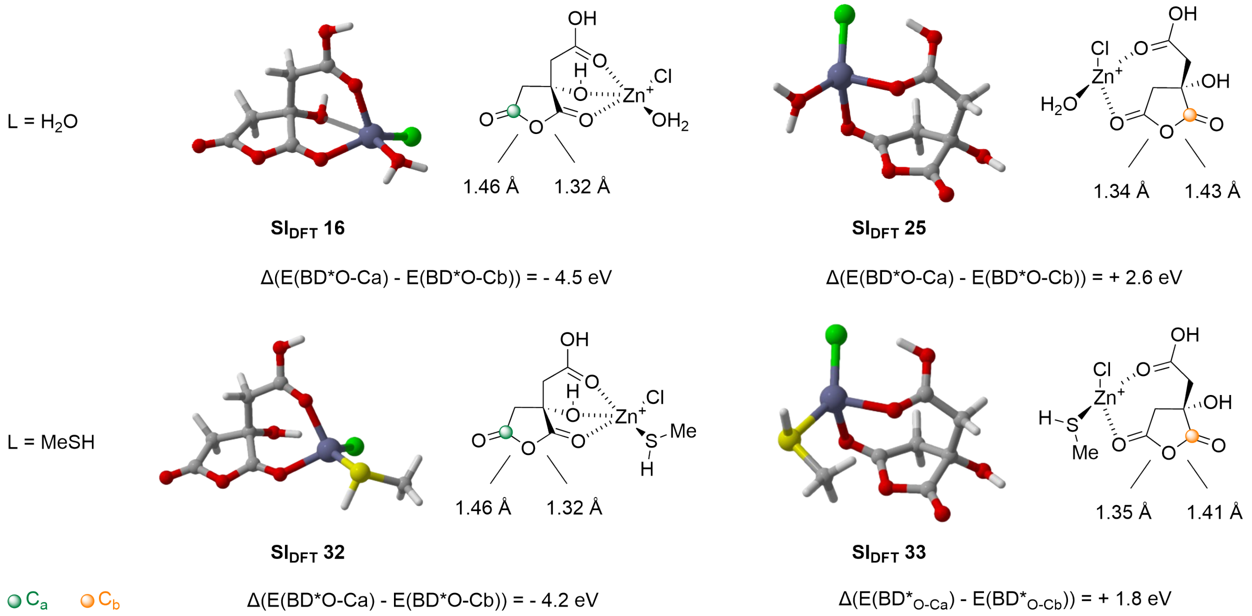

Figure S32: SI_{DFT} 1 complex with $Zn^+Cl{\cdot}L$, L being either H_2O or MeSH.

SI DFT 032
E(RB3LYP) = -3362.26322337 A.U.
First frequency = 21.1621 cm^{-1}

C	-1.981397891	0.9894364857	-0.0614522336
O	-3.1123506495	1.6037145702	-0.3405315981
C	-4.2584433948	0.8225339904	0.109698714
C	-3.7184332689	-0.3527161643	0.868693674
C	-2.2144450894	-0.4127342296	0.553469774

H	-3.8448157572	-0.1603506349	1.936121367
H	-4.2784448319	-1.2517867762	0.6210781966
O	-5.3411193636	1.1849746033	-0.1461881909
O	-0.9145220864	1.5345999231	-0.2920313476
O	-1.5304355469	-0.6008187504	1.7531344805
H	-0.5689235687	-0.4234428576	1.7207059883
C	-1.9624627797	-1.5385715306	-0.4751843423
H	-2.5765685507	-1.3867095114	-1.3705818347
H	-2.3105893102	-2.4678960285	-0.0237376619
C	-0.5636665883	-1.78536087	-0.9771600714
O	0.3602568378	-0.9825502762	-1.0409770687
O	-0.4281793805	-3.013553688	-1.4238280377
H	0.4678653745	-3.1493170489	-1.7799469588
Zn	0.9639289141	0.7600144534	-0.1331720345
Cl	1.5520454239	0.2357222114	1.891746572
S	2.3540663307	2.0615559068	-1.5404846224
H	1.6776274771	3.2147067951	-1.3789987115
C	3.761230815	2.3980828547	-0.4167671199
H	3.4121256368	2.6265701913	0.5850022285
H	4.3560258907	1.4879807091	-0.4040000464
H	4.3407712737	3.2138969526	-0.8392238289
H	4.3407712737	3.2138969526	-0.8392238289

SI DFT 033
E(RB3LYP) = -3362.25489615 A.U.
First frequency = 22.8072 cm^{-1}

Zn	-1.5501957385	-0.3511267373	0.1727355558
Cl	-2.0623147646	-2.4582134645	0.2677299471
S	-2.7525219608	1.6280005107	-0.2403431817
O	0.1381059447	-0.3614475735	-1.0880835571
O	0.7252218397	-2.5245689428	-1.0779893175
O	-0.1938390138	0.3747569953	1.6971770465
O	2.7680234636	2.2798219114	-0.9681506891
O	1.2142264069	1.6924751654	0.5785107539
O	4.2296485228	0.0135502704	0.2227483995
C	2.4582342187	-0.9112389053	-1.1643333116
C	0.9283884928	0.575218351	1.2836887117

C	2.122749625	-0.3289035377	1.3289564898
C	0.995872894	-1.2568493852	-1.1378219358
C	2.8505476901	0.0259020515	0.02088125
C	2.3502986136	1.4602014094	-0.2341472603
H	-3.5148538533	1.156101991	-1.245296736
H	2.6948829539	-0.4155676195	-2.108541823
H	3.0574974875	-1.8150351989	-1.079921764
H	-0.2389261724	-2.6829081184	-0.8851640257
H	4.685305496	0.4446391179	-0.5146266201
H	1.8389231331	-1.3711975445	1.4525764671
H	2.7573907818	-0.0399172992	2.171132319
C	-1.5864244582	2.6417858243	-1.2224787206
H	-1.1089299535	2.0485719698	-1.9952753487
H	-2.1455050185	3.4716928255	-1.6455119449
H	-0.845944716	3.0125066545	-0.5187366759
H	-0.845944716	3.0125066545	-0.5187366759

14.2 Silica[1]

Silica can be a potential catalyst through hydrogen bonding for the reaction. Transition states were modelled, without the involvement of a Lewis acid, taking account of a silica model for the nucleophile attack of MeSH on citric anhydride.[8] It was found that the two lowest TS for C_a and C_b selectivity exhibited similar $\Delta\Delta G^{\ddagger}$ values (1.2 kcal/mol in the case of silica as catalyst and 1.0 kcal/mol in the case of no catalyst, Figure S33 and S34). Consequently, it was determined that silica should not be included in the reaction model, as this would result in a reduction in the computational resources required.

MeSH
E(RB3LYP) = -438.567091522 A.U.
First frequency = 254.6725 cm^{-1}

C	0.048118000	1.159488000	0.000000000
S	0.048118000	-0.670123000	0.000000000
H	-0.433540000	1.565426000	0.901008000
H	-0.433540000	1.565426000	-0.901008000
H	1.103955000	1.465028000	0.000000000
H	-1.295469000	-0.830845000	0.000000000

SI DFT 001
E(RB3LYP) = -683.154767080 A.U.
First frequency = 52.1719 cm^{-1}

O	3.219599000	0.313302000	-0.952495000
O	2.504088000	-0.236856000	1.094572000
O	-0.074113000	-1.025397000	1.322336000
O	0.272155000	1.896177000	0.177129000
O	-3.684313000	-0.153435000	-0.147226000
O	-1.852403000	1.137753000	0.115737000
C	2.328759000	-0.200770000	-0.105591000
C	1.111182000	-0.773313000	-0.800005000

[1]All calculations were made at the B3LYP-D3BJ/DEF2SVP level of theory and FAST-CAR was applied to every TSs.

C	-0.166020000	-0.528428000	0.008972000
C	-1.453693000	-1.077216000	-0.595323000
C	-2.501718000	-0.057547000	-0.189531000
C	-0.489511000	0.981449000	0.096631000
H	3.965790000	0.647380000	-0.422761000
H	1.264643000	-1.862430000	-0.881878000
H	1.033126000	-0.362583000	-1.815564000
H	0.804617000	-0.757530000	1.662064000
H	-1.417829000	-1.106336000	-1.696450000
H	-1.704453000	-2.075896000	-0.216747000

Silica model
E(RB3LYP) = -7037.33387011 A.U.
First frequency = 18.4199 cm^{-1}

O	-5.424690000	-2.789498000	0.261855000
O	-2.787525000	-2.377846000	0.194824000
Si	-4.092574000	-2.575098000	1.207393000
H	-3.882721000	-3.741572000	2.071076000
O	-4.275610000	-1.214265000	2.124551000
Si	-1.941880000	-1.162399000	-0.558093000
O	-5.583499000	-0.862411000	-1.656478000
O	-2.975334000	-0.248215000	-1.447658000
Si	-4.522048000	0.331358000	-1.257445000
H	-4.725604000	1.500350000	-2.115823000
O	-4.752915000	0.715876000	0.334920000
Si	-6.041895000	-2.403001000	-1.235228000
H	-5.504613000	-3.350967000	-2.241458000
H	-7.517564000	-2.454121000	-1.147359000
H	3.918001000	5.598120000	-0.582273000
H	5.541033000	-0.150985000	2.281585000
O	-0.283707000	1.760377000	2.118842000
O	0.542479000	-2.899027000	0.575835000
O	1.810819000	2.549114000	0.600853000
O	1.931735000	-0.617372000	0.345906000
Si	0.925499000	2.880030000	1.954871000
Si	1.524258000	-1.842822000	1.382350000
H	1.775417000	2.842541000	3.147271000

H	0.879461000	-1.329549000	2.591910000
O	0.237822000	4.371478000	1.743614000
O	2.915923000	-2.645607000	1.784378000
Si	-1.583683000	1.255466000	1.267721000
Si	0.079821000	-3.207231000	-0.981362000
O	-1.245496000	-0.209586000	0.589698000
O	-0.801332000	-1.882994000	-1.473442000
H	6.989655000	-0.408259000	0.310754000
H	2.481268000	6.236292000	-2.472782000
Si	-4.379500000	0.425903000	1.919928000
O	-2.899795000	1.099088000	2.238433000
O	-0.827044000	-4.568129000	-1.053455000
H	-5.387888000	0.995453000	2.817233000
Si	2.603058000	5.422997000	-1.241744000
Si	5.658805000	-0.673530000	0.898574000
Si	1.745746000	2.461372000	-1.047684000
Si	3.066729000	-0.207507000	-0.777262000
O	2.400354000	3.827185000	-1.666982000
O	4.502083000	0.066630000	-0.038601000
O	2.577176000	1.149973000	-1.550455000
O	1.381602000	-3.390175000	-1.952658000
O	-1.966831000	2.324243000	0.074914000
O	3.230620000	-1.432873000	-1.859497000
O	0.171361000	2.339970000	-1.517779000
Si	3.002363000	-3.071220000	-1.921974000
Si	-1.246918000	3.124329000	-1.178313000
H	-2.132085000	3.153348000	-2.345019000
H	3.632646000	-3.623763000	-3.123061000
Si	-0.022109000	5.433075000	0.496347000
Si	4.162303000	-3.375382000	0.974337000
O	1.424060000	5.872603000	-0.159158000
O	5.409989000	-2.315097000	0.890051000
H	-0.737220000	6.612681000	0.992373000
O	4.696196000	-4.714922000	1.742366000
O	3.636885000	-3.740833000	-0.549838000
O	-0.905139000	4.663019000	-0.679635000
H	4.124269000	-5.490596000	1.726180000
H	-1.707214000	-4.463402000	-0.667639000

14.2.1 C_a selectivity

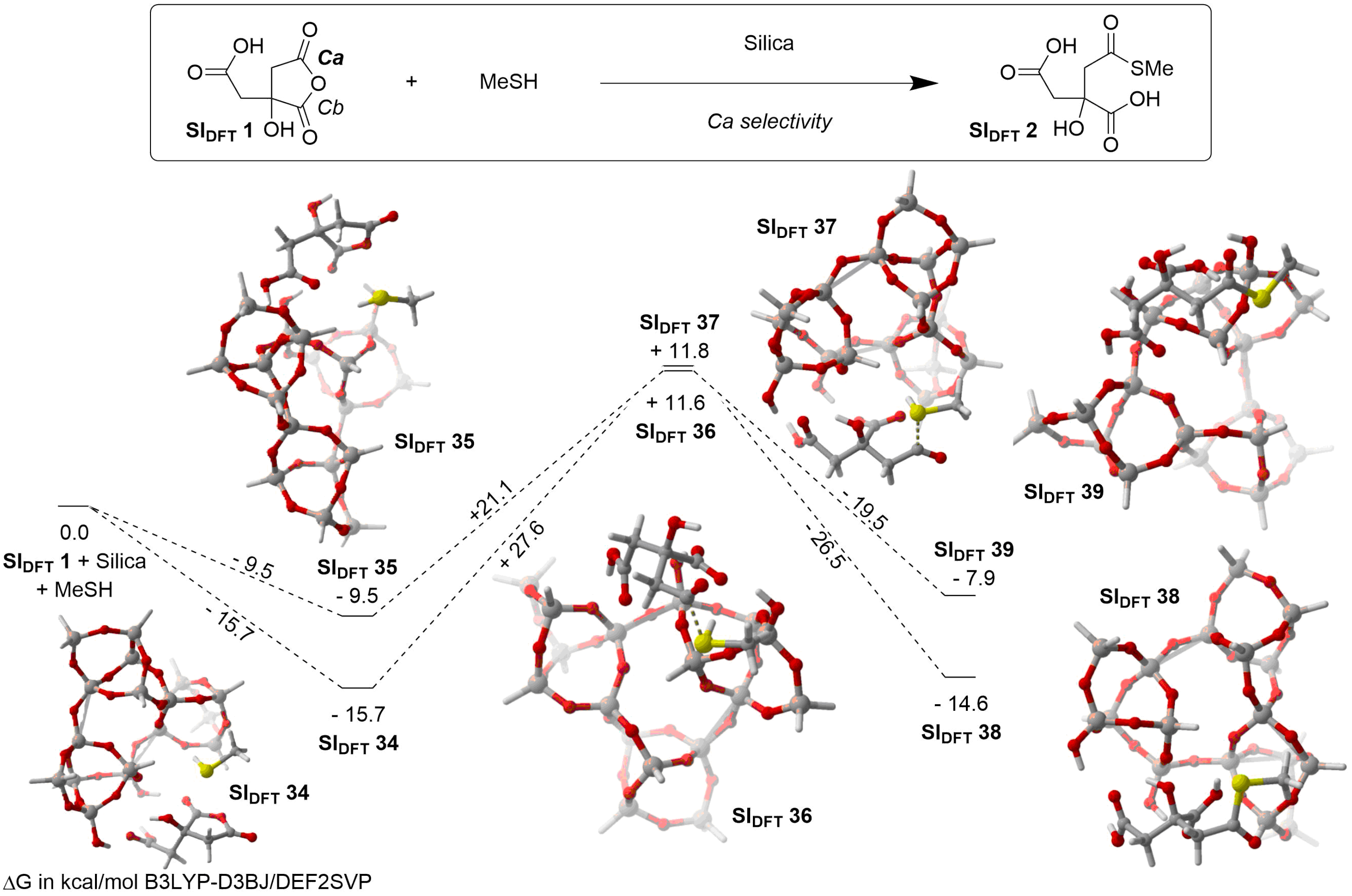

Figure S33: Nucleophilic addition of MeSH to the C_a site of citric anhydride with a model of silica as catalyst. The Gibbs free energies relative to the starting materials are given in kcal/mol.

SI DFT 034
E(RB3LYP) = -8159.12599230 A.U.
First frequency = 13.6527 cm^{-1}

O	1.9001692966	4.9361956593	-2.7683001875
O	0.3615303993	2.8079800902	-2.2004243079

Si	0.4728252041	4.4618924711	-2.100844383
H	-0.6505117532	5.0841391208	-2.8055011118
O	0.4713458572	4.8965229518	-0.5036273369
Si	0.919579085	1.5128627418	-1.3602570004
O	3.942597173	3.9838452843	-1.2405867638
O	2.4182705064	1.8391392697	-0.7622717984
Si	3.3873567137	2.982840074	-0.0555884681
H	4.4996725686	2.3781823923	0.6742662395
O	2.4422368236	3.8580618933	0.993200062
Si	3.5385360487	4.6581718475	-2.7048837457
H	3.9064453996	3.7188959793	-3.7919560995
H	4.2383499836	5.9561453821	-2.8247249681
H	-8.1624755197	1.0504490537	-1.1230840233
H	-3.6705694805	-6.2474329618	0.3041107174
O	-2.093456839	3.0167435312	-0.1740415301
O	0.8410325417	-2.4356845208	-2.8401355355
O	-3.5477209487	1.1353599525	-1.3888356963
O	-1.7439319567	-3.0799148486	-2.3814348984
Si	-3.4422773339	2.7600820106	-1.0922929686
Si	-0.2280485726	-3.68856272	-2.6664745979
H	-3.3603817787	3.5242265336	-2.3394510831
H	-0.2304791647	-4.4882935673	-3.8939756619
O	-4.761820242	3.2323221609	-0.2147496694
O	0.2263440245	-4.5768066746	-1.3601261351
Si	-1.0622977055	2.2107561662	0.8234635407
Si	1.5788739241	-1.3107871742	-1.9167827558
O	-0.0847576081	1.2363825404	-0.0771790758
O	0.9975123692	0.1898335682	-2.3097019927
H	-3.0369448428	-4.8183968265	2.2007402879
H	-7.8220811178	-0.4898623548	0.7627048109
Si	0.9160214796	4.4924382588	1.0343731305
O	-0.1318855372	3.3296790008	1.5901547587
O	3.2077667431	-1.3923173124	-2.1814705298
H	0.8748836548	5.6567780173	1.9235857841
Si	-7.2177964443	0.5548485447	-0.0965199246
Si	-2.7752088867	-5.1718140414	0.7845396702
Si	-4.2707773622	-0.1750073008	-0.698426478
Si	-2.4763406369	-2.5468845358	-1.0085447674

O	-5.8930600362	-0.0690010045	-0.8848638162
O	-3.0323285133	-3.8161385066	-0.1422463835
O	-3.717528783	-1.5468189698	-1.3888717432
O	1.2866761669	-1.5097763268	-0.3076852713
O	-1.8771078395	1.2917628214	1.8999403242
O	-1.3854909566	-1.6874528072	-0.1216816134
O	-3.927682204	-0.1828096463	0.9185047293
Si	0.0313982905	-1.8720813458	0.712551925
Si	-3.4273438463	0.774647035	2.1675283515
H	-3.4712508974	0.0330729646	3.4298926914
H	0.0437689731	-1.0570696529	1.9214968862
Si	-5.5144174116	2.8519307485	1.2149390393
Si	0.2031570997	-4.9153982077	0.2581448126
O	-6.7504563072	1.8149556271	0.8824432361
O	-1.2052052267	-5.6832974114	0.6112744352
H	-6.025531311	4.0603804123	1.8688912172
O	1.4684376431	-5.8477350463	0.6852196523
O	0.2087892966	-3.4742337317	1.0833984055
O	-4.4170886583	2.1020374678	2.2063747444
H	2.3104799759	-5.4294450172	0.4352401993
H	3.6816252136	-0.8629628923	-1.5083215494
O	5.4730588146	-0.038686902	1.9921696993
C	4.6048386876	-0.7794624787	1.2556059599
C	5.7086333308	-0.609436425	3.2480355864
C	4.310451571	-2.1280615832	1.9728341286
C	4.8887977721	-1.8714151931	3.367764818
H	5.4963130841	-2.6884269402	3.7796858056
H	4.0543879814	-1.6770395345	4.0576986391
O	4.222259193	-0.4105908918	0.1815472354
O	6.4514559399	-0.1014463024	4.0210958504
O	2.9508477121	-2.4039804936	2.0713919732
H	2.6388631901	-2.8675307426	1.2745121459
C	5.074030677	-3.2559539724	1.212362487
H	6.1388229328	-3.0046227693	1.1132112695
H	4.9608812378	-4.1760343131	1.8031067096
C	4.4345523583	-3.4552383494	-0.1414587114
O	3.3307511327	-3.9649053496	-0.2606728565
O	5.1088529864	-2.941765794	-1.1500974316

H	4.468658648	-2.7650197591	-1.8842497603
S	2.1748583597	0.5735743061	3.0747330399
H	1.8233472277	0.9220588812	1.8185754956
C	2.2185017636	2.246073318	3.8097824095
H	1.2247061442	2.7088813995	3.7586740219
H	2.5021982095	2.1074748734	4.8620600856
H	2.9568083124	2.8864858484	3.3126186195
H	2.9568083124	2.8864858484	3.3126186195

SI DFT 035
E(RB3LYP) = -8159.11778394 A.U.
First frequency = 11.1304 cm^{-1}

O	0.2672548331	5.7569311781	0.3691937214
O	1.3818368986	3.6117927519	1.5907359807
Si	1.6919109399	5.0470055291	0.8108579932
H	2.461808803	5.9216654427	1.6996023563
O	2.5397334804	4.7228827985	-0.5769591457
Si	0.5959844343	2.2594599099	1.0839363311
O	-0.9965751649	4.691153741	-1.7965894952
O	-0.4162569256	2.7515218948	-0.1375177244
Si	-0.4105059483	3.1469189522	-1.7637616726
H	-1.1936414615	2.2375656196	-2.5951976245
O	1.1699794741	3.137765789	-2.2590820236
Si	-0.6852167013	6.0956074719	-0.9544025203
H	-1.964787628	6.6519967623	-0.4655983617
H	0.0303207401	7.0351370612	-1.8526926266
H	7.0290780316	-5.379471738	0.6155372016
H	-1.6889901362	-6.9077042433	0.4871111943
O	4.2580925901	0.5394340557	0.6851116043
O	-1.0341130323	-0.8764208984	3.0491157322
O	3.9657231825	-2.0011972065	1.4513618781
O	-0.2578342215	-3.4168816541	2.5260662174
Si	5.0556738686	-0.7641499793	1.3120085026
Si	-1.5105352099	-2.4614913352	3.0309832239
H	5.6342199138	-0.4170333272	2.6122667645
H	-1.9459773038	-2.8917754246	4.3627149835
O	6.2451838762	-1.2238007644	0.2596076914

O	-2.7371607848	-2.5584701526	1.920700159
Si	3.0384587628	0.8974628967	-0.3463573489
Si	-1.4108481431	0.3804685221	2.052513447
O	1.6520603356	1.1587452884	0.4933920223
O	-0.3134327868	1.6000172197	2.2680514806
H	-1.5470091385	-5.7430565097	-1.6734330804
H	5.5472444907	-5.881700722	-1.2807262916
Si	2.6880531012	3.5909276735	-1.7757785276
O	3.4475034093	2.2549054385	-1.1747565561
O	-2.9016584122	0.9828506668	2.3490511838
H	3.4580201023	4.1435424859	-2.8935169967
Si	5.9573734108	-4.8759802595	-0.2729879995
Si	-1.5796783155	-5.6008760785	-0.1986129934
Si	3.4700370247	-3.3484462379	0.6465615415
Si	0.5145881059	-3.6132215961	1.0845202854
O	4.6533709524	-4.4796946811	0.6848443384
O	-0.1692548817	-4.8547682697	0.2692033561
O	2.1026385723	-3.9221956458	1.3320344312
O	-1.2656617739	-0.0955902327	0.4779595516
O	2.7571366405	-0.3375153774	-1.3885388664
O	0.3782756261	-2.2169798301	0.2131600574
O	3.1799427515	-2.9666872216	-0.9341867553
Si	-0.8169623747	-1.331301548	-0.5152380622
Si	3.419961364	-1.7232286681	-1.9944706079
H	2.8078640684	-2.0350935337	-3.288746031
H	-0.3674383476	-0.8222093542	-1.8108899018
Si	6.4031024597	-1.8930044311	-1.2496503185
Si	-3.1596675403	-3.0661498006	0.3998718799
O	6.4983547513	-3.5289373498	-1.0817023605
O	-2.8860720079	-4.6781488285	0.2573107734
H	7.6028302027	-1.3808963965	-1.9184248394
O	-4.725975588	-2.7332316429	0.111846753
O	-2.1406783211	-2.3138267527	-0.6776459272
O	5.0567671049	-1.5029636947	-2.1370383472
H	-5.0148168565	-1.8559003693	0.4242649799
H	-3.6693520015	0.4891290536	2.0066331013
O	-6.2371700177	0.1288389842	-0.952328827
C	-5.6544754249	0.6631125837	0.1373340188

C	-6.6856219278	1.1389333992	-1.8456288893
C	-6.0030235134	2.166628661	0.2097153795
C	-6.2550214699	2.4681156095	-1.2727717126
H	-5.3152963559	2.7313934214	-1.7814664106
H	-7.0065950572	3.2495239519	-1.4449228942
O	-5.0744408927	-0.0304092815	0.9389760395
O	-7.2972233834	0.8554695572	-2.8170094691
O	-7.1887570691	2.2615518801	0.986162046
H	-7.9307420855	1.8517484443	0.5189732081
C	-4.9750911674	3.0203452611	0.9342780265
H	-5.3364987029	4.059332824	0.9772991688
H	-4.8890533085	2.6786112197	1.9765788405
C	-3.6005219816	2.9760216096	0.3202882939
O	-3.3279664527	2.2845340452	-0.6422631078
O	-2.7302768732	3.7614122615	0.9304832845
H	-1.8396520924	3.5361952927	0.5671510825
S	-3.5591227256	0.1741760086	-2.8843440347
H	-3.2410661653	0.0660065192	-1.5802842162
C	-4.1162832408	-1.5479869138	-3.1436115878
H	-3.2750953914	-2.2471285504	-3.0575239382
H	-4.8920149988	-1.8184140944	-2.4161344038
H	-4.5349949974	-1.5908761413	-4.1582946095
H	-4.5349949974	-1.5908761413	-4.1582946095

SI DFT 036
E(RB3LYP) = -8159.08258084 A.U.
First frequency = -287.7150 cm^{-1}

O	-2.8020750048	5.636231264	-1.3576319401
O	-2.0931982723	3.0549432988	-1.3544905569
Si	-3.2754204391	4.124419735	-0.9024891712
H	-4.5509928139	3.7819728359	-1.5363552565
O	-3.4179983292	4.0955690873	0.7471984156
Si	-0.6827872725	2.3739734844	-0.8634718579
O	-0.5139614898	6.1286737473	0.0258486432
O	0.2764937072	3.5803049813	-0.225550051
Si	0.0510876329	4.8310479661	0.8647122332
H	1.3245346761	5.152322179	1.5110135764

O	-1.061959562	4.3791753379	1.9930445437
Si	-1.4485807869	6.5978555484	-1.2694260565
H	-0.6608204987	6.4471236708	-2.5163213276
H	-1.8789869092	7.9942569256	-1.0397010272
H	-6.9503394467	-4.6158898	-0.6293315083
H	1.4818628533	-6.601574769	-1.6909146038
O	-3.6698426435	0.8774116118	0.3598710847
O	1.2173902463	-0.458397726	-3.2660200114
O	-3.7598317783	-1.3686382418	-1.0723438207
O	0.313796434	-2.9673442302	-2.8624722612
Si	-4.6735237899	-0.1261328694	-0.4776552814
Si	1.5927406294	-2.056043691	-3.4039797202
H	-5.3355526764	0.6104491067	-1.5570349144
H	1.8772506791	-2.4016353571	-4.7992713507
O	-5.7963393584	-0.7460730225	0.5674681512
O	2.8866003662	-2.3805034306	-2.4240228601
Si	-2.3100543172	0.9393322294	1.2843436077
Si	1.4689400902	0.7576488284	-2.1817484759
O	-1.0194748394	1.2757532024	0.3217414136
O	0.0782512018	1.6612421793	-2.105875667
H	1.6204286529	-6.1096684774	0.7098640144
H	-5.3730379247	-5.6803157481	0.926817234
Si	-2.6014986555	3.7933070208	2.1520610214
O	-2.5124946645	2.1555474517	2.3719692552
O	2.6898928897	1.7436829303	-2.5795321219
H	-3.2727632112	4.4121675643	3.2983438908
Si	-5.7595939927	-4.4307652244	0.2308930129
Si	1.5523773787	-5.5417170133	-0.6584888281
Si	-3.2583632084	-2.869393971	-0.6210323253
Si	-0.3893541628	-3.2095305804	-1.3978864781
O	-4.5052536147	-3.9236480069	-0.7386712284
O	0.1922574347	-4.5906625599	-0.7336655011
O	-2.0136923526	-3.3339698915	-1.5678843617
O	1.6894648348	0.1002734	-0.6824551568
O	-2.046576694	-0.4753978188	2.0599232035
O	-0.0513437884	-1.934682848	-0.4042212532
O	-2.7620870047	-2.8348829912	0.9544992612
Si	1.2659204736	-1.1730296395	0.2559967845

Si	-2.8002215073	-1.9223162167	2.3278453434
H	-2.1221418016	-2.621228779	3.4230740878
H	1.0096239111	-0.7798616633	1.6432750131
Si	-5.8480578745	-1.7339625604	1.8984419771
Si	3.3573049759	-3.0430240065	-0.988992368
O	-6.0855870687	-3.2814791103	1.3863001231
O	2.8999002607	-4.6214528087	-0.9456589584
H	-6.9279060783	-1.3258343471	2.8020045832
O	4.9596509374	-2.9615617127	-0.7272364178
O	2.5413188972	-2.2452183131	0.2333137445
O	-4.3912746566	-1.6313875158	2.6863247881
H	5.2902114878	-2.0397572996	-0.553645554
H	3.5602072563	1.5291893224	-2.1759163768
O	5.4992043713	-0.6096644259	0.2829052845
C	5.2602915342	0.5887258128	-0.1123404017
C	5.8446003205	-0.1654828607	2.7085696121
C	5.6724685525	1.6579774219	0.9450381597
C	5.2705577143	1.1715986542	2.3719133324
H	4.1757243072	1.1758362707	2.4523869561
H	5.6999750393	1.8821566626	3.0951303685
O	4.8152897697	0.9260906926	-1.2029601476
O	6.9371321932	-0.5015112471	3.0006905919
O	7.0703166586	1.8425184547	0.8755007601
H	7.4958909134	0.9728568134	0.8993390928
C	5.0346907002	3.008393453	0.6286593171
H	5.2964311146	3.7366931659	1.4139259095
H	5.4613178775	3.3679478627	-0.3147323549
C	3.5256113427	2.9427345692	0.5018090963
O	2.8104851475	2.2356335298	1.185348342
O	3.0633551454	3.7666330264	-0.4277663134
H	2.0932360564	3.6224685398	-0.5150506852
S	4.4000481159	-1.5433072737	2.7412415392
H	4.3373291394	-1.257520484	1.3816529817
C	5.4271751003	-3.0376766199	2.6005983846
H	4.994927359	-3.810581031	3.2463165357
H	5.4378763856	-3.3455789421	1.5453729188
H	6.4369834817	-2.7599878518	2.9384363918
H	6.4369834817	-2.7599878518	2.9384363918

SI DFT 037
E(RB3LYP) = -8159.08811755 A.U.
First frequency = -205.7257 cm^{-1}

O	-1.8492902804	-4.866685394	-2.7289786094
O	-0.340587113	-2.6586551277	-2.2890394077
Si	-0.4731382585	-4.2968251657	-2.0369346411
H	0.6976910807	-4.9731025791	-2.6039368536
O	-0.5653844726	-4.5748499157	-0.402735412
Si	-1.0863020492	-1.4152233976	-1.5013876758
O	-3.8934910948	-4.1932103076	-1.050722464
O	-2.5643976302	-1.9357982934	-1.0406146587
Si	-3.4868063906	-2.9088553173	-0.0695962363
H	-4.7339849943	-2.3545888406	0.4415705982
O	-2.5201872314	-3.5283723149	1.1417425898
Si	-3.5054605796	-4.7777547155	-2.5505902947
H	-4.0370895967	-3.882684193	-3.6083041969
H	-4.0525713208	-6.1493281324	-2.6658425632
H	8.0584509431	-1.004361314	-1.1039708114
H	3.5817352982	6.4737840747	-0.2442166855
O	1.9784341117	-2.7148086004	-0.1991963564
O	-0.7883597142	2.4705482829	-3.0385885444
O	3.4910029419	-0.9507489847	-1.5238986729
O	1.7483807026	3.3164572007	-2.6161387407
Si	3.3346131941	-2.5488691795	-1.125091986
Si	0.1816591945	3.7955611503	-2.8969562829
H	3.2513269497	-3.3911424018	-2.3194240796
H	0.1317368416	4.5883150254	-4.1286573435
O	4.629585401	-2.9932189796	-0.1935633562
O	-0.3345247869	4.6641202706	-1.5938566748
Si	0.9094822722	-1.9077411021	0.7478102762
Si	-1.6687079681	1.4630513308	-2.0899961231
O	-0.1512064835	-1.0563599994	-0.1712154625
O	-1.2212339318	-0.0933231458	-2.4423730527
H	3.1050538254	5.2693582053	1.8420691312
H	7.7629181045	0.6865060214	0.6561381748
Si	-1.0029563989	-4.1672014186	1.1337136677
O	0.0731550858	-2.9997033719	1.6535421154

O	-3.25938151	1.7548495228	-2.3427499254
H	-0.919011415	-5.317804057	2.040902843
Si	7.1225506982	-0.3895517018	-0.1351186662
Si	2.7542562849	5.4353821419	0.4110349335
Si	4.215615	0.375538496	-0.8681091055
Si	2.4655334698	2.7379857566	-1.2554319027
O	5.8412969082	0.2352287772	-0.99118869
O	3.0178177139	3.9782987695	-0.3442406018
O	3.707926893	1.730973555	-1.6250134429
O	-1.3111173508	1.6770109032	-0.4876405145
O	1.6889362928	-0.8837704612	1.7698322969
O	1.3622646857	1.8561501229	-0.4065407199
O	3.8227686671	0.4523793614	0.7379990047
Si	-0.0268710838	2.0470079403	0.470468321
Si	3.2584289255	-0.408377754	2.0246130109
H	3.2982508754	0.4062371201	3.2431221487
H	0.039186721	1.2437457104	1.6832017698
Si	5.3246564611	-2.5639812781	1.2498023547
Si	-0.2441359962	5.0573983077	0.0090040233
O	6.5810048874	-1.5474771222	0.9295482622
O	1.1604246455	5.8714243048	0.2656388321
H	5.7956935063	-3.7478608193	1.9754381974
O	-1.5025247617	5.9808881561	0.4733781421
O	-0.1713518642	3.6439095275	0.8817543974
O	4.1944210995	-1.7668311978	2.1691295348
H	-2.3421091166	5.4955793498	0.4063880156
H	-3.8008620473	1.2428386207	-1.6962728539
O	-3.1759346768	-0.5963289561	1.4331441105
C	-3.7635984307	0.4219801529	0.9508056621
C	-3.5794530311	-0.2959668051	3.6635981829
C	-3.9264215095	1.5832756847	1.9891550639
C	-4.3685723093	0.9367953301	3.3039333607
H	-5.4162017702	0.6053567656	3.2581752162
H	-4.2500845163	1.6606949366	4.1264973615
O	-4.2121707572	0.5612354654	-0.1892993582
O	-3.9401821357	-1.3112816646	4.1381064607
O	-2.6702417326	2.1755077585	2.2652007957
H	-2.4979359858	2.8644608205	1.5904658854

C	-4.9488583066	2.6245048057	1.4853061452
H	-5.9107369571	2.1457642485	1.2688927101
H	-5.0837854981	3.3949644236	2.2610992792
C	-4.4152931772	3.3038603012	0.2454722306
O	-3.3232437996	3.8563023427	0.2409737752
O	-5.1991865374	3.2596264133	-0.813296341
H	-4.6589222954	3.4863141234	-1.5972327908
S	-1.5500729454	0.0142621095	3.6992276556
H	-1.6439651721	-0.2845566805	2.3619066057
C	-1.0721092395	-1.6185668927	4.3253009325
H	-0.1996065282	-1.9534631791	3.7519610015
H	-0.8325583353	-1.5109852544	5.3903089051
H	-1.9133118571	-2.3105087051	4.1800896843
H	-1.9133118571	-2.3105087051	4.1800896843

SI DFT 038
E(RB3LYP) = -8159.13236682 A.U.
First frequency = 17.4976 cm^{-1}

O	1.9937519907	4.870021161	-2.7480257951
O	0.3862703177	2.7365367536	-2.2987589291
Si	0.5935249858	4.3681488317	-2.0467124576
H	-0.5461678595	5.0977584796	-2.6094120626
O	0.7169994962	4.6450184339	-0.4160735268
Si	1.0944362787	1.476108503	-1.5056156258
O	4.0881417402	4.1325446198	-1.1555951778
O	2.6124650276	1.9501360047	-1.1060150758
Si	3.5868027898	2.9039160302	-0.1667458932
H	4.7377197725	2.1991571084	0.3922503173
O	2.6741141285	3.5506182358	1.0642292695
Si	3.6500309523	4.7491567247	-2.6368411107
H	4.1275784076	3.8514752807	-3.7168041563
H	4.2262277813	6.1079778435	-2.748701533
H	-8.0258674412	1.2800357759	-0.8952651563
H	-3.7838102104	-6.2036469563	0.1246317685
O	-1.8628411442	2.9020877925	-0.1071093409
O	0.7237812647	-2.4506490696	-3.017869644
O	-3.4451069981	1.171917265	-1.3846629787

O	-1.8165767327	-3.1792470314	-2.4445544186
Si	-3.2445476347	2.7686430986	-0.9980442297
Si	-0.2931199657	-3.7348748722	-2.7911587946
H	-3.1661423325	3.6016326198	-2.1998083857
H	-0.3304692748	-4.5494866579	-4.0086550362
O	-4.5107394818	3.2493275152	-0.0467109248
O	0.2557245657	-4.5961479838	-1.4971267871
Si	-0.808987783	2.0412560895	0.815076367
Si	1.6098782349	-1.4292536669	-2.0958491518
O	0.1888033691	1.1626983268	-0.154345718
O	1.1715967561	0.1305782785	-2.4256227346
H	-3.0612124168	-4.9253660318	2.0950232644
H	-7.6814771337	-0.4018782376	0.8646193144
Si	1.1723564109	4.2386064531	1.1173725019
O	0.0934996531	3.107670838	1.6855016026
O	3.1993321105	-1.7153428351	-2.4055602768
H	1.1688580685	5.4045418673	2.0071004677
Si	-7.0617150719	0.6653951196	0.0453927105
Si	-2.8529238444	-5.1798421974	0.6492935246
Si	-4.1920867121	-0.1402577094	-0.7275416982
Si	-2.4988283764	-2.5513841574	-1.0871420749
O	-5.8152765393	0.0256220157	-0.8532950447
O	-3.1105058541	-3.7521823981	-0.1622469643
O	-3.6912951315	-1.4991427687	-1.4840012571
O	1.3235227028	-1.6802948377	-0.4901119449
O	-1.6081089679	1.0345291235	1.8298315109
O	-1.3463477665	-1.7213758656	-0.2462447251
O	-3.805652824	-0.2355083842	0.8772436297
Si	0.0788515258	-1.9649881509	0.556217904
Si	-3.179230416	0.6136837187	2.1455152872
H	-3.213702535	-0.1936414762	3.3679437397
H	0.1565553006	-1.1353585224	1.7553446693
Si	-5.2004057179	2.819805605	1.3999492212
Si	0.1486703291	-4.9827674856	0.10802622
O	-6.4760618385	1.8266949382	1.0813653225
O	-1.3018835363	-5.7035689584	0.3796081435
H	-5.6475815049	4.0064798918	2.1357162752
O	1.3427671164	-5.9919396267	0.5632367604

O	0.1786250062	-3.5633369885	0.9767106508
O	-4.0743065036	1.9987821257	2.3015110588
H	2.2158736632	-5.5791485149	0.4638446683
H	3.8072949054	-1.1580721928	-1.883137416
O	2.7954325201	0.1868978814	1.0861746288
C	3.7934984312	-0.640308248	0.8216227822
C	4.2008837604	0.2796374985	3.5478869289
C	4.0715547722	-1.7224485688	1.8973454119
C	4.754717762	-1.0795793579	3.135048864
H	5.8206065377	-0.9141345676	2.9295588305
H	4.6430232135	-1.8038450911	3.9561332078
O	4.4454081826	-0.5381805965	-0.1934053903
O	4.8218169471	1.2999248518	3.3967514037
O	2.8514431592	-2.2723545894	2.3339077783
H	2.5781780433	-2.9680004824	1.7024589487
C	5.0157832253	-2.7930548397	1.296858924
H	5.9645336677	-2.3383365189	0.993585299
H	5.1955600208	-3.5527024433	2.0724875413
C	4.354661319	-3.4539857234	0.1119013049
O	3.2578974553	-3.9849824726	0.201976079
O	5.0242870188	-3.361657233	-1.0200420767
H	4.4126552881	-3.5442637741	-1.7620448405
S	2.5154913116	0.2488884333	4.1959817863
H	2.3268562977	-0.062681335	1.9127751471
C	2.1959160862	2.040594322	4.2740694092
H	1.1294244381	2.1870230994	4.0603611635
H	2.4497598954	2.4309553581	5.2683026169
H	2.8029585439	2.5374335057	3.5058951284
H	2.8029585439	2.5374335057	3.5058951284

SI DFT 039
E(RB3LYP) = -8159.11919023 A.U.
First frequency = -10.8180 cm^{-1}

O	2.9441943509	5.401518151	2.0707847792
O	1.8838976992	2.9910308036	1.6647788708
Si	3.2409875347	3.9127094424	1.4280004784
H	4.4091050136	3.3067121791	2.0714131283

O	3.4960039346	4.0758980635	-0.1998107988
Si	0.4740828189	2.4180444806	1.0563684421
O	0.6531489507	6.1784072702	0.8362108912
O	-0.3748109802	3.7157144277	0.4375108965
Si	0.0656086532	5.1539691423	-0.3030380934
H	-1.1215677725	5.7204502187	-0.946971592
O	1.2349525647	4.839045697	-1.4216029426
Si	1.6478665767	6.4411393406	2.1437506946
H	0.8792846994	6.1877104042	3.3852950584
H	2.1569220311	7.828053712	2.0685955754
H	6.7925181217	-4.4479491505	0.2782387609
H	-1.0807960599	-6.6527425126	1.1828957507
O	3.4325790922	0.9079517366	-0.2008028462
O	-1.5284626156	-0.4597496705	3.3133869001
O	4.0952767737	-1.3648548617	1.0297458836
O	-0.3286570228	-2.8410227639	2.8716590528
Si	4.7016573246	-0.0095988465	0.30558932
Si	-1.7023215547	-2.0927973708	3.4260468851
H	5.5245940963	0.752951584	1.2471024383
H	-1.9466927873	-2.4858863065	4.8160286638
O	5.595306061	-0.4658810983	-1.0092515833
O	-2.958502451	-2.5579388773	2.4475758455
Si	2.1102667393	1.1362275791	-1.1503803984
Si	-1.7792576304	0.7695752757	2.2454288361
O	0.8081637273	1.3716195981	-0.1723918356
O	-0.3994341396	1.6852225603	2.2084578883
H	-1.3140641558	-5.7005787994	-1.068925632
H	5.2644306959	-5.5476189134	-1.3021568943
Si	2.6786876604	4.0599549931	-1.6380562568
O	2.3718277636	2.4868676251	-2.0507112737
O	-3.0146699018	1.7320969536	2.6630394677
H	3.4549998037	4.7131652712	-2.6949396013
Si	5.5706618353	-4.3103417307	-0.5478620561
Si	-1.2834979183	-5.4212937527	0.3871373801
Si	3.2541600298	-2.6972678728	0.5564753169
Si	0.4133711349	-2.9677604396	1.4109429013
O	4.2849996153	-3.9655834912	0.4548431595
O	-0.0416854141	-4.3591309342	0.6738648586

O	2.0396676914	-2.9847626878	1.6109555249
O	-1.9967428685	0.1353625139	0.7356259728
O	1.8449942726	-0.1506139715	-2.1211858794
O	-0.0197053113	-1.6759183065	0.4779432156
O	2.5940291601	-2.4459101143	-0.93233721
Si	-1.4063043269	-1.0753374098	-0.2111484045
Si	2.4921926915	-1.6518330634	-2.3715159839
H	1.644714382	-2.3820232037	-3.3178974555
H	-1.179407879	-0.6455233978	-1.5897945268
Si	5.5409540732	-1.5654917293	-2.2486148506
Si	-3.2997447852	-3.2045757375	0.9698451862
O	5.7864106114	-3.0726071451	-1.6323789431
O	-2.7219010144	-4.7363845107	0.8610125572
H	6.5658160822	-1.2483114445	-3.2473562367
O	-4.9182488754	-3.2672680096	0.7087080643
O	-2.5318273388	-2.299605271	-0.1889881615
O	4.0371799922	-1.4953973833	-2.9466258066
H	-5.3687091333	-2.4268084031	0.8820929718
H	-3.8889431322	1.5890290318	2.251522069
O	-5.000798187	-0.5650004832	0.0714694985
C	-5.2063458453	0.7598106724	0.0472905143
C	-5.7356494801	-0.5927994091	-2.7418495697
C	-5.531554279	1.4841109008	-1.2981817714
C	-5.0582783934	0.7496233902	-2.5758233497
H	-3.9627526913	0.6763340702	-2.5880903905
H	-5.3691878302	1.3791521177	-3.4247002044
O	-5.2276945891	1.3729081921	1.0854481996
O	-6.9406801147	-0.7375769629	-2.7052777717
O	-6.9286236702	1.6686106793	-1.3265856089
H	-7.3315945844	0.8727576053	-1.7199273181
C	-4.9065893081	2.8844065794	-1.2043099608
H	-5.0545087936	3.3979743947	-2.1666680104
H	-5.4446951649	3.4392837203	-0.4283359848
C	-3.4260927094	2.8484239118	-0.8793464709
O	-2.6318834817	2.1123600298	-1.4300213379
O	-3.0971234639	3.7003881677	0.0813057291
H	-2.1334956518	3.6096758052	0.27526012
S	-4.5695664092	-1.9364976904	-2.852993195

H	-4.7236716692	-0.9667328543	-0.7786798294
C	-5.6717660053	-3.3734079211	-2.6737422095
H	-5.3430770687	-4.1497554066	-3.3758640708
H	-5.6109436582	-3.7316921684	-1.6366735366
H	-6.6929001412	-3.0441262149	-2.9102458937
H	-6.6929001412	-3.0441262149	-2.9102458937

14.2.2 C_b selectivity

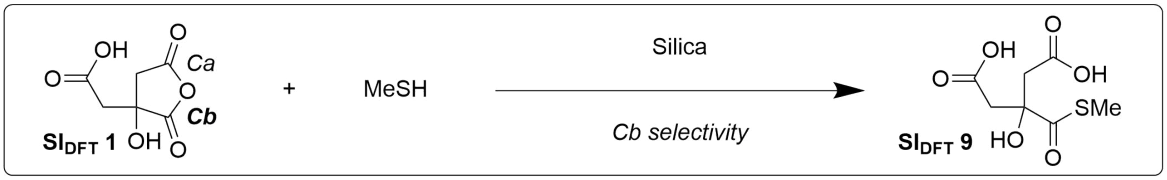

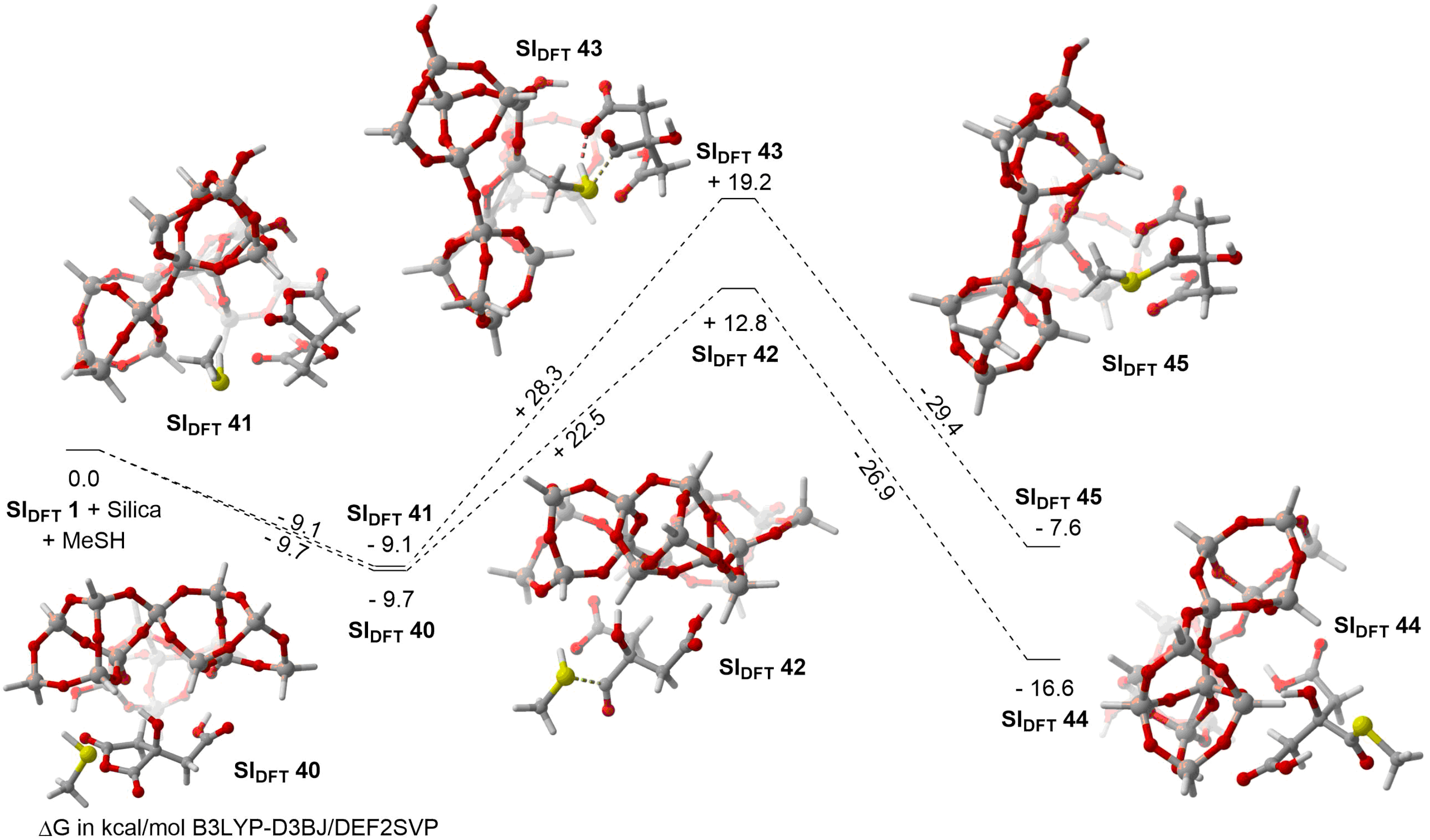

Figure S34: Nucleophilic addition of MeSH to the C_b site of citric anhydride with a model of silica as catalyst. The Gibbs free energies relative to the starting materials are given in kcal/mol.

SI DFT 040
E(RB3LYP) = -8159.11610694 A.U.
First frequency = 12.5655 cm^{-1}

O	4.9785285505	-1.1617700598	1.1250297859
O	2.5608485589	-0.2549246766	0.3733947229
Si	4.0981085877	0.2074315455	0.8851365183
H	4.7015327882	1.0759360926	-0.1214839572
O	3.948496348	0.991365943	2.325459647
Si	1.1999802218	-0.7226707567	1.213779023
O	4.0386190021	-2.4501845884	3.3251491202
O	1.6512789562	-1.6304926375	2.4993900712
Si	2.6201443425	-1.7814409234	3.8365186715
H	1.981795347	-2.6149701794	4.8580315333
O	2.8952081636	-0.2635710499	4.4391902651
Si	4.8447693588	-2.6429437276	1.8806275557
H	4.0827944794	-3.5590609227	1.0010182525
H	6.210611749	-3.1259707389	2.1743415012
H	-1.0209208902	4.8959647489	-3.6414397258
H	-7.239632404	-0.4132360396	-1.3512307737
O	1.6346570833	2.8270093693	0.9554605656
O	-1.2740426695	-3.3545148922	-1.0145582465
O	-0.2110261779	2.3854672428	-0.8804272766
O	-3.1181582006	-1.5282079836	-1.7118667785
Si	1.1777095879	3.2415771153	-0.5885890483
Si	-2.7774726478	-3.1476938089	-1.6628564318
H	2.2483129468	2.9497420687	-1.5360255068
H	-2.8142793381	-3.7168616659	-3.0124853424
O	0.8074229883	4.8529902926	-0.6081351542
O	-3.8944665405	-3.8668742719	-0.6772791499
Si	0.8173793543	2.1279664747	2.2043298037
Si	-0.4634146841	-3.0946253404	0.3986980482
O	0.3914423323	0.5987104931	1.7374460627
O	0.2702844838	-1.6101792454	0.2154621026
H	-6.8403621661	-0.1990750345	1.0693116445
H	-3.3125945949	5.6288066321	-3.0966638999
Si	3.2219259799	1.2243106359	3.7927871345
O	1.8073683844	2.0375138445	3.5103323362
O	0.6233763305	-4.2668370921	0.6992565538
H	4.0749883011	1.9972434593	4.6986808171
Si	-2.0543775284	5.0376294808	-2.5927953425
Si	-6.3123642627	-0.6769360317	-0.2303442009

Si	-1.849457052	2.4388675664	-0.895557152
Si	-3.2906550753	-0.2787546007	-0.6538012357
O	-2.4154289211	3.5461532115	-1.9476555843
O	-4.8730155653	0.1130771712	-0.5257023342
O	-2.4365807348	0.9582356324	-1.3130407226
O	-1.5226312851	-2.9612400885	1.651651924
O	-0.5344179651	2.9594281117	2.5869919325
O	-2.7009064622	-0.708457681	0.81877812
O	-2.3739892069	2.7978555904	0.6272352123
Si	-2.7799831123	-1.9260892092	1.9460200971
Si	-1.7785129983	3.7576204212	1.8393600131
H	-2.8247722261	4.0511181692	2.8213451316
H	-2.7036895545	-1.3873077744	3.3059603234
Si	-0.4186660381	5.8541615855	-0.1145733168
Si	-5.0141874967	-3.431425581	0.4603830711
O	-1.4800778609	6.0109621414	-1.3659639024
O	-6.034592011	-2.3127580471	-0.1660585056
H	0.1020817954	7.1661041738	0.281481744
O	-5.9014568363	-4.7175105787	0.9326454175
O	-4.2018840405	-2.7376021013	1.7281807264
O	-1.1871699225	5.1471655693	1.17284976
H	-5.4268763186	-5.5108194044	1.2059588792
H	1.3438480107	-4.2719943677	0.0422510991
O	3.4945937464	-2.2933631312	-2.9232981679
C	3.2550048092	-1.1653562511	-3.7160263162
C	2.372017883	-2.648249823	-2.218616508
C	1.9038518454	-0.5644566161	-3.2699509311
C	1.2163999068	-1.8070347042	-2.6985326675
H	0.6938159145	-2.3751777632	-3.4844689443
H	0.49622485	-1.5882768437	-1.9099763269
O	4.0298973239	-0.8007890993	-4.5380943002
O	2.4105120454	-3.5164733351	-1.3904899215
O	2.1678133906	0.4044437965	-2.2789639375
H	2.3287379484	0.0097596904	-1.4002681965
C	1.1815825071	0.1606465311	-4.4022015548
H	1.8344153311	0.9518118839	-4.789803014
H	0.9452371709	-0.5433602543	-5.2144936604
C	-0.0950766283	0.7945458175	-3.8804382707

O	-0.3119273459	1.9791385752	-3.8245880606
O	-0.9605517681	-0.1407183457	-3.4602963642
H	-1.6884785968	0.2884020246	-2.9641210017
S	6.2920043117	-0.5179748691	-2.0311577161
H	6.6310626189	-0.9415293506	-0.7934083929
C	6.6700490338	-2.0696572114	-2.9198460438
H	6.0934005567	-2.9092703632	-2.5104159562
H	6.344464347	-1.9027001153	-3.9546471413
H	7.7465745543	-2.2893209245	-2.9016682048
H	7.7465745543	-2.2893209245	-2.9016682048

SI DFT 041
E(RB3LYP) = -8159.11575891 A.U.
First frequency = 12.1353 cm^{-1}

O	-4.6530560087	-1.543244813	-0.2732771545
O	-2.3139527717	-0.2334513209	-0.2747626015
Si	-3.94889376	-0.0113204396	-0.2722973474
H	-4.4597351744	0.8065749083	0.8190973356
O	-4.4063860223	0.6616779984	-1.7174437606
Si	-1.0408858546	-0.362341638	-1.3160980165
O	-3.8294347078	-2.4418730973	-2.7302512155
O	-1.5422501528	-1.0647575902	-2.7144576138
Si	-2.8123845272	-1.5195224177	-3.6539016953
H	-2.3686481696	-2.2735849904	-4.8273699437
O	-3.5956772782	-0.1282241816	-4.1194741339
Si	-5.0364529704	-2.5104915789	-1.6063100873
H	-5.1486007908	-3.8871566973	-1.0821782636
H	-6.3014140727	-2.0182166159	-2.2012045771
H	0.731407752	4.4518533663	4.1791040134
H	7.1215756727	0.4394918751	1.2685255418
O	-2.2919174742	2.8185100499	-0.4834814244
O	1.6430620883	-3.3392750613	0.069668516
O	-0.2755814277	2.3672677792	1.2029999652
O	2.9526289419	-1.2144949281	1.0941626392
Si	-1.7929318217	3.0143920581	1.0878725622
Si	2.9393652783	-2.8626484487	0.9758486628
H	-2.7274411002	2.3656618984	2.0051042333

H	2.8945621463	-3.492940678	2.2973290236
O	-1.6996560296	4.6339076937	1.4087044716
O	4.3231136905	-3.3221220837	0.1893058529
Si	-1.5054533034	2.4953907577	-1.9024990454
Si	0.8141918821	-2.7061206129	-1.2084383077
O	-0.5464081393	1.1677477981	-1.6687412064
O	0.1481601033	-1.2711555859	-0.6850245318
H	6.6906657519	0.9033035368	-1.1071417748
H	2.7306809959	5.6379991279	3.3652725486
Si	-3.9679237432	1.2092635957	-3.2169228097
O	-2.6388037136	2.1858998079	-3.0465671961
O	-0.268300024	-3.7487695319	-1.8334324565
H	-5.0588605909	1.9545446755	-3.8510540713
Si	1.5001336536	4.9067128034	2.9945791268
Si	6.2348141068	0.1892748983	0.1111844862
Si	1.3077305281	2.6762806749	0.8707802378
Si	3.175388165	0.1712916108	0.2406660311
O	1.9304802084	3.57536535	2.0960040203
O	4.7042580957	0.7126383186	0.4992411514
O	2.1114988546	1.2791345765	0.761251196
O	1.8827510123	-2.3278320586	-2.4064838602
O	-0.554414003	3.7565899082	-2.3287616467
O	2.9883285254	-0.1158527849	-1.3688612925
O	1.423253055	3.5367310661	-0.527700959
Si	3.1655592733	-1.2962133423	-2.5174908944
Si	0.6022945517	4.6008288842	-1.4886227954
H	1.5045069077	5.2641534432	-2.4327812999
H	3.2325830158	-0.7078412231	-3.8582854017
Si	-0.7177406213	5.91350956	1.0242096118
Si	5.404445403	-2.6849071685	-0.8843632849
O	0.5548553055	5.9185315968	2.0734918549
O	6.2085219789	-1.4420938622	-0.1827185753
H	-1.4588727731	7.1753106929	1.1126777703
O	6.5140151684	-3.8010757406	-1.3285236159
O	4.5631546377	-2.1172651583	-2.1913503026
O	-0.1476040653	5.7087231338	-0.5171802182
H	6.1779313054	-4.6360283851	-1.6733882445
H	-1.0583455384	-4.0078929683	-1.3295645078

O	-1.0673514744	-3.1895759339	1.110107479
C	-0.6536992669	-2.9605770976	2.4079430522
C	-1.943479423	-4.2535578052	1.0466473586
C	-1.6175305533	-3.6925475903	3.356029982
C	-2.1555222841	-4.7991162707	2.437691193
H	-3.2037812042	-5.0775653122	2.5899824726
H	-1.5200268396	-5.6918561845	2.5575209959
O	0.3039658411	-2.3146987179	2.6847505645
O	-2.3613803908	-4.6197506818	-0.0131087258
O	-0.9255045297	-4.2387420016	4.4398200411
H	-0.3736972491	-3.5385393764	4.8228260651
C	-2.7014875149	-2.6949941368	3.8465904927
H	-3.4050217054	-3.2680626032	4.4680636331
H	-2.2047566851	-1.919431832	4.4466498297
C	-3.4280640323	-2.0043740357	2.7145601232
O	-3.2340908831	-0.8521866661	2.3957063172
O	-4.2505916199	-2.8170782069	2.0500745787
H	-4.5498440267	-2.3472922474	1.2241094352
S	-0.7365613785	0.5533787264	4.1353114763
H	-1.015786348	0.3641877255	2.8303223302
C	1.0869075373	0.5927615257	3.9843597047
H	1.4930058915	0.4191971773	4.9907117286
H	1.4353338243	-0.2003443507	3.3120760753
H	1.4349838893	1.5693614544	3.6260130777
H	1.4349838893	1.5693614544	3.6260130777

SI DFT 042
E(RB3LYP) = -8159.08436179 A.U.
First frequency = -301.0117 cm^{-1}

O	-4.7504249067	-0.7124481368	-1.0645560349
O	-2.1933392896	0.0429533662	-0.654367816
Si	-3.6578354925	0.4760177831	-1.3561697501
H	-4.1154052277	1.7208627477	-0.7351763312
O	-3.4435065729	0.6532333442	-2.9774098487
Si	-0.8367702708	-0.8094342711	-1.1160807812
O	-3.7816349339	-2.7442558829	-2.6098661265
O	-1.2483249451	-1.9730640282	-2.1840767227

Si	-2.3199248473	-2.516822103	-3.3169823912
H	-1.8393091879	-3.7532726626	-3.936181143
O	-2.4276135254	-1.3122246005	-4.4638821075
Si	-5.0793258573	-2.2270632874	-1.7138122951
H	-5.421676074	-3.1766692127	-0.6454856996
H	-6.2112266323	-2.0350528353	-2.6589941401
H	0.9204445256	5.6048843972	2.4589270593
H	7.0355710984	-0.1776620533	2.3448155893
O	-1.1440406234	2.5981498491	-1.9796984756
O	1.0406499926	-3.1554780348	1.8771482912
O	0.1917140571	2.5310150433	0.3195639619
O	2.8212047916	-1.2018820649	2.3415913202
Si	-0.9950942164	3.3376643095	-0.5036841058
Si	2.4362455718	-2.781625427	2.6628283424
H	-2.2633254363	3.3088999435	0.2237639876
H	2.2717669323	-2.9843064963	4.1047683587
O	-0.5079031477	4.8975624154	-0.749434985
O	3.6767424605	-3.7099803001	2.0773976913
Si	-0.2035774303	1.543792115	-2.8403621505
Si	0.4137460806	-3.0565606137	0.3528072881
O	0.1611307556	0.2634735549	-1.8627670047
O	-0.1067095465	-1.4708399245	0.1779041281
H	6.8775235148	-0.4788478535	-0.092853171
H	3.3247537211	5.9982572807	2.0820244998
Si	-2.5572085943	0.3286007137	-4.3410050013
O	-1.0655982353	1.0294309783	-4.1370955227
O	-0.7842133821	-4.1107942385	0.1108215856
H	-3.2044141047	0.8820622817	-5.5333334434
Si	2.0819600359	5.3864291283	1.565770564
Si	6.2058337826	-0.654090681	1.2175655728
Si	1.8230390175	2.4824326444	0.5070957899
Si	3.2107148911	-0.2174485719	1.0873467671
O	2.3435753023	3.7537920269	1.3874164465
O	4.7836425181	0.2225033763	1.2009682203
O	2.21861855	1.0830600205	1.2682832778
O	1.6391248167	-3.2668844367	-0.7371585242
O	1.1933326621	2.266868928	-3.2911104977
O	2.9576078896	-0.9677324369	-0.3483598029

O	2.5442022483	2.5116638058	-0.9744129155
Si	3.0228060643	-2.4483832415	-1.0991810032
Si	2.3118386786	3.1781400767	-2.4718991516
H	3.5644740687	3.2070071027	-3.2300825911
H	3.174785307	-2.2837911536	-2.5471831925
Si	0.8523420452	5.6999269765	-1.2616962445
Si	4.9424835733	-3.5451623379	1.0268142714
O	1.7636170175	6.0452865388	0.0681604161
O	5.8576286048	-2.2560776847	1.4643618991
H	0.4928909136	6.9314464717	-1.9711526509
O	5.9013506157	-4.8677206076	1.0501052824
O	4.3223092844	-3.2644019653	-0.4824958248
O	1.7067429366	4.702945035	-2.2707277347
H	5.4746324366	-5.7224929485	0.9210030446
H	-1.6791158089	-3.7170204151	0.2969458821
O	-4.5636374945	-1.784640443	1.8642840647
C	-4.2098373783	-0.1848752632	3.4939010247
C	-3.3606777051	-2.0885733528	1.5768664983
C	-2.7834025923	-0.0656271791	2.9215362037
C	-2.3351982602	-1.4869285121	2.5527141535
H	-2.3149881424	-2.1061360384	3.4634335499
H	-1.3215242465	-1.4596861826	2.1436648691
O	-4.5916047005	-0.5410754363	4.547727498
O	-3.0027622474	-2.771625755	0.6042212684
O	-2.8698758002	0.7834397103	1.8095201215
H	-2.4136003407	0.4136135023	1.0251622932
C	-1.919177301	0.6298623319	4.0034137385
H	-2.4488238667	1.5111693719	4.3863362241
H	-1.7295652718	-0.0643741126	4.8352954167
C	-0.6065836114	1.131778174	3.4214868062
O	-0.2971275688	2.2958224031	3.3547919438
O	0.1596585069	0.1296997305	2.9835815487
H	0.9497948043	0.4958420531	2.5287754354
S	-5.4914826875	0.7821438808	2.2655495265
H	-5.2101557998	-0.307500578	1.4309233527
C	-6.9786900748	0.0624521168	3.0292084442
H	-6.9212957393	-1.0298198533	2.9442428881
H	-6.9857317396	0.3571909217	4.0867002042

H	-7.860602489	0.4612054618	2.5125718527
H	-7.860602489	0.4612054618	2.5125718527

SI DFT 043
E(RB3LYP) = -8159.07378783 A.U.
First frequency = -457.5948 cm^{-1}

O	4.45971	1.62948	-0.50895
O	2.27765	0.06068	-0.30049
Si	3.92581	0.03945	-0.45831
H	4.60816	-0.73942	0.56605
O	4.30436	-0.60806	-1.93814
Si	0.90622	0.18881	-1.21651
O	3.45193	2.38992	-2.94634
O	1.22486	0.93673	-2.63833
Si	2.36693	1.42173	-3.71858
H	1.76537	2.12073	-4.85507
O	3.13541	0.03814	-4.23922
Si	4.59581	2.71139	-1.80474
H	4.4002	4.05766	-1.23443
H	5.93732	2.52866	-2.41028
H	-1.6789	-4.94898	4.3774
H	-7.30667	-0.62292	0.68403
O	2.22511	-2.91317	-0.39025
O	-1.6681	3.10958	0.42108
O	0.20479	-2.20498	1.2451
O	-3.20787	0.97767	0.91266
Si	1.72787	-2.87512	1.18962
Si	-3.14315	2.63417	0.97277
H	2.64686	-2.10728	2.03052
H	-3.3269	3.0963	2.35319
O	1.62572	-4.43608	1.72652
O	-4.352	3.20959	0.00207
Si	1.43896	-2.63818	-1.82237
Si	-0.75306	2.57736	-0.85469
O	0.43122	-1.3498	-1.57993
O	-0.24105	1.02928	-0.42606
H	-6.72837	-0.88229	-1.69219

H	-3.0783	-5.75926	2.52681
Si	3.73302	-1.22822	-3.36033
O	2.5297	-2.31357	-2.99579
O	0.39013	3.58605	-1.37242
H	4.79172	-1.91124	-4.10892
Si	-1.83234	-5.05355	2.9077
Si	-6.334	-0.30438	-0.38408
Si	-1.33319	-2.7365	0.94169
Si	-3.31123	-0.37907	-0.01236
O	-1.85828	-3.50541	2.2965
O	-4.85394	-0.93454	0.05352
O	-2.30177	-1.48244	0.6273
O	-1.78492	2.32235	-2.12975
O	0.54173	-3.95643	-2.20424
O	-2.92436	-0.04667	-1.57294
O	-1.34074	-3.81027	-0.30177
Si	-2.96264	1.27498	-2.58209
Si	-0.47927	-4.85228	-1.25594
H	-1.36832	-5.64794	-2.1056
H	-2.80103	0.84538	-3.97428
Si	0.82078	-5.83928	1.36096
Si	-5.36153	2.58567	-1.15086
O	-0.54768	-5.89628	2.27862
O	-6.2229	1.34567	-0.508
H	1.66606	-7.00396	1.63975
O	-6.42551	3.71606	-1.66362
O	-4.43853	2.00365	-2.3922
O	0.40195	-5.82014	-0.24328
H	-6.05847	4.55023	-1.97797
H	1.07736	3.96189	-0.74998
O	1.19983	2.71983	1.36843
C	0.73409	2.47483	3.71908
C	1.7864	3.85674	1.40372
C	2.03473	3.32091	3.90533
C	2.04502	4.40188	2.80868
H	2.98639	4.96563	2.84123
H	1.22716	5.11142	3.02244
O	-0.33826	2.87502	4.02287

O	2.06602	4.53977	0.41418
O	1.9247	3.88874	5.19324
H	1.10238	4.39953	5.22955
C	3.32906	2.4935	3.97546
H	4.08901	3.1809	4.37088
H	3.19747	1.67064	4.69024
C	3.81041	1.92709	2.66059
O	3.61576	0.77562	2.32318
O	4.44558	2.81144	1.91075
H	4.5726	2.40885	1.00954
S	0.87806	0.59647	3.1404
H	1.15103	1.26127	1.91737
C	-0.893	0.26376	2.97493
H	-1.43665	1.04363	3.52551
H	-1.14981	0.29624	1.91474
H	-1.08699	-0.7288	3.3956
H	-1.08699	-0.7288	3.3956

SI DFT 044
E(RB3LYP) = -8159.13139077 A.U.
First frequency = 8.0734 cm^{-1}

O	4.5390912058	-1.8208024075	1.647960217
O	2.5113551641	-0.4550584828	0.4736453985
Si	4.0546124151	-0.3442435318	1.1452070845
H	4.9525034732	0.210075207	0.1309257114
O	3.96144211	0.6639275934	2.4467071006
Si	1.0307319508	-0.7444902174	1.188646681
O	3.2536607828	-2.8691345137	3.7812007135
O	1.2655090082	-1.5561273748	2.5888317771
Si	2.0184459195	-1.8154890087	4.041702919
H	1.0807736519	-2.3459226725	5.0348010442
O	2.6187213489	-0.354263441	4.5462206137
Si	4.2770940734	-3.21847982	2.5117389746
H	3.6630493716	-4.2331208569	1.6335303035
H	5.57271528	-3.6560223923	3.0780558779
H	-0.8983220909	4.6079921893	-3.8084913907
H	-7.1816308946	-0.1896828334	-1.3362598543

O	1.7731069782	2.8322939565	0.8541737779
O	-1.3772947363	-3.5320928574	-0.9112096106
O	-0.1310902062	2.5075642862	-0.9365753297
O	-3.0681242301	-1.5384385104	-1.5171993973
Si	1.3079737195	3.2829708028	-0.6752371735
Si	-2.8500910496	-3.179354499	-1.5612611821
H	2.3330031408	2.9271695205	-1.652957729
H	-2.916844776	-3.6715028011	-2.9396450351
O	1.017614583	4.9109171147	-0.7007963604
O	-4.044126548	-3.8526161136	-0.6324957584
Si	0.8770765507	2.1548484461	2.0619610435
Si	-0.5541775276	-3.147119424	0.4651199664
O	0.3204848564	0.6920154315	1.5193325327
O	0.1352260154	-1.651562601	0.1748492106
H	-6.7851754757	0.0156150989	1.08514961
H	-3.1531634981	5.4944642717	-3.3444530399
Si	3.1326379732	1.0432267886	3.8292406996
O	1.8176210584	1.9437856746	3.3849817754
O	0.5514739616	-4.2636794162	0.8717984507
H	3.9759107194	1.8050548083	4.7536773069
Si	-1.9395218685	4.8604911947	-2.7867647263
Si	-6.2708458631	-0.488182801	-0.210705926
Si	-1.7688251099	2.4511914623	-0.9256273632
Si	-3.2406148653	-0.2496794638	-0.5128402511
O	-2.3947800518	3.4235566569	-2.0767130057
O	-4.7998178491	0.2427785771	-0.5096864633
O	-2.2572599507	0.8971416943	-1.1599723692
O	-1.6184130646	-2.9584675082	1.7109472583
O	-0.4044841358	3.0902007191	2.4531036986
O	-2.7797544476	-0.6492627093	1.0124498252
O	-2.3035457642	2.9369984196	0.557342943
Si	-2.8686421618	-1.9395906415	2.0557213622
Si	-1.6452482566	3.9093126226	1.725183436
H	-2.6521751571	4.2642598469	2.7277999336
H	-2.8003852889	-1.4807026797	3.4455195148
Si	-0.2770560885	5.8815666789	-0.3338472638
Si	-5.139025292	-3.3345528003	0.4934472722
O	-1.3229923788	5.8633130985	-1.607491295

O	-6.0585538448	-2.1320929181	-0.1364277514
H	0.169353061	7.2509745019	-0.061047383
O	-6.1354056726	-4.5488191904	0.94049869
O	-4.2976809897	-2.7213806647	1.7820396535
O	-1.0361796811	5.2646227074	1.0036435247
H	-5.7284196866	-5.3883316624	1.1831938193
H	1.3304572721	-4.2588914516	0.2803450333
O	4.2455654754	-2.3298032687	-1.395973567
C	3.7704007161	-0.839680234	-3.9832875291
C	2.9702204373	-2.6964650742	-1.5392691689
C	2.4805147544	-0.6613126674	-3.1550836206
C	2.0778618434	-2.0308992997	-2.5793964234
H	2.0030581309	-2.7474285	-3.4131495051
H	1.078272481	-1.9582161855	-2.1340737592
O	3.8505620569	-1.5643717095	-4.9398621913
O	2.5450492157	-3.6008562948	-0.8554562398
O	2.7090415482	0.3084833098	-2.1622413687
H	2.5172948529	-0.0235756322	-1.2628932895
C	1.4390964079	-0.1114204878	-4.163724436
H	1.8907865055	0.7220739023	-4.7162355388
H	1.1767575492	-0.900933061	-4.8839164137
C	0.1847147676	0.4362353115	-3.5168245715
O	-0.2134899509	1.5660523729	-3.6712032034
O	-0.4407057114	-0.4726709369	-2.7644896584
H	-1.1936621915	-0.0405199068	-2.3025938237
S	5.1491392921	0.1272243105	-3.3448140338
H	4.5273872555	-1.5727787245	-1.9489124971
C	6.4197465586	-0.492021411	-4.4929869829
H	6.5232586245	-1.5809599449	-4.4011469313
H	6.144776735	-0.2390464931	-5.5251428023
H	7.3593195032	0.0044505213	-4.2176505404
H	7.3593195032	0.0044505213	-4.2176505404

SI DFT 045
E(RB3LYP) = -8159.12072551 A.U.
First frequency = 15.9906 cm^{-1}

O	-4.5342459417	-1.622325049	-0.5370852895

O	-2.3527685868	-0.0674917164	-0.5462802146
Si	-4.0058828743	-0.0320490618	-0.4966777773
H	-4.5473009158	0.6742586584	0.6544038985
O	-4.5553034302	0.7034407296	-1.8778062046
Si	-1.0471328908	-0.1195482404	-1.5561867058
O	-3.7354786595	-2.1977263334	-3.0959712767
O	-1.4593213229	-0.7930417164	-2.9950199198
Si	-2.7427202889	-1.1953109603	-3.9462193095
H	-2.3002256854	-1.8364335944	-5.1860991866
O	-3.5506662372	0.2161648917	-4.2916721153
Si	-4.6378714981	-2.7174704956	-1.8185134755
H	-4.1201478796	-4.0041110069	-1.3083781379
H	-6.0557301493	-2.799317374	-2.241577451
H	1.7966743669	4.6907193315	4.320856982
H	7.0860969661	0.2122800764	1.3114060899
O	-2.3533734274	2.8698476109	-0.3540033541
O	1.3069802756	-3.1181439603	0.1724090991
O	-0.2492872349	2.0933091895	1.1230685608
O	2.8246979952	-1.0548905582	0.9486509272
Si	-1.7800099857	2.7399181955	1.1952728745
Si	2.6650812283	-2.7029649401	1.0154695718
H	-2.6466258153	1.9119776957	2.0318991152
H	2.5195493153	-3.1382701186	2.4078187161
O	-1.6662287445	4.267882724	1.8210318767
O	3.9961343128	-3.3790036217	0.312999853
Si	-1.6679527493	2.7319492794	-1.8536442999
Si	0.6629072684	-2.5493233668	-1.2500623912
O	-0.6211241659	1.4503925594	-1.8252164126
O	0.1776526667	-0.98486082	-0.9237428394
H	6.8762457887	0.4635071534	-1.1272334265
H	3.0415466074	5.6383771201	2.4249413061
Si	-4.084268612	1.4211401397	-3.2916260668
O	-2.8511077063	2.4822478459	-2.9560905049
O	-0.4986100523	-3.5203089731	-1.8317748859
H	-5.1939713479	2.1458184874	-3.9165522335
Si	1.8368187197	4.8894623399	2.8536275803
Si	6.2620161589	-0.0652248742	0.115117416
Si	1.2582466226	2.6723859621	0.7810186469

Si	3.2240262975	0.2302488907	-0.0059256818
O	1.8360840821	3.3822647816	2.1477840229
O	4.7743330103	0.6526686424	0.3274684572
O	2.2375538719	1.4652926625	0.3446850067
O	1.8626418796	-2.4234434439	-2.3743643894
O	-0.8135971467	4.0923769706	-2.1823017977
O	3.1164450401	-0.1836218923	-1.5910659316
O	1.1802968249	3.81008277	-0.4025408203
Si	3.2174649652	-1.5092738962	-2.5817902736
Si	0.2640572304	4.9145089594	-1.2281128247
H	1.1050651244	5.7718492367	-2.0669008761
H	3.3630853332	-1.0961363557	-3.9799266321
Si	-0.9096538352	5.7102120332	1.5149425455
Si	5.2199053309	-2.9257308843	-0.7059696089
O	0.4962730854	5.7436159519	2.3762582389
O	6.0691548464	-1.7074577092	-0.0150958325
H	-1.7656663246	6.8345054168	1.9047534815
O	6.2507712879	-4.1652661241	-0.9694416809
O	4.5391185204	-2.3885261503	-2.1137172567
O	-0.5585735699	5.8053918989	-0.1027856551
H	5.8816832397	-4.9751869133	-1.3396168574
H	-1.0728843535	-3.9789407047	-1.1785956128
O	-1.4814417531	-2.4827643876	0.7797274223
C	-0.4271472998	-2.1379354167	3.9038896465
C	-1.5755639145	-3.7485038141	1.1795009118
C	-1.5888012787	-3.1774782181	3.8359539232
C	-1.3562241931	-4.1295820322	2.62859416
H	-1.9769877724	-5.0180353309	2.8043773967
H	-0.3084884969	-4.4722542776	2.685082605
O	0.6086671065	-2.4840485314	4.4306620544
O	-1.788077292	-4.6001238892	0.3420753044
O	-1.4483224381	-3.9653498892	4.997816091
H	-0.4976756599	-3.9756115204	5.2056782614
C	-3.0186646708	-2.5992036373	3.9351238733
H	-3.6463647732	-3.4453135029	4.245454286
H	-3.0320716772	-1.8407343805	4.7282900113
C	-3.6071523808	-1.9954546866	2.6799799111
O	-3.5390355703	-0.8189807341	2.3981536055

O	-4.1898234133	-2.900513576	1.896845468
H	-4.427514828	-2.4567411554	1.0429157138
S	-0.6496714775	-0.5306781294	3.1823422152
H	-1.1584602818	-1.8271177353	1.4333018614
C	1.0150780911	0.1507664179	3.4538976886
H	1.3788747502	-0.1972368041	4.4279700337
H	1.6896437982	-0.1741130254	2.6550802481
H	0.9080695951	1.2409129069	3.440020251
H	0.9080695951	1.2409129069	3.440020251

References

[1] Gayraud, O, Courbière, B, & Guégan, F. (2024) *Phys. Chem. Chem. Phys.* **26**, 25780–25787.

[2] Pracht, P, Bohle, F, & Grimme, S. (2020) *Phys. Chem. Chem. Phys.* **22**, 7169–7192.

[3] Meli, R & Biggin, P. C. (2020) *J. Cheminform.* **12**, 49.

[4] Grimme, S, Antony, J, Ehrlich, S, & Krieg, H. (2010) *J. Chem. Phys.* **132**, 154104.

[5] Grimme, S, Ehrlich, S, & Goerigk, L. (2011) *J. Comput. Chem.* **32**, 1456–1465.

[6] Frisch, M. J, Trucks, G. W, Schlegel, H. B, Scuseria, G. E, Robb, M. A, Cheeseman, J. R, Scalmani, G, Barone, V, Petersson, G. A, Nakatsuji, H, Li, X, Caricato, M, Marenich, A. V, Bloino, J, Janesko, B. G, Gomperts, R, Mennucci, B, Hratchian, H. P, Ortiz, J. V, Izmaylov, A. F, Sonnenberg, J. L, Williams-Young, D, Ding, F, Lipparini, F, Egidi, F, Goings, J, Peng, B, Petrone, A, Henderson, T, Ranasinghe, D, Zakrzewski, V. G, Gao, J, Rega, N, Zheng, G, Liang, W, Hada, M, Ehara, M, Toyota, K, Fukuda, R, Hasegawa, J, Ishida, M, Nakajima, T, Honda, Y, Kitao, O, Nakai, H, Vreven, T, Throssell, K, Montgomery, Jr., J. A, Peralta, J. E, Ogliaro, F, Bearpark, M. J, Heyd, J. J, Brothers, E. N, Kudin, K. N, Staroverov, V. N, Keith, T. A, Kobayashi, R, Normand, J, Raghavachari, K, Rendell, A. P, Burant, J. C, Iyengar, S. S, Tomasi, J, Cossi, M, Millam, J. M,

Klene, M, Adamo, C, Cammi, R, Ochterski, J. W, Martin, R. L, Morokuma, K, Farkas, O, Foresman, J. B, & Fox, D. J. (2016) Gaussian˜16 Revision B.01. Gaussian Inc. Wallingford CT.

[7] (2020) "cylview20; legault, c. y., université de sherbrooke, 2020" (http://www.cylview.org). Accessed: 2024-06-27.

[8] Rimola, A, Fabbiani, M, Sodupe, M, Ugliengo, P, & Martra, G. (2018) *ACS Catalysis* **8**, 4558–4568.
